# Autolysosomal enhancer GHF201 attenuates pathophysiology in a mucopolysaccharidosis type I mouse model

**DOI:** 10.26508/lsa.202603750

**Published:** 2026-10-06

**Authors:** Pradeep Kumar, Emmanuelle Merquiol, Talya Avraham, Reut Sinai-Turyanski, Pankaj K Singh, Roberto A Ribas, Sahar Sweetat, Uri Sprecher, Noam Muchtar, Manjeet Gupta, Kumudesh Mishra, Shashank Kumar Ojha, Zhanna Yekhtin, Hava Glickstein, Ori Shalev, Alexandra Eliassaf, Anna Morshina, Hadas Segev-Yekutiel, Sharon Garti, Nissim Garti, Hannah Rosenmann, Miguel Weil, Matthew S Gentry, Galia Blum, Or Kakhlon

**Affiliations:** 1 Department of Neurology, The Agnes Ginges Center for Human Neurogenetics, Hadassah Hebrew University Medical Center, Jerusalem, Israel; 2 Faculty of Medicine, Hebrew University of Jerusalem , Ein Kerem, Jerusalem, Israel; 3 The Institute of Drug Research, School of Pharmacy, Faculty of Medicine, The Hebrew University of Jerusalem , Jerusalem, Israel; 4 Department of Biochemistry and Molecular Biology, College of Medicine, University of Florida, Gainesville, FL, USA; 5 Center for Advanced Spatial Biomolecule Research, University of Florida, Gainesville, FL, USA; 6 The Shmunis School of Biomedicine and Cancer Research, The George S. Wise Faculty for Life Sciences, Tel Aviv University , Tel Aviv, Israel; 7 Departments of Bone Marrow Transplantation and Pulmonology, Hadassah Hebrew University Medical Center, Jerusalem, Israel; 8 Electron Microscopy Unit, The Hebrew University-Hadassah Medical School, Ein Kerem, Jerusalem, Israel; 9 The Metabolic Profiling Unit, Core Research Facility, Faculty of Medicine, The Hebrew University of Jerusalem , Jerusalem, Israel; 10 Flow Cytometry Unit, The Core Research Facility, Faculty of Medicine, The Hebrew University of Jerusalem , Jerusalem, Israel; 11 Lyotropic Delivery Systems Ltd., Jerusalem, Israel; 12 Sagol School of Neurosciences, Tel Aviv University , Tel Aviv, Israel

## Abstract

Mucopolysaccharidosis type I (MPS-I) is a hereditary lysosomal storage disorder caused by deficiency in the lysosomal enzyme α-L-iduronidase (IDUA) leading to pathogenic over-accumulation of glycosaminoglycans (GAGs), major constituent of connective tissue and mediators of cell signaling. We tested the therapeutic efficacy of the autolysosomal activator GHF201 in the *Idua*^*−/−*^ mouse model of MPS-I. We show that GHF201 reduced GAGs and increased cathepsin activity in *Idua*^*−/−*^ mouse embryonic fibroblasts (MEFs) and brain cells. In vivo GHF201 improved aberrant locomotion and behavior, reduced interstitial cathepsins in joints and liver, increased systemic catabolism, corrected bone dysplasia, increased autophagic flux, and ameliorated CNS neurodegeneration and neuroinflammation. GHF201 also reduced brain N-glycans, which are degraded in lysosomes. In conclusion, the lysosomal and autophagic activator GHF201 was demonstrated to improve various pathophysiological parameters in MPS-I–modeling *Idua*^*−/−*^ female mice. Our results suggest that GHF201 can complement the inadequate Aldurazyme enzyme replacement therapy as an add-on therapy.

## Abstract (precis)

This work demonstrates that the autolysosomal enhancer GHF201 attenuates pathophysiology in an MPS-I mouse model.

## Graphical Abstract

**Figure:**
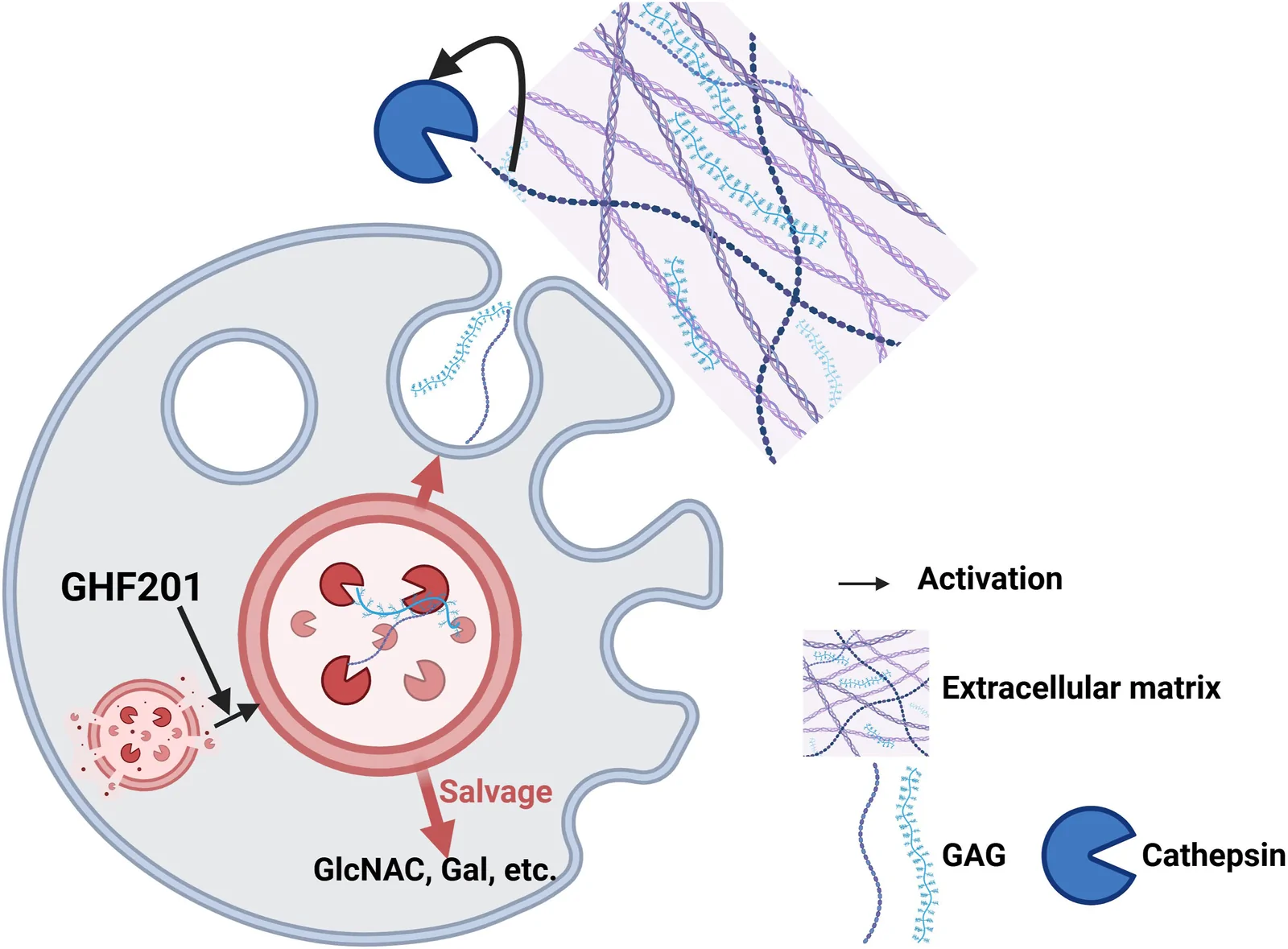

## Introduction

Mucopolysaccharidoses (MPSs) are a subgroup of lysosomal storage disorders caused by deficiencies in lysosomal enzymes responsible for the breakdown of glycosaminoglycans (GAGs) (1). MPS type I (MPS-I), the most common and severe MPS, is caused by a deficiency in iduronidase (Idua), leading to lysosomal storage of GAGs, dermatan sulfate (DS) in the periphery (2), and heparan sulfate (HS) in the central nervous system (CNS) (3). Idua deficiency in MPS-I leads to lysosomal and extracellular (4, 5) GAG accumulation and to lysosome swelling and malfunction.

GAG chains, especially those linked to proteoglycans, play a critical role in the assembly of extracellular matrix (ECM) and connective tissues and thus in the development and function of all organs. Their dysregulation, as happens in MPS-I, is therefore multifaceted involving the entire body. Accumulation of extracellular GAGs (6) in MPS-I causes overactivation of extracellular cathepsins either directly (7, 8) or via activation of macrophages (9), which subsequently secretes cathepsins. Dysregulated extracellular cathepsin activity, in turn, enhances amyloid production (10) and collagen overdigestion (9). After excessive lysosomal GAG storage in MPS-I, the lysosomes become distended and dysfunctional causing accumulation of toxic aggregates, abnormal vesicle fusion with ensuing impairment of intracellular trafficking (11, 12), compromised autophagy in a vicious cycle (13), and, subsequently, unregulated oxidative stress because of impaired mitophagy.

MPS-I has a global prevalence of approximately 1 in 100,000 live births, with the severe Hurler phenotype (MPS-I-H) outnumbering the attenuated Scheie variant by a 3:1 ratio (14). Pathologically, this dominant, severe form presents as a multisystemic disorder characterized by prominent peripheral features such as corneal clouding, upper airway obstruction, progressive cardiomyopathy, restrictive joint contractures, and chronic systemic inflammation (15, 16). In the attenuated form, Scheie syndrome (MPS-I-S), these symptoms’ onset is delayed by 3–5 yr. CNS pathology in MPS-I-H causes cognitive decline and developmental delays (17). The etiology of the CNS pathology in MPS-I is no different, in principle, from that of other tissues and involves neuroinflammation by GAG-mediated activation of Toll-like receptor 4 (TLR4) (18, 19), and the mentioned mitochondrial, lysosomal, and autophagic dysfunctions. The neurological and retinal symptoms in MPS-I are treated less effectively than other symptoms because of the restricted ability of Idua enzyme replacement therapy (ERT) to cross blood–brain and blood–retina barriers (20). Additional causes for the limited response to ERT are immune reaction to the injected enzyme and disruption of its lysosomal delivery because of autophagic buildup.

Although Idua ERT is irreplaceable as a standard of care for MPS-I, it is insufficient as a monotherapy even when started at infancy after diagnosis through newborn screening. Although ERT for MPS-I is effective (21, 22), alleviation of symptoms is incomplete and inconsistent. Particularly, poor peripheral responses are those of cardiac, respiratory, and ocular symptoms (23, 24). The other common therapeutic approach, heterologous stem cell therapy (HSCT) (25), also does not cure MPS-I and is an invasive procedure, potentially more detrimental in fragile infants with MPS-I. Moreover, HSCT exposes the patients to a preconditioning immune suppression regimen, which increases infection and malignancy risks and is inefficient in reversing preexisting MPS-I pathology (26). Perhaps most importantly, both ERT and HSCT are not CNS-permeable and thus neither addresses the neuropathology in MPS-I, which arguably is the most debilitating aspect of the disease. Thus, alternative therapies addressing the current limitations of ERT and HSCT have the potential to provide a major beneficial impact for patients.

To address these challenges, we tested the therapeutic efficacy of the novel lysosomal activator GHF201 (27), dosed as a tailored nanodroplet (ND) formulation in *Idua*^*−/−*^ mouse and cell models of MPS-I. The rationale of testing GHF201 in MPS-I models was that we expected it to enhance lysosomal function and autophagic flux, thus enhancing lysosomal GAG turnover. Beyond its clinical safety and efficacy in the treatment of adult polyglucosan body disease patients, GHF201 has improved general and GAG-degrading lysosomal activity in *Idua*^*−/−*^ mice and mouse embryonic fibroblasts (MEFs). Our ex vivo data show that glycans, vacuolation and storage in different tissues, astroglial and microglial immune activation in brain sections, splenomegaly, and urine GAGs were all reduced by GHF201. Plasma biomarkers show that GHF201 treatment reduced neurofilament accumulation and generated a shift toward catabolism, as demonstrated by plasma metabolomics analysis. Lysosomal activity in microglia and neurons, and autophagic activity in the brain and liver were also improved by GHF201. Our in vivo results also show amelioration of various parameters by GHF201: estimating skeletal features using CT scanning, we show that GHF201 ameliorated bone dysplasia in joints, which is a major factor in MPS-I morbidity. Animal cognition tested by open-field habituation and Morris water maze (MWM) tests was improved, as was animal motility assessed by open-field and grip-strength tests. Our in vivo imaging system (IVIS) results show that GHF201 can reduce overall cathepsin activity in joints and in liver, which is considered a therapeutic advantage considering the excessive cathepsin activity observed in these tissues in *Idua*^*−/−*^ mice. In summary, we believe that this comprehensive analysis demonstrates safety and efficacy of the lysosomal activator GHF201 as an MPS-I potential therapeutic, warranting its clinical development as an adjunct treatment for this incurable disorder.

## Results

### GHF201 and ERT reduce GAG levels in MEFs

**Figure 1.**
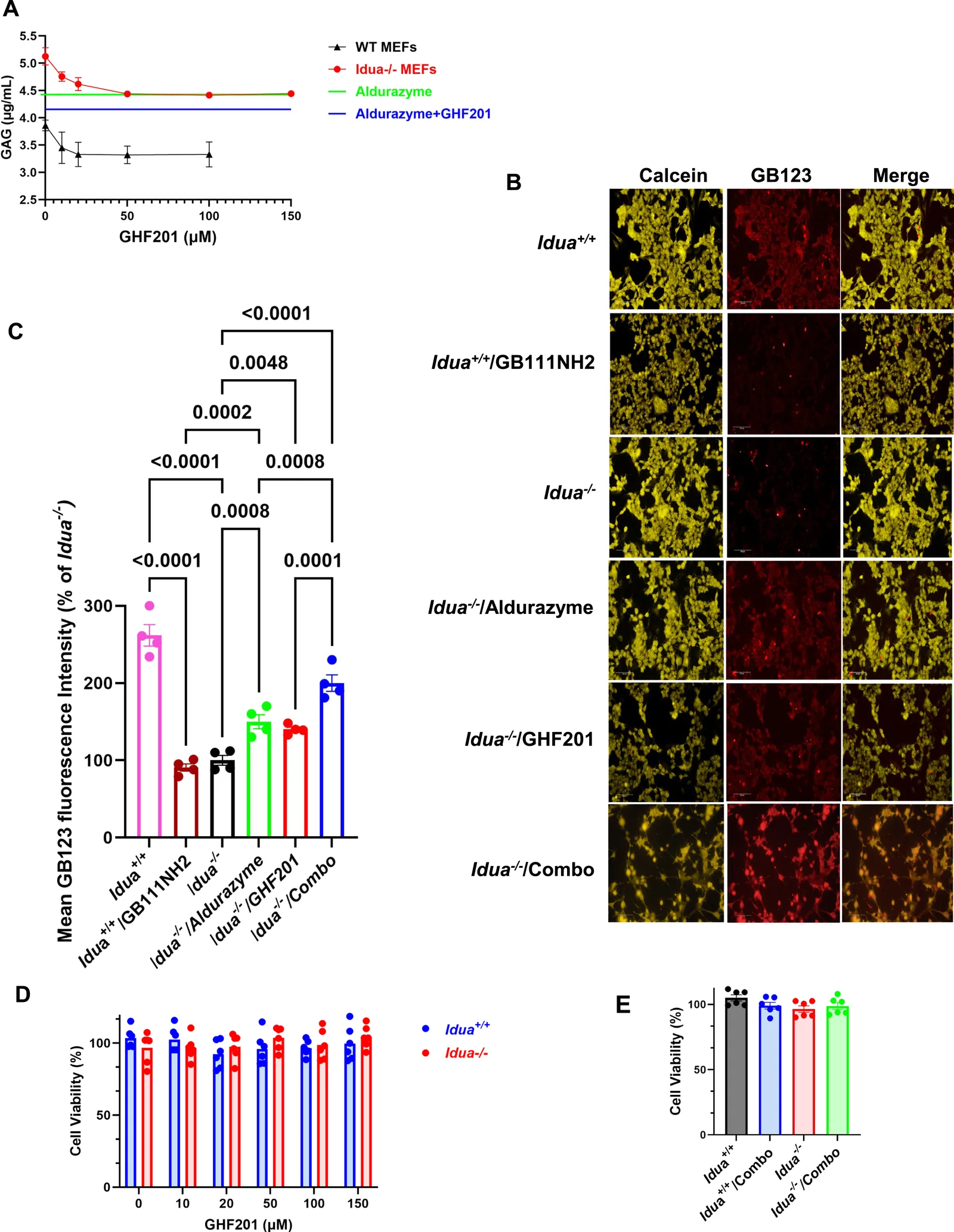
GAG and cathepsin activity in MEFs. **(A)** *Idua*^*+/+*^ and *Idua*^*−/−*^ MEFs were incubated in the absence or presence of increasing GHF201 concentrations for 24 h. The green line shows the sGAG level (quantified by Blyscan) in *Idua*^*−/−*^ MEFs treated with 5 μg/ml Aldurazyme, and the blue line shows *Idua*^*−/−*^ MEFs treated with a combination of 5 μg/ml Aldurazyme and 150 μM GHF201. These treatments also lasted 24 h. The data are shown as the mean ± SD of MEFs derived from four pregnant mice (5 embryos each) per group. **(B)** *Idua*^*+/+*^ or *Idua*^*−/−*^ MEFs, as indicated, were incubated for 24 h alone, or in the presence of the cathepsin inhibitor GB111-NH2 (1 μM), the ERT Aldurazyme (Idua, 5 μg/ml), GHF201 (50 μM), or a combination of 50 μM GHF201 and 5 μg/ml Aldurazyme (combo). MEFs were then stained for 1 h with 0.25 μM of the red fluorescence cathepsin activity probe GB123 and 1 nM Hoechst followed by live microscopy imaging. Scale bars, 10 μm. **(C)** GB1232 fluorescence intensity of the indicated groups. *P*-values were calculated by one-way ANOVA with Tukey’s post hoc test. **(D)** Assessment of GHF201 toxicity by the MTT assay. Relative cell survival of MEFs derived from *Idua*^*+/+*^ and *Idua*^*−/−*^ pregnant females treated for 24 h with the indicated concentrations of GHF201 as in (A). **(E)** Assessment of the toxicity of the Aldurazyme+GHF201 combination (combo) using the same conditions as in (D).

GHF201 is our own-discovered lysosomal activator (27). Therefore, it was expected to improve lysosomal function in all cells, healthy and affected, but probably more in affected ones where lysosomes would be more liable to therapeutic intervention. To evaluate GHF201’s capacity to reduce intracellular sulfated GAGs (sGAGs), we used Blyscan’s sGAG quantification assay (Biocolor; e.g., reference (28)) across a fixed concentration gradient. Primary MEFs were derived from five distinct embryos per litter from four pregnant *Idua*^*−/−*^ or *Idua*^*+/+*^ littermate control mice. *Idua*^*+/+*^ cells were treated with 0, 10, 20, 50, and 100 μM GHF201, whereas mutant *Idua*^*−/−*^ cells received an identical gradient supplemented with an additional 150 μM treatment group. This a priori selected 150 μM dose served as a saturating, supramaximal concentration to ensure the absolute therapeutic plateau of GHF201 had been reached in the affected cells. This configuration permitted a rigorous evaluation of GHF201’s maximal efficacy when benchmarked directly against the efficacy ceiling of recombinant Idua ERT. GHF201 lowered GAGs in a dose-responsive manner in both MEF populations (Fig 1A). Expectedly, sGAG levels in *Idua*^*−/−*^ MEFs were significantly higher than those in *Idua*^*+/+*^ MEFs. Supplemented 5 μg/ml Idua (Ald, Aldurazyme, recombinant Idua) served as a positive control for GAG degradation. Treatment with the enzyme alone significantly reduced, but did not normalize, GAG levels, possibly because of limiting surface cation-independent mannose-6-phosphate receptor availability, preexisting disruption of endo-lysosomal fusion in the affected *Idua*^*−/−*^ MEFs, or sGAG substrate aggregation and ensuing inaccessibility. On the other hand, GAG levels in Idua-supplemented *Idua*^*−/−*^ MEFs were reduced to levels equivalent to those attained by treatment with > 50 μM GHF201. Supplementation of a combination of GHF201 (150 μM) and Ald (5 μg/ml) additively reduced sGAG levels to 4.15 μg/ml.

### GHF201 increases cathepsin activity additively with ERT in *Idua*^−/−^ mouse fibroblasts

To test whether GHF201 reduced GAG accumulation by activating lysosomes, and whether its activity has an additive effect to ERT, *Idua*^*−/−*^ MEFs were treated with GHF201 in the absence or presence of Ald and lysosomal cathepsin activity was quantified using the fluorescent activity–based probe GB123, which binds irreversibly to active cathepsins (29) (Fig 1B). As expected, lysosomal activity in *Idua*^*−/−*^ MEFs, probed as a red fluorescent signal, was significantly lower (38 ± 5%) than in *Idua*^*+/+*^ MEFs (Fig 1B, quantified in Fig 1C). This reduction in GB123 intensity was not caused by GHF201 cytotoxicity (Fig 1D), or by toxicity of the Ald+GHF201 combination (combo, Fig 1E), GHF201 and Ald increased lysosomal cathepsin activity to 57 ± 7 and 54 ± 2% of *Idua*^*+/+*^ activity, respectively. Their combination further improved lysosomal activity to 76 ± 8%, significantly above each drug alone. These results suggest that the reduction in GAG accumulation upon treatment with GHF201 (Fig 1A) was indeed due to activation of lysosomal function, as reported by the increased lysosomal cathepsin activity, and the use of GHF201 together with Ald as a combination therapy.

### A tailored ND formulation of GHF201 improves its pharmacokinetic profile

**Figure S1.**
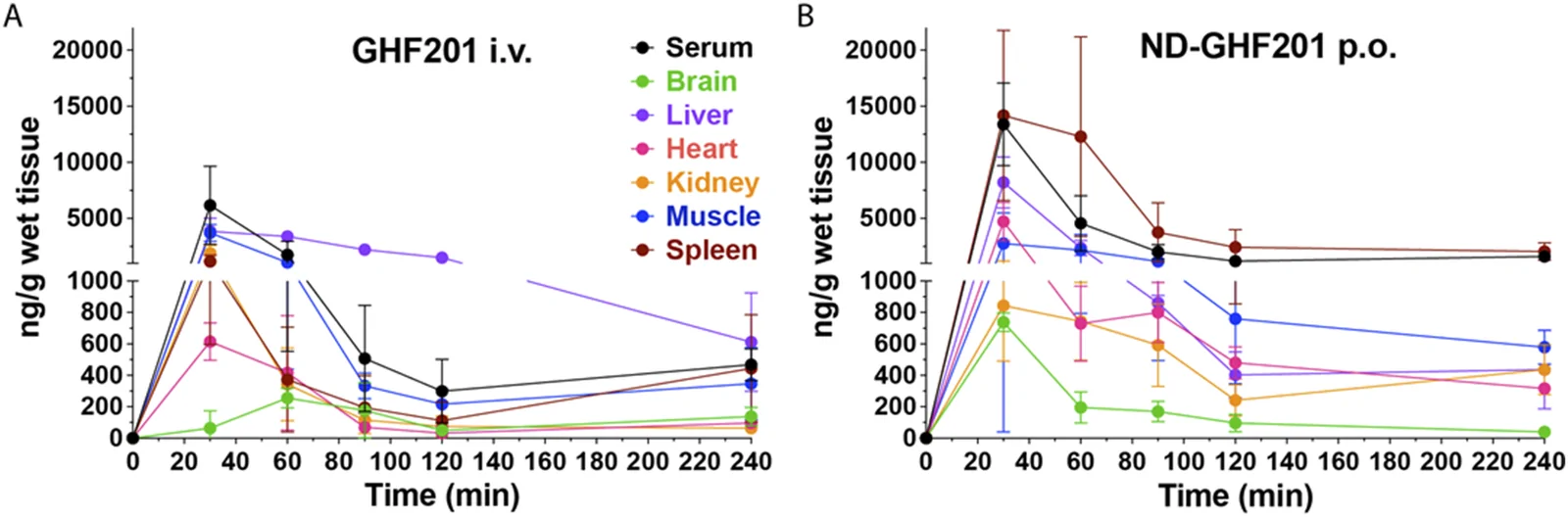
Nonaqueous oral solution ND formulation improves oral absorption and BBB penetration of GHF201. 70 mg/kg GHF201 was administered (A) i.v. in 5% DMSO, or (B) p.o. as ND-GHF201 to Idua−/− mice. Animals were euthanized 30, 60, 90, 120, or 240 min post-administration, and the indicated tissues were extracted for PK analysis by LC-MS/MS. The data are presented as the mean ± SD of three mice per time point. BBB, blood–brain barrier.

With the intent of increasing biodistribution and efficacy, in our in vivo and ex vivo experiments we used a tailored nanoformulation of GHF201. After its initial use as an intravenous (i.v.) injectable drug (27), this new ND nonaqueous oral solution formulation of GHF201 for per os (p.o.) administration has been developed by Lyotropic Delivery Systems Ltd. To test the formulation, ND-GHF201 was administered p.o. to *Idua*^*−/−*^ mice and found to have a substantially slower clearance rate (serum AUC = 784,000, Fig S1B) compared with unformulated GHF201 (AUC = 303,000, Fig S1A). Importantly, the brain penetration of ND-GHF201 (AUC = 42,700, Fig S1B) also was substantially higher than that of GHF201 administered i.v. (AUC = 26,900, Fig S1A).

### GHF201 improves behavioral and motor deficits in *Idua*^−/−^ mice

**Figure 2.**
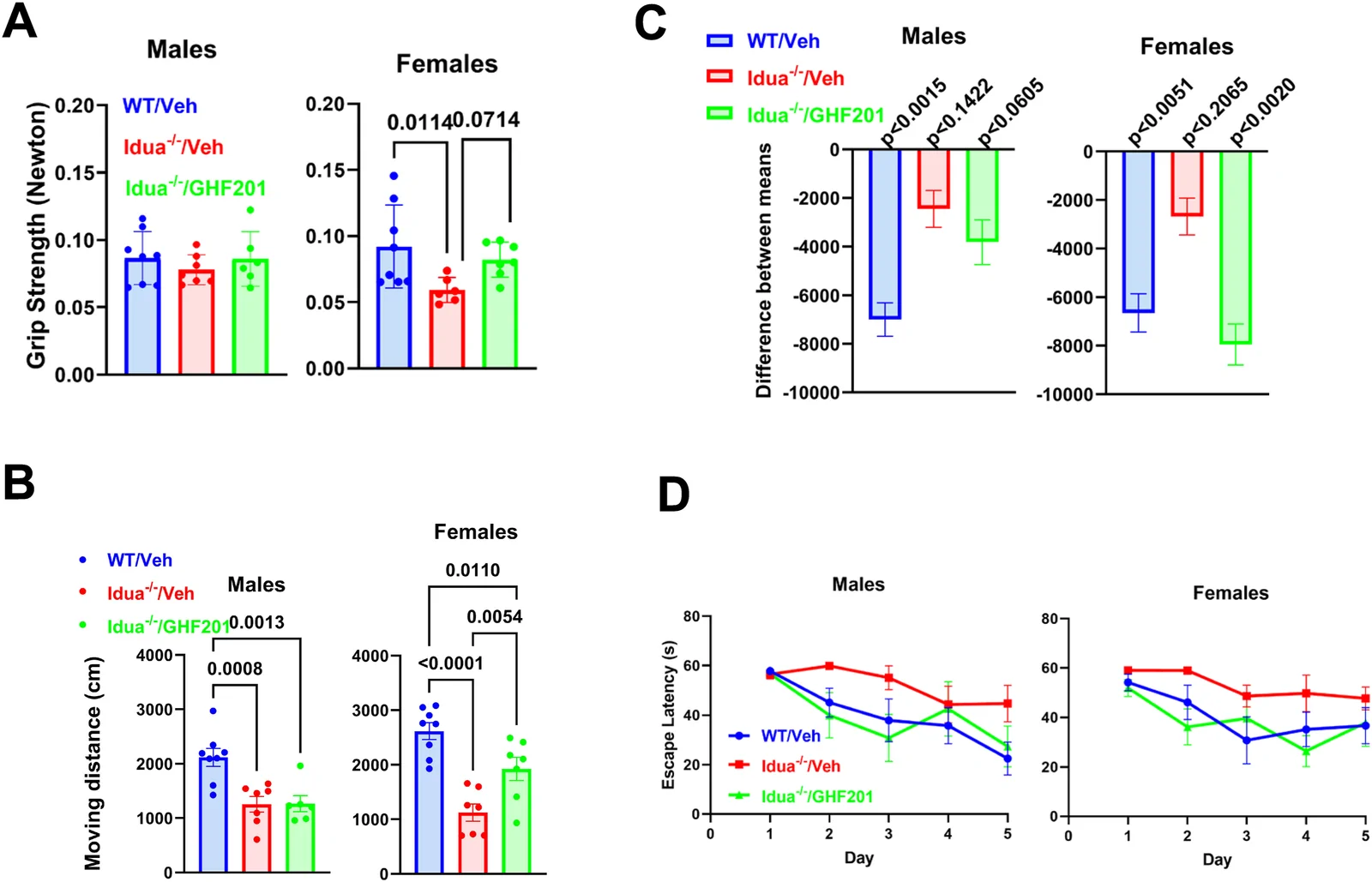
Effects of GHF201 on motor performance and behavior. **(A)** Grip strength in male and female mice was measured in (left panel) n = 8 *Idua*^*+/+*^ males, n = 7 *Idua*^*−/−*^ males, and n = 6 GHF201-treated *Idua*^*−/−*^ males, as well as in (right panel) n = 8 *Idua*^*+/+*^ females, n = 6 *Idua*^*−/−*^ females, and n = 7 GHF201-treated *Idua*^*−/−*^ females. Control animals were treated with unloaded ND vehicle (*Idua*^*+/+*^/Veh and *Idua*^*−/−*^/Veh, respectively). One-way ANOVA with correction for multiple comparisons shows that GHF201 corrected the decrease in grip strength observed in *Idua*^*−/−*^ female mice, as compared to *Idua*^*+/+*^. **(B)** Open-field moving distance was measured in (left panel) n = 8 *Idua*^*+/+*^ males, n = 7 *Idua*^*−/−*^ males, and n = 6 GHF201-treated *Idua*^*−/−*^ males, as well as in (right panel) n = 8 *Idua*^*+/+*^ females, n = 7 *Idua*^*−/−*^ females, and n = 7 GHF201-treated *Idua*^*−/−*^ females. One-way ANOVA with correction for multiple comparisons shows that GHF201 corrected the decrease in movement in an open-field arena observed in *Idua*^*−/−*^ female mice, as compared to *Idua*^*++/*^ mice. **(C)** Open-field habituation mean differences and their respective *P*-values are shown for the same male and female mice analyzed in (B). Open-field habituation evaluates long-term nonassociative (i.e., same stimulus) spatial memory—the higher the difference the better the nonassociative spatial memory. Open-field habituation was improved by GHF201 in *Idua*^*−/−*^ females more than in *Idua*^*−/−*^ males. **(D)** The MWM tests spatial learning by quantifying the ability to rely on distal cues to navigate to a submerged platform, which was determined as a reference memory when the platform was exposed at the water surface. The figure shows the change over time in latency to escape to a submerged platform as an inverse estimate of spatial learning. This escape latency was measured over 5 d in n = 8 *Idua*^*+/+*^, n = 7 *Idua*^*−/−*^, and n = 7 GHF201-treated male and female mice. A one-way repeated-measures ANOVA revealed that the progressive decrease in escape latency over time, an index of spatial learning, was significantly attenuated in *Idua*^*−/−*^ mice (red) compared with *Idua*^*+/+*^ controls (blue, *P* < 0.03). This learning deficit was significantly improved by GHF201 treatment (*P* < 0.02), restoring performance to a level where the trajectory of treated *Idua*^*−/−*^ mice (green) was statistically indistinguishable from *Idua*^*+/+*^ controls (*P* > 0.05). Visual indicators of these statistical transitions are now explicitly marked on the accompanying line graphs.

**Figure 3.**
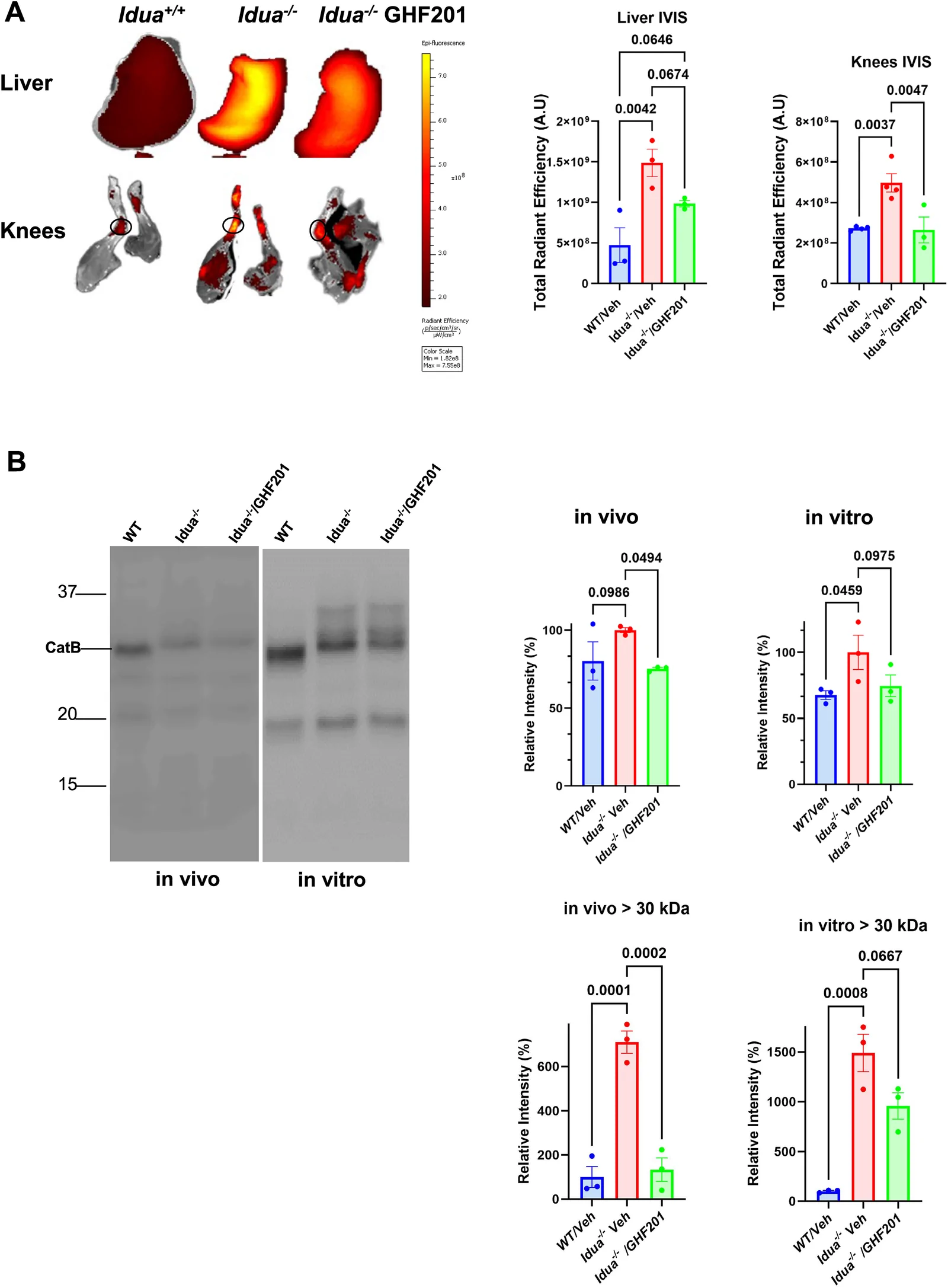
In situ cathepsin activity. **(A)** Left panel, GB123 was injected to female *Idua*^*+/+*^, or *Idua*^*−/−*^ mice with or without 5.5 mo of GHF201 treatment, 24 h before IVIS scanning of tissue ex vivo, liver (upper panel) and knee joints (circles*,* lower panel). Right panel, total radiant fluorescence efficiency was quantified in 3–4 mice per group, presented as the mean ± SD. **(B)** Left panel, liver lysates from mice treated with GB123 in vivo (described in (A)), or liver lysates extracted from the indicated groups that were treated in vitro with 2 μM GB123 and then separated by 12.5% sodium dodecyl sulfate–polyacrylamide gel electrophoresis and visualized on-gel by scanning the GB123 signal. Right panel, on-gel GB123 signal quantification, reflecting total lane densitometry, in three mice per group presented as the mean ± SD. Below these total lane densitometric quantifications, we show densitometric quantifications of the apparently hyperglycosylated cathepsin B species with MW > 30 kD. *P*-values (indicated) of the significance of intergroup differences were calculated using a one-way ANOVA with correction for multiple comparisons. Equal total protein input was loaded across all lanes based on preloading BCA protein determination. Source data are available for this figure.

**Figure 4.**
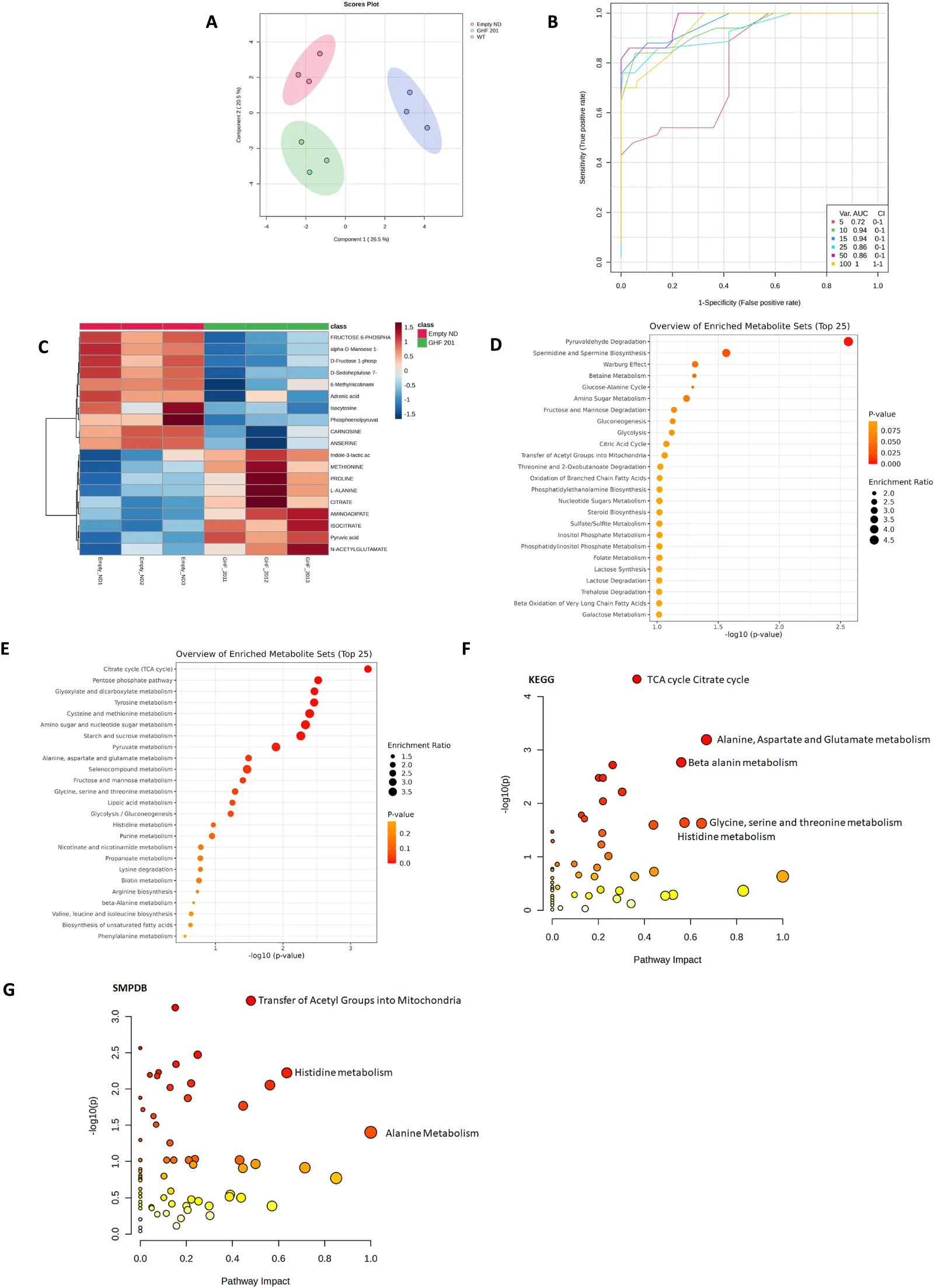
Systemic effects of GHF201. **(A)** PLS-DA showing that the plasma metabolome of GHF201-treated and untreated *Idua*^*−/−*^ mice was distinct from each other and from *Idua*^*+/+*^ mouse plasma metabolome. **(B)** Predictive ROC exploratory curves validating the diagnostic accuracy of selected plasma biomarker panels. A minimized panel of 15 optimal metabolites (blue) successfully classifies *Idua*^*−/−*^ and *Idua*^*+/+*^ mice with a high predictive probability (AUC = 0.94). **(C)** Metabolome heatmap showing 19 plasma metabolites significantly different between *Idua*^*−/−*^ and *Idua*^*+/+*^ mice (*t* test). **(D, E)** Metabolite set enrichment analyses comparing plasma metabolites of ND-GHF201–treated *Idua*^*−/−*^ mice versus empty-ND (i.e., unloaded ND)–treated *Idua*^*−/−*^ mice as a reference. The comparisons were against the KEGG (D) and SMPDB (E) datasets. **(F, G)** Pathway enrichment and topology analyses comparing plasma metabolites of GHF201-treated and untreated *Idua*^*−/−*^ mice against two metabolite datasets, KEGG (F) and SMPDB (G). For both panels, the circle size is proportional to the calculated pathway impact score, and the circle color intensity (shifting from yellow to dark red) corresponds to increasing statistical significance (−log_10_(*P*-value)). The data are presented as the mean ± SD. *P*-values were calculated using a one-way ANOVA with correction for multiple comparisons. AUC, area under the curve; ROC curve, receiver operating characteristic curve. Source data are available for this figure.

**Figure 5.**
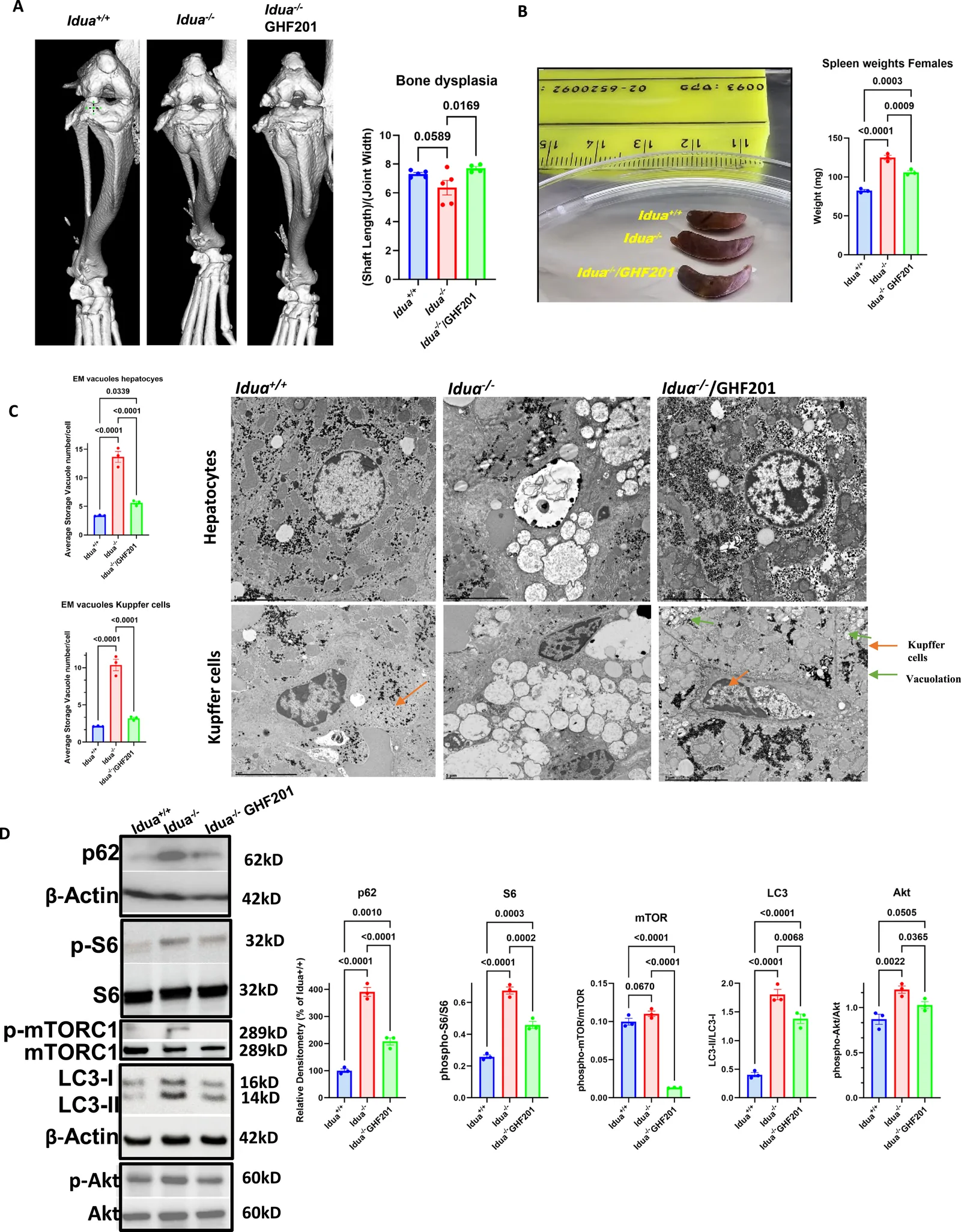
GHF201 effect on joint, spleen, and liver pathology. **(A)** Left panel, representative CT images of legs of *Idua*^*+/+*^ and vehicle and GHF201-treated *Idua*−/− mice illustrating bone dysplasia (especially note knee joint deformity and reduced tibia length-to-joint width ratio) in *Idua*−/− mice and its partial correction by GHF201. Right panel, quantification of (shaft length)/(joint width) as a measure of bone dysplasia. n = 3. *P*-values were determined by one-way ANOVA with correction for multiple comparisons. **(B)** Left panel, a representative photo of the spleens collected from 8-mo-old mice from the indicated genotypes and treatments. Right panel, mean weights of spleens from n = 3 mice in the indicated groups. One-way ANOVA with correction for multiple comparisons shows that spleen weights were significantly increased in *Idua*^*−/−*^ mice and decreased by GHF201. **(C)** Representative TEM micrographs of liver sections showing hepatocytes, identified by cytoplasmic glycogen rosettes, and Kupffer cells (orange arrows). Livers of *Idua*^*−/−*^ mice contain more storage vacuoles (endo-lysosomes and autolysosomes). GHF201 treatment leads to a reduction in the size and number of storage vacuoles. The left panel shows the number of storage vacuoles/cell in the different treatments. Shown are average (±SEM) vacuole counts per animal based on quantification of 10 biospecimens/animal from all animals (n = 3 per group), and statistical significance was determined by one-way ANOVA with correction for multiple comparisons. Scale bars, 5 μm. **(D)** Left panel*,* representative Western immunoblots of autophagic markers in liver extracts from *Idua*^*+/+*^, *Idua*^*−/−*^, and GHF201-treated *Idua*^*−/−*^ mice. Right panel*,* densitometric analysis of immunoblots from n = 3 mice/group. Statistically significant differences were determined by one-way ANOVA with correction for multiple comparisons.

**Figure 6.**
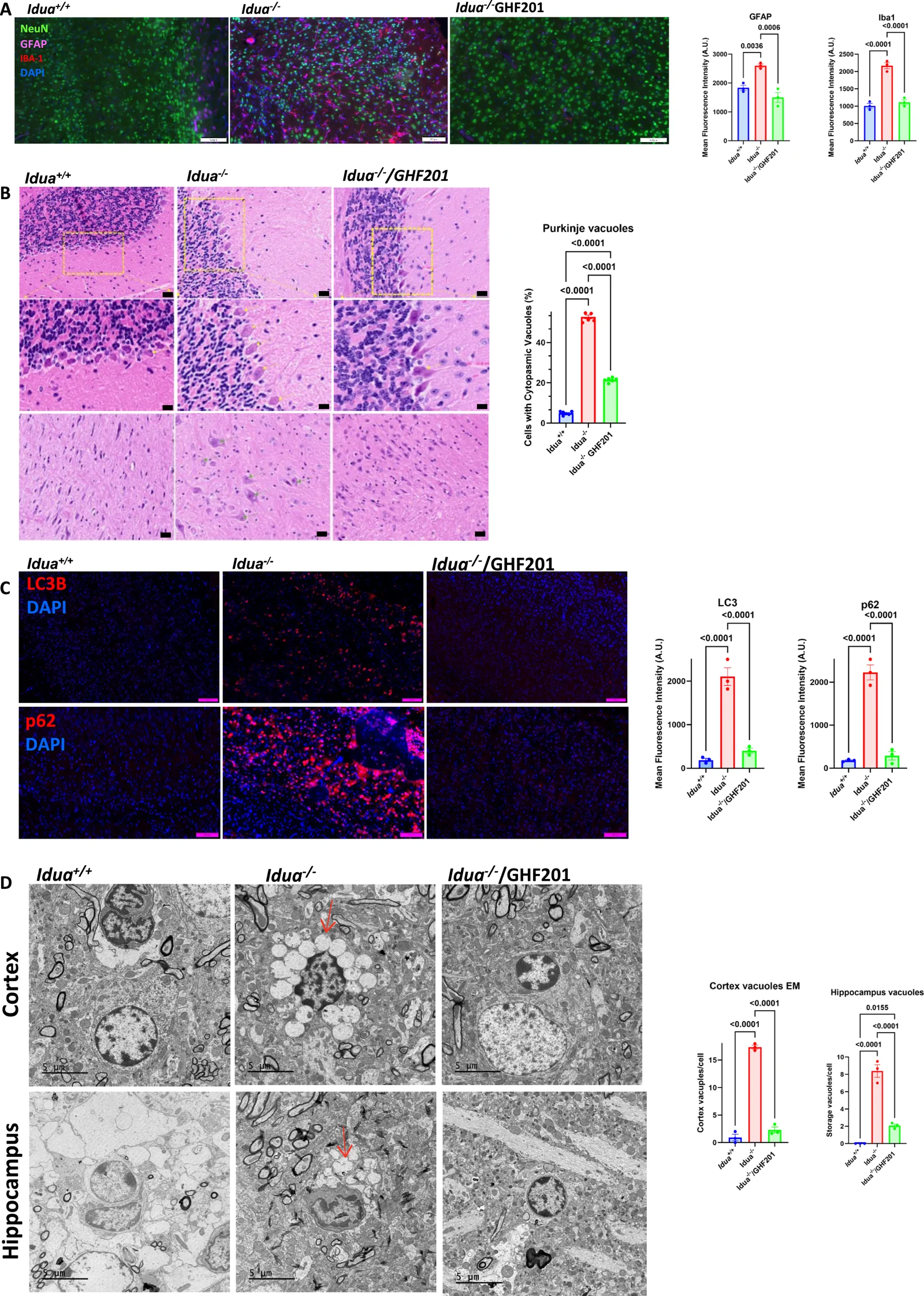
GHF201 effect on neuroinflammation and CNS pathology. **(A)** Left panel, brain slices from the indicated mouse groups were fixed and stained with NeuN and DAPI to detect neuronal and total cell nuclei, respectively. GFAP and Iba1 antibodies were used as biomarkers of gliosis and microglial activation, respectively. Secondary antibodies were conjugated to FITC, CY3, and CY5 dyes to respectively stain NeuN, Iba1, and GFAP as indicated. Right Panel, quantification of fluorescence intensity of GFAP and Iba1 staining in *Idua*^*+/+*^, *Idua*^*−/−*^, and GHF201-treated *Idua*^*−/−*^ mice (n = 3 per group). Statistical differences were determined by one-way ANOVA with multiple comparisons correction. **(B)** Upper l*eft* panel, H&E staining of fixed cerebellum sections taken from mice of the indicated genotypes and treatments. Purkinje cells, shown also in the blowup in the middle left panel, manifest cytoplasmic vacuoles (yellow arrows) as a sign of morphological abnormality and stress. Lower left panel, medulla regions from the same animals also showing vacuolation (green arrows). Right panel, quantification of the percentage of vacuolated Purkinje cells based on five fields from n = 6 animals per group. One-way ANOVA with correction for multiple comparisons shows that cerebral Purkinje cells are hypervacuolated in *Idua*^*−/−*^ mice. This hypervacuolation is partially restored by GHF201. Scale bars, 50 μm (upper left panel), 20 μm (middle left panel), 50 μm (lower left panel). **(C)** Left panel, brain slices from the indicated mouse groups were fixed and stained with the autophagic substrates LC3B (top), or p62 (bottom), and with the nuclear stain DAPI. Secondary antibodies were conjugated to CY3. Slides were then scanned using the Olympus VS200 slide scanner, and fluorescence intensity was quantified with VS200 built-in software. Right panel, quantification of fluorescence intensity of LC3B and p62 staining. Reduced levels of both LC3B and p62 indicate increased autophagic flux because of enhanced degradation of these autophagic substrates. Scale bars in (A, B, C), 100 μm. **(D)** Left panel, representative TEM micrographs of cortical and hippocampal sections showing resident microglia in the indicated mouse groups. Cortical and hippocampal sections from *Idua*^*−/−*^ mice contain more storage vacuoles (arrows). GHF201 treatment led to a reduction in the size and number of storage vacuoles. Right *panel*, the number of storage vacuoles/cell in the different treatments. Vacuole count was performed across biospecimens from all animals (n = 3 per group), and statistical significance was determined by one-way ANOVA with correction for multiple comparisons. Scale bars, 5 μm.

**Figure 7.**
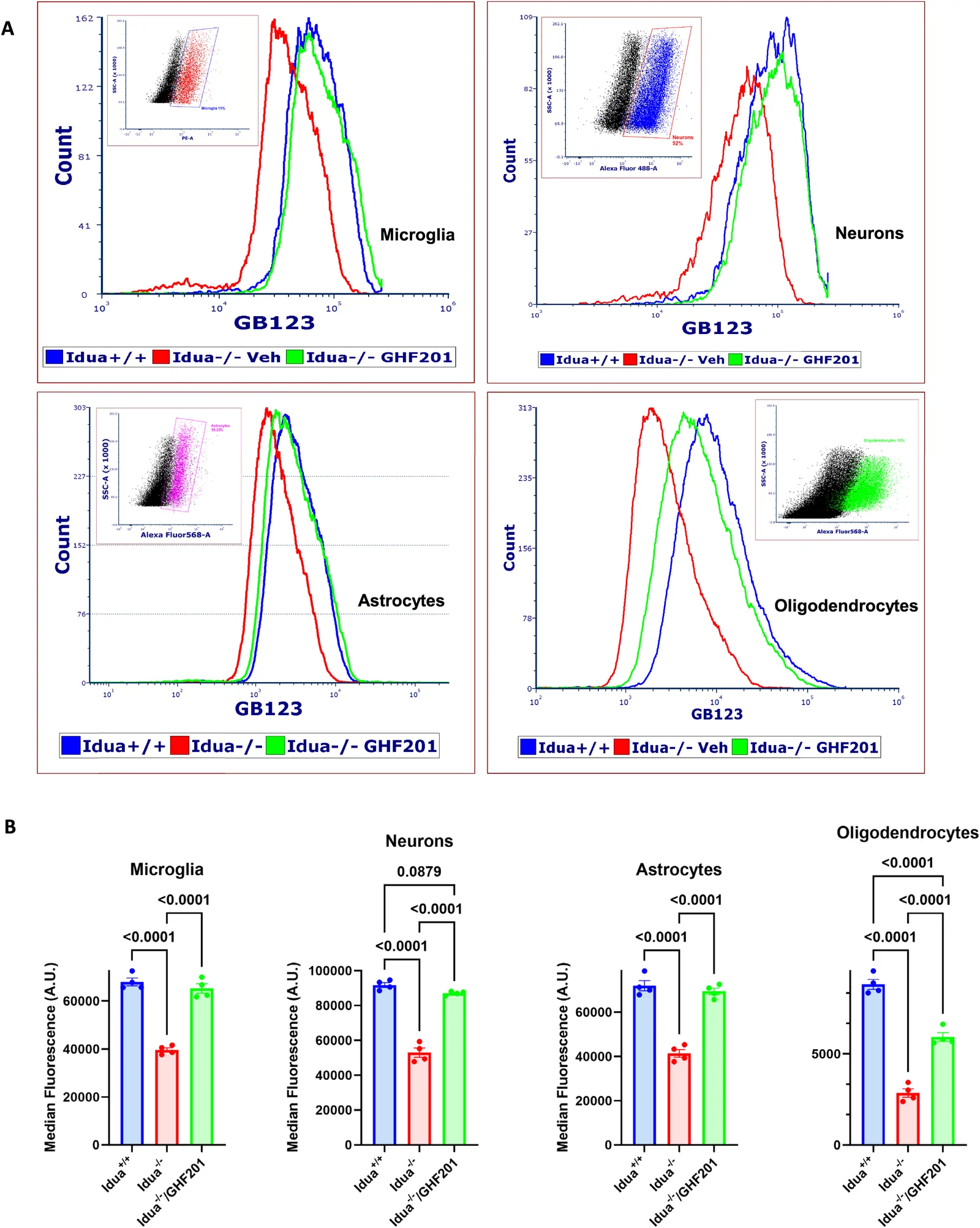
Flow cytometry analysis of cathepsin activity in different brain cell types. **(A)** Representative histograms showing flow cytometry quantification of GB123 fluorescence intensity (cathepsin activity) in the brain cell types specified. Groups are indicated below each histogram. Insets, gates of the cell types analyzed showing their distinct staining by the respective antibodies within the whole-brain cell population. Histograms show gated analysis of these stained subpopulations. **(B)** Quantification of GB123 in microglia, neuronal nuclei, astrocytes, and oligodendrocytes in *Idua*^*+/+*^, *Idua*^*−/−*^, and GHF201-treated *Idua*^*−/−*^ mice (n = 3 per group). Statistical differences were determined by one-way ANOVA with multiple comparisons correction.

**Figure 8.**
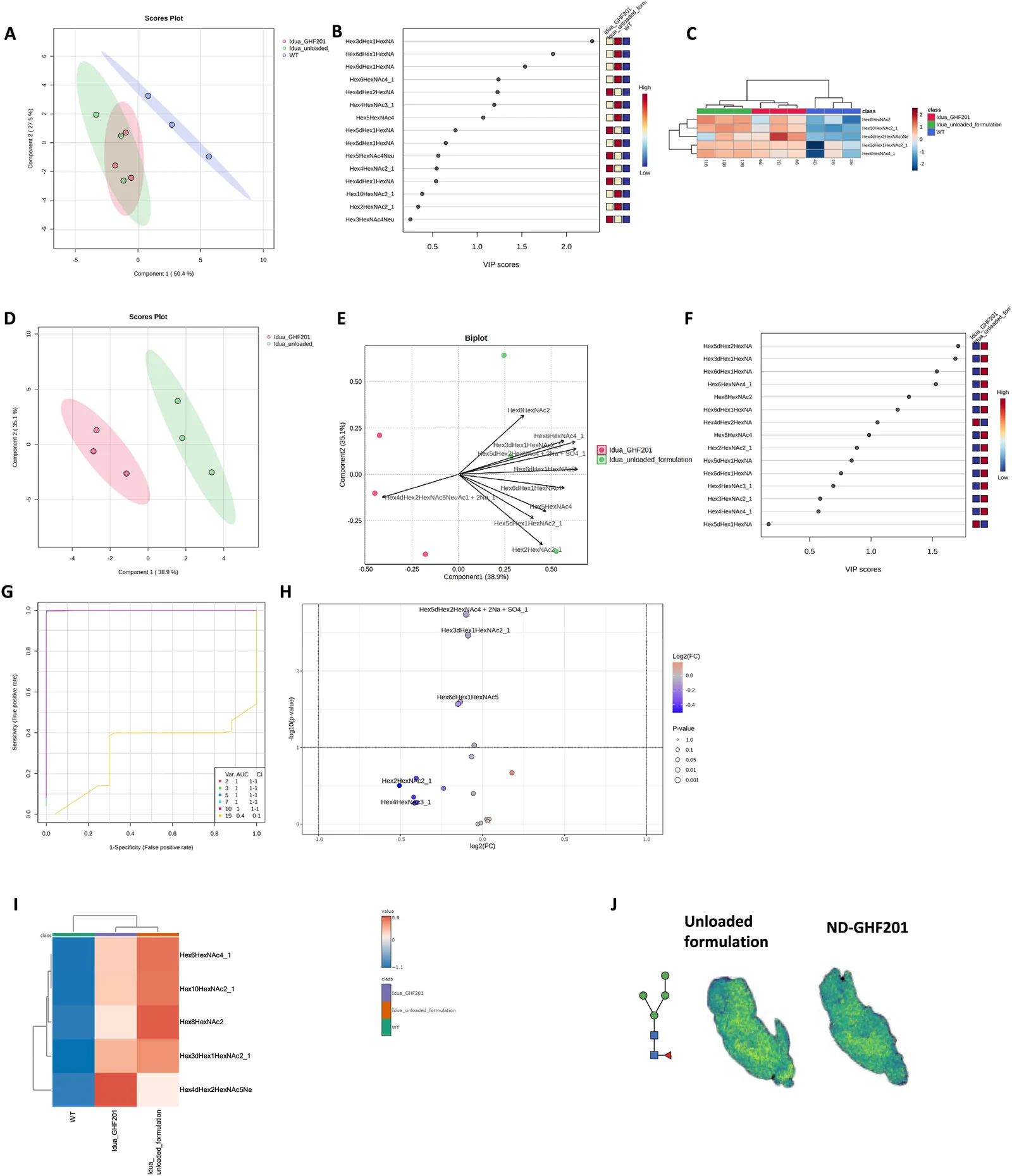
MALDI-IMS profiling of brain N-glycans. **(A)** Supervised PLS-DA dimension reduction analysis of 20 prefiltered brain N-glycans in *Idua*^*+/+*^, *Idua*^*−/−*^, and GHF201-treated *Idua*^*−/−*^ mice (n = 3 mice per group) showing that when all three groups are analyzed together, *Idua*^*−/−*^ mice (treated and untreated) are distinguished from *Idua*^*+/+*^ mice. **(B)** VIP ranking of the relative contribution of the indicated N-glycans to a PLS-DA classification of *Idua*^*+/+*^, *Idua*^*−/−*^, and GHF201-treated *Idua*^*−/−*^ mice. **(C)** Clustering heatmap of 5 N-glycans, which were significantly different among the three groups by one-way ANOVA. **(D)** PLS-DA dimension reduction analysis of brain N-glycans from treated and untreated *Idua*^*−/−*^ mice shows separation of the two groups. **(E)** Biplot visualization showing again that brain N-glycan fingerprints are distinct between treated and untreated *Idua*^*−/−*^ mice (red and green dots) and also, as shown by the loadings (arrows), that untreated *Idua*^*−/−*^ mouse brains manifest accumulated N-glycans (relatively long arrows), which contribute to variability more than the treated mice. **(F)** VIP plot showing the N-glycans contributing to overvariability between treated and untreated *Idua*^*−/−*^ mouse brains. **(G)** ROC curve showing that groups of 2–10 brain N-glycans can classify (AUC = 1) treated and untreated *Idua*^*-/*^ mice. **(H)** Volcano plot of brain N-glycans from *Idua*^*-/*^ mice (treated over untreated) showing that most N-glycans significantly modified by the treatment were reduced. **(I)** Heatmap showing average relative levels of the five brain N-glycans significantly different among *Idua*^*+/+*^, *Idua*^*−/−*^, and GHF201-treated *Idua*^*−/−*^ mice. **(J)** MALDI ion image of the representative N-glycan Hex4dHex1HexNac2_1 in sagittal brain sections from *Idua*^*−/−*^ mice treated with unloaded nonaqueous oral solution (empty-ND) or ND-GHF201, as indicated. Hex4dHex1HexNac2_1 was slightly, but significantly, reduced by GHF201. VIP, Variables Importance in Projection. Source data are available for this figure.

We used the grip-strength, open-field, and MWM tests to test whether GHF201 improved locomotor and memory deficits compared with *Idua*^*+/+*^ littermates. Importantly, sex dimorphism has been observed in MPS-I mice (30), presumably because of variations in epidermal growth factor receptor quantities (31), which are crucial in controlling GAG synthesis rates (32, 33). EGF binding to its receptor is directly proportional to the amount of testosterone (34). Thus, male mice often show a more severe phenotype (35) and a poorer response to therapy (30). This sex dimorphism has not been observed in patients with MPS-I and appears to be unique to the mouse model, similar to models of many other diseases. In view of these known differences, we tested GHF201’s effect on behavioral features separately in male and female mice. 70 mg/Kg ND-GHF201 was administered daily p.o. for 5.5 mo starting at 3 mo of age. In line with the reported sex dimorphism, we found significant improvement or improvement trends in female mice, whereas male mice responded differently: in the grip-strength test, *Idua*^*−/−*^ males did not present with phenotype or response to treatment (Fig 2A). Despite the expected aggravation in grip-strength phenotype in males (because of increased EGF sensitivity), the baseline muscle mass reserve characteristic of male rodents might have transiently masked the MPS-I musculoskeletal pathology during early- to mid-disease stages. Conversely, GHF201 treatment caused a trend of improvement in grip strength in females (*P* < 0.07). In the open-field test (Fig 2B), *Idua*^*−/−*^males did present with a compromised phenotype but, as opposed to the females (whose open-field movement was significantly improved by GHF201, *P* < 0.0054), did not respond to GHF201 treatment. Only in the cognitive tests, open-field habituation (Fig 2C), and MWM (Fig 2D), did male *Idua*^*−/−*^mice show similar trends to those observed in females, that is, compromised phenotype in the transgenic mice and partial correction by GHF201. Given the absence of a baseline phenotype in the grip-strength assay and the attenuated therapeutic response observed in the open-field test among male mice, all subsequent functional and molecular analyses (Figs 3, 4, 5, 6, 7, and 8) focused exclusively on the female cohort.

In female *Idua*^*−/−*^ mice, GHF201 treatment ameliorated muscle dysfunction in the grip-strength assay (Fig 2A) and the mobility (distance traveled) in the open-field test compared with untreated females (Fig 2B). Improved locomotion is also illustrated in our sample video (Video 1 and Video 2). In addition, Video 3 and Video 4 demonstrate that GHF201 treatment led to the regaining of tail up-lifting reflex lost in *Idua*^*−/−*^ mice. Cognitive function in the open field was assessed by comparing habituation time, which evaluates long-term, nonassociative, spatial memory (36, 37). In this test, a mouse is placed in an open field over two (or three) consecutive days and is allowed to explore for 5 min. The longer the animal spends exploring its environment after the first day, the more compromised its nonassociative memory is considered. The nonassociative spatial memory was compromised in both *Idua*^*−/−*^ males and females (Fig 2C). It was corrected robustly by GHF201 in females (*P* < 0.002) and to a smaller extent (*P* < 0.065) in males. In addition, we assessed spatial learning by testing mice daily over five consecutive days in the MWM test (38). GHF201 treatment improved the spatial learning deficit in both male and female *Idua*^*−/−*^ mice. After therapy, treated mice of both sexes exhibited escape latencies that dropped progressively over the 5-d trial, ultimately reaching an efficacy ceiling that was statistically indistinguishable from healthy *Idua*^*+/+*^ wild-type controls (Fig 2D).

Video 1 Locomotion, untreated *Idua*^−*/*−^ mouse. Download video

Video 2 Locomotion, treated *Idua*^−*/*−^ mouse. Download video

Video 3 Tail uprighting, untreated *Idua^−^*^*/*−^ mice. Download video

Video 4 Tail uprighting, treated *Idua*^−*/*−^ mice. Download video

### IVIS and on-gel studies demonstrate attenuation of tissue cathepsins by GHF201

After the different treatments, mice were injected i.v. with the fluorescent cathepsin activity probe GB123 described above for imaging of liver and knee joints, as representative tissues affected strongly in MPS-I, by IVIS. Importantly, in contrast to MPS-I–isolated cells (Fig 1B), where a beneficial treatment effect is manifested by an increase in lysosomal cathepsin activity reflecting improved lysosomal function, in the original biological environment, especially in interstitial tissues of MPS-I models and patients, an increase in cathepsin levels is considered pathological. The concentration of these enzymes increases extracellularly because of their stabilization by the excessive unprocessed GAGs in the ECM causing indiscriminate damage (7, 9, 39). Therefore, somewhat unintuitively, although GB123 can detect both extracellular and intracellular cathepsin activity, the increased GB123 fluorescence in this experiment (*P* < 0.0042 in liver; *P* < 0.0037 in knee joints) suggests pathological, high extracellular cathepsin activity, and lower activity upon treatment with GHF201 is the desired outcome. Indeed, GHF201 lowered overall cathepsin levels in liver (trend with *P* < 0.067) and knee joints (*P* < 0.0047) of *Idua*^*−/−*^ mice, bringing them closer to those of *Idua*^*+/+*^ mice (Fig 3A), presumably thanks to reduced accumulation of extracellular GAGs. Interestingly, our on-gel GB123-based cathepsin activity assay shows that also in resected liver lysates, ND-GHF201 treatment slightly lowered total cathepsin activity whether the activity probe GB123 was injected in vivo, or added ex vivo to liver extracts (Fig 3B). The on-gel results also reveal that GB123-bound cathepsins accumulate in *Idua*^*−/−*^ mouse liver, with a small, but detectable, reduced mobility shift especially of cathepsin B, possibly related to hyperglycosylation (40) as a result of secondary lysosomal dysfunction impairing glycan trimming by lysosomal glycosidases. This cathepsin B hyperglycosylation has also been in other lysosomal storage disorders such as mannosidosis (41) and Niemann–Pick disease types C1 and C2 (42), and is reduced by GHF201 as demonstrated in the lower panel in Fig 3B, where presumably glycosylated active cathepsin B species are shown. Cathepsin accumulation in *Idua*^*−/−*^ mouse livers is probably related to the high GAG levels in this strain, which generate steric hindrance (43), inhibiting cathepsin degradation by lysosomal hydrolases. In addition, the increased cathepsin levels can be caused by compensatory overexpression, as observed for cathepsin B in *Idua*^*−/−*^ mice (10).

### Deficient systemic biomarkers ameliorated by GHF201

Neurofilament light chain (LC-NfL) is a plasma biomarker of neurodegeneration that strongly correlates with cerebrospinal fluid concentrations (44, 45, 46). In both MPS II mouse models (47) and MPS II patients (48), reductions in LC-NfL correlate with therapeutic phenotypic improvements. To evaluate whether LC-NfL is responsive to GHF201, we measured plasma levels using a single-molecule array (Fig S12A). We found a fourfold increase in LC-NfL in *Idua*^*−/−*^ mice, which was reduced by 30% by GHF201.

To dissect metabolic pathway enrichments associated with GHF201 treatment, we conducted an untargeted metabolomics analysis (Fig 4A–G). Partial least-squares discriminant analysis (PLS-DA) showed that the plasma metabolome of GHF201-treated and untreated *Idua*^*−/−*^ mice was distinct from each other and from *Idua*^*+/+*^ mouse plasma metabolome (Fig 4A). The ROC curve also shows that treated and untreated plasmas are separated with two groups of 15 and 10 specific metabolites having a discriminatory power which generates an AUC of 0.94 (Fig 4B). Clustering heatmap analysis (Fig 4C) showed an increase in catabolic metabolites, such as pyruvate and citrate, which is consistent with a GHF201-prompted increase of systemic catabolic activity, similar to the effects of the compound in models of glycogen storage disorder types III (49) and IV (27), quantified by indirect calorimetry (metabolic cages). Interestingly, metabolite set enrichment analyses against two metabolite databases, KEGG (Fig 4D) and SMPDB (Fig 4E), similarly showed that GHF201 enriched catabolic metabolite sets, including the pyruvate-generating pyruvaldehyde degradation and TCA. In addition, the analysis using both these databases revealed that GHF201 induced a significant increase in amino sugar metabolism, which includes intralysosomal GAG degradation, possibly compensating for the lysosomal GAG accumulation in MPS-I. Prediction of enriched metabolic pathways (Fig 4F and G) showed that histidine and alanine metabolism were co-enriched (SMPDB). Enrichment of histidine metabolism is expected to produce glutamate, which can feed into the TCA cycle by its conversion to α-ketoglutarate. Enrichment of alanine metabolism and co-enrichment of alanine and pyruvic acid are probably related to both gluconeogenesis in the liver and glycolysis in the muscle (the glucose–alanine cycle) implicating high carbohydrate turnover. Pathway enrichment analysis against the KEGG database showed enrichment of alanine, aspartate, and glutamate metabolism, as well as TCA cycle. Together, enrichments across histidine, alanine, aspartate/glutamate, and the TCA cycle implicate that GHF201 treatment led to catabolic activation. These enrichments are also consistent with activation of anaplerosis: the active scavenging of the amino acid pool to compensate for an increased demand for TCA cycle intermediates.

Lastly, we determined the effect of GHF201 on GAG levels in urine. Accumulation of GAGs in urine is one of the key hallmarks of MPS-I. Therefore, it was important to test the effect of GHF201 on this biomarker. As can be seen in Fig S12B, GHF201 significantly reduced GAG levels (measured, as previously by the Blyscan kit) in urine, which accumulated in *Idua*^*−/−*^ as compared to *Idua*^*+/+*^ mice.

### Deficient organ-specific biomarkers ameliorated by GHF201

To test whether GHF201 affects bone dysplasia, commonly observed in MPS-I, we imaged the hindlimbs using CT. *Idua*^*−/−*^ mice showed substantial bone dysplasia, especially in the knee joint region, in agreement with previously reported skeletal abnormalities in this model and in patients with MPS-I (50) (Fig 5A). We quantified the dysplasia by the ratio of tibia shaft length to joint width, as described previously (50, 51). GHF201 treatment increased this ratio by 20%, normalizing it to levels similar to those observed in *Idua*^*+/+*^ mice.

Spleen pathology in MPS-I is manifested as splenomegaly (52, 53). We therefore weighed the spleens of GHF201-treated and untreated *Idua*^*−/−*^ mice and compared it with the spleen weight of *Idua*^*+/+*^ mice. Our results (Fig 5B) show a significant (*P* < 0.0001) 50% increase in spleen weight in *Idua*^*−/−*^ mice, which was reduced significantly (by 15%, *P* < 0.001) in GHF201-treated mice.

Liver histopathology was assessed by transmission electron microscopy (TEM, Fig 5C). Multiple large vacuoles were observed in both hepatocytes, identified by cytoplasmic glycogen rosettes, and resident macrophages (mostly Kupffer cells lacking glycogen rosettes). GHF201 treatment reduced the size and amount of these vacuoles in both cell types suggesting improvement of lysosomal function and offset of lysosomal swelling. In line with this observation, liver autophagy was also activated by GHF201 treatment as demonstrated by decreases in p62, and LC3II/LC3I, phosphor-S6/S6, phosphor-mTOR/mTOR, and phosphor-Akt/Akt ratios (Fig 5D). These decreases were observed in liver lysates subjected to SDS–PAGE and subsequent Western immunoblotting.

### GHF201 reverses neuroinflammation and Purkinje cell vacuolation

Neuroinflammation is a hallmark phenotype in MPS-I patients and corresponding mouse models (54), characterized by the robust activation of astrocytes and microglia accompanied by the secretion of pro-inflammatory cytokines (55, 56). To evaluate whether GHF201 could mitigate this neuroinflammatory cascade, we immunostained the frontal cortex of treated and untreated *Idua*^*−/−*^ mice for GFAP (astrocytes) and Iba1 (activated microglia), comparing them with *Idua*^*+/+*^ wild-type baselines (Fig 6A). Both markers were substantially elevated in the untreated KO mice and significantly reduced after systemic GHF201 treatment. Concurrently, we assessed the therapeutic impact on the cerebellum by scoring the extent of vacuolation in Purkinje neurons, which typically swell with large cytoplasmic vacuoles in MPS-I because of impaired autophagic flux (57). GHF201 treatment significantly ameliorated Purkinje cell vacuolation (Fig 6B). This structural rescue correlated directly with a restoration of autophagy, as evidenced by the clearing of accumulated LC3 puncta and the autophagic substrate p62 (Fig 6C). Together, these findings demonstrate that systemically administered GHF201 successfully crosses the blood–brain barrier to suppress chronic neuroinflammation and attenuate neuronal autophagic defects.

### Pharmacological induction and suppression of autophagy respectively mimic and counter some of GHF201 effects

The working assumption of this study is that the beneficial effects of GHF201 in the MPS-I mouse model are associated with enhancement of lysosomal and autophagic functions. Therefore, alongside GHF201, we evaluated the autophagy inducer rapamycin as a positive control and the autophagy inhibitor 3-methyladenine (3-MA) as a negative control. Both compounds were administered as stand-alone treatments in *Idua*^*−/−*^ mice not exposed to GHF201. For direct visual comparison, the corresponding wild-type, untreated *Idua*^*−/−*^, and GHF201 datasets from Figs 2, 3, 5, and 6 are replotted alongside these cohorts as reference comparators. Rapamycin (10 mg/kg) and 3-MA (15 mg/kg) were administered every other day for the last two weeks before euthanasia to minimize the known toxic effects of these drugs. We believe that these putative toxic effects might confound the effects of rapamycin and 3-MA as respective inducer and inhibitor of autophagy and thus GHF201 comparators. This might explain why rapamycin not always produced an effect similar to the ameliorating effect of GHF201, whereas 3-MA did not always produce the opposite effect. In addition, rapamycin and 3-MA often produced effects similar or opposite to GHF201, respectively, but only as trends which were not statistically significant.

**Figure S2.**
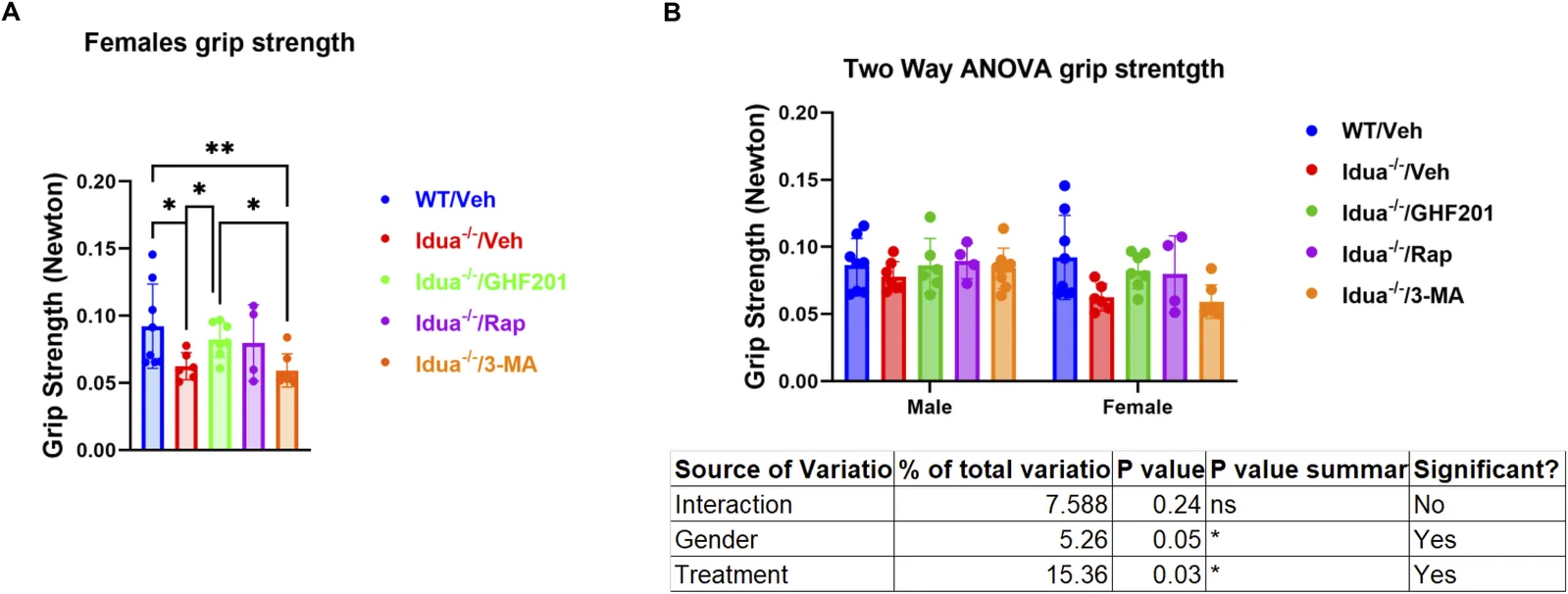
GHF201 ameliorates grip strength in *Idua*^−*/*−^ female mice. **(A)** Grip strength in female mice was measured in n = 6 WT mice, or n = 6 *Idua*^*−/−*^ mice treated with unloaded ND vehicle (WT/Veh and Idua−/−, respectively); n = 6 *Idua*^*−/−*^mice treated with a ND excipient loaded with GHF201 (*Idua*^*−/−*^/GHF201), n = 4 *Idua*^*−/−*^mice treated with the autophagy inducer rapamycin (*Idua*^*−/−*^/Rap), and n = 4 *Idua*^*−/−*^mice treated with the autophagy inhibitor 3-MA (*Idua*^*−/−*^/3-MA). One-way ANOVA with correction for multiple comparisons shows that GHF201 corrected the decrease in grip strength observed in *Idua*^*−/−*^mice, as compared to WT, whereas the effects of rapamycin and 3-MA respectively increased and decreased grip strength in *Idua*^*−/−*^mice, but not at a statistically significant level. *P < 0.05, **P < 0.01. **(B)** Two-way ANOVA results of grip strength in males and females, showing that as opposed to male mice, grip strength in female mice was significantly affected by GHF201. Details are shown in text. WT, wild type.

In *Idua*^*−/−*^ females, grip strength was compromised and significantly increased by GHF201 (Fig 2), whereas rapamycin increased grip strength, but not at a statistically significant level. Although the grip strength of 3-MA–treated *Idua*^*−/−*^ female mice was markedly lower than that of healthy *Idua*^*+/+*^ controls, 3-MA administration exerted a small but not statistically significant reduction when compared directly to vehicle-treated *Idua*^*−/−*^ females (*P* < 0.31, one-way ANOVA with correction for multiple comparisons). All these effects were observed in females (Fig S2A), similar to the female-selective effect of GHF201 in adult polyglucosan body disease–modeling mice (27). As opposed to *Idua*^*−/−*^ females, *Idua*^*−/−*^ males did not present with a grip deficiency phenotype or grip response to GHF201 or any other treatment (Fig S2B). Two-way ANOVA using gender and treatment as factors indeed shows that both gender (*P* < 0.05) and treatment (*P* < 0.03) significantly contribute to overall variability, confirming the differential effect in females as compared to males. However, the null hypothesis that the treatment differentially affects males and females, or that there is a significant interaction between the factors could not be rejected (*P* < 0.24), possibly because of the relatively small sample sizes and the robust statistical threshold required to demonstrate interaction between factors.

**Figure S3.**
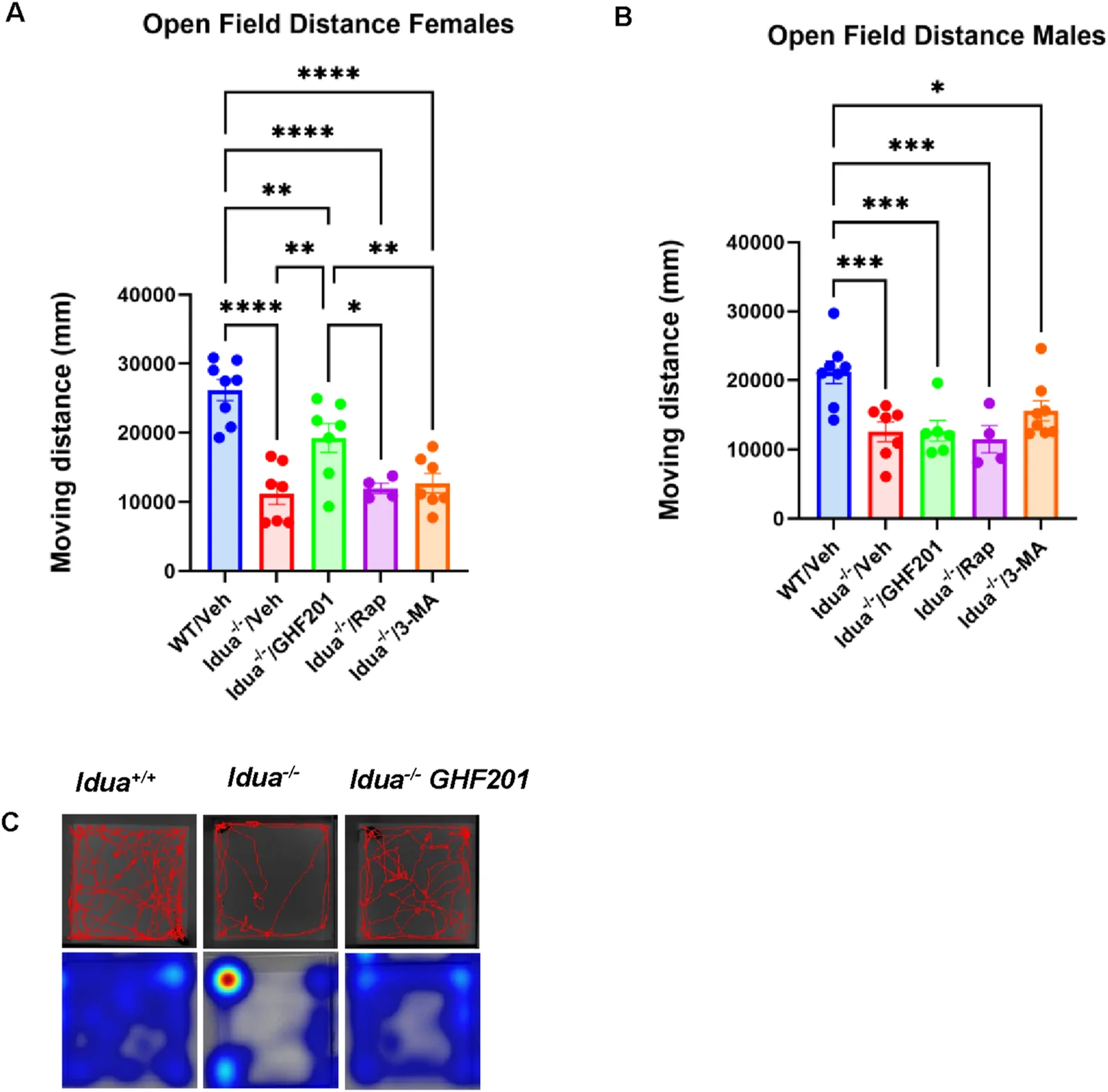
GHF201 ameliorates open field locomotion in *Idua*^−*/*−^ female mice. **(A)** Open-field moving distance in female mice. Open field was measured in n = 8 WT mice or n = 7 *Idua*^*−/−*^ mice treated with unloaded ND vehicle (WT/Veh and *Idua*^*−/−*^, respectively); n = 7 *Idua*^*−/−*^ mice treated with a ND excipient loaded with GHF201 (*Idua*^*−/−*^/GHF201), n = 4 *Idua*^*−/−*^ mice treated with the autophagy inducer rapamycin (*Idua*^*−/−*^/Rap), and n = 7 4 *Idua*^*−/−*^ mice treated with the autophagy inhibitor 3-MA (*Idua*^*−/−*^/3-MA). One-way ANOVA with correction for multiple comparisons shows that GHF201 corrected the decrease in open-field locomotion observed in *Idua*^*−/−*^ mice, as compared to WT, whereas rapamycin and 3-MA did not affect open-field locomotion. *P < 0.05, **P < 0.01, ***P < 0.001, ****P < 0.0001. **(B)** Open-field moving distance in male mice. See (A) for group details. **(C)** Examples of single animal tracking and sojourn heatmaps of (left to right) *Idua*^*+/+*^ female, *Idua*^*−/−*^ female, and GHF201-treated *Idua*^*−/−*^ female. See Fig 2B. WT, wild type.

Gross motor activity, as evaluated by total moving distance in the open-field test, is intrinsically dependent on peripheral musculoskeletal force and neuromuscular grip capacity. Motor activity, measured by the open-field test, is influenced by muscle or grip strength and was therefore reduced in *Idua*^*−/−*^ mice (Fig 2) and expected to be improved by rapamycin and inhibited by 3-MA. However, although the compromised open-field locomotion in *Idua*^*−/−*^ mice was improved by GHF201 (Fig 2), it was not significantly influenced by rapamycin and 3-MA (Fig S3A–C).

**Figure S4.**
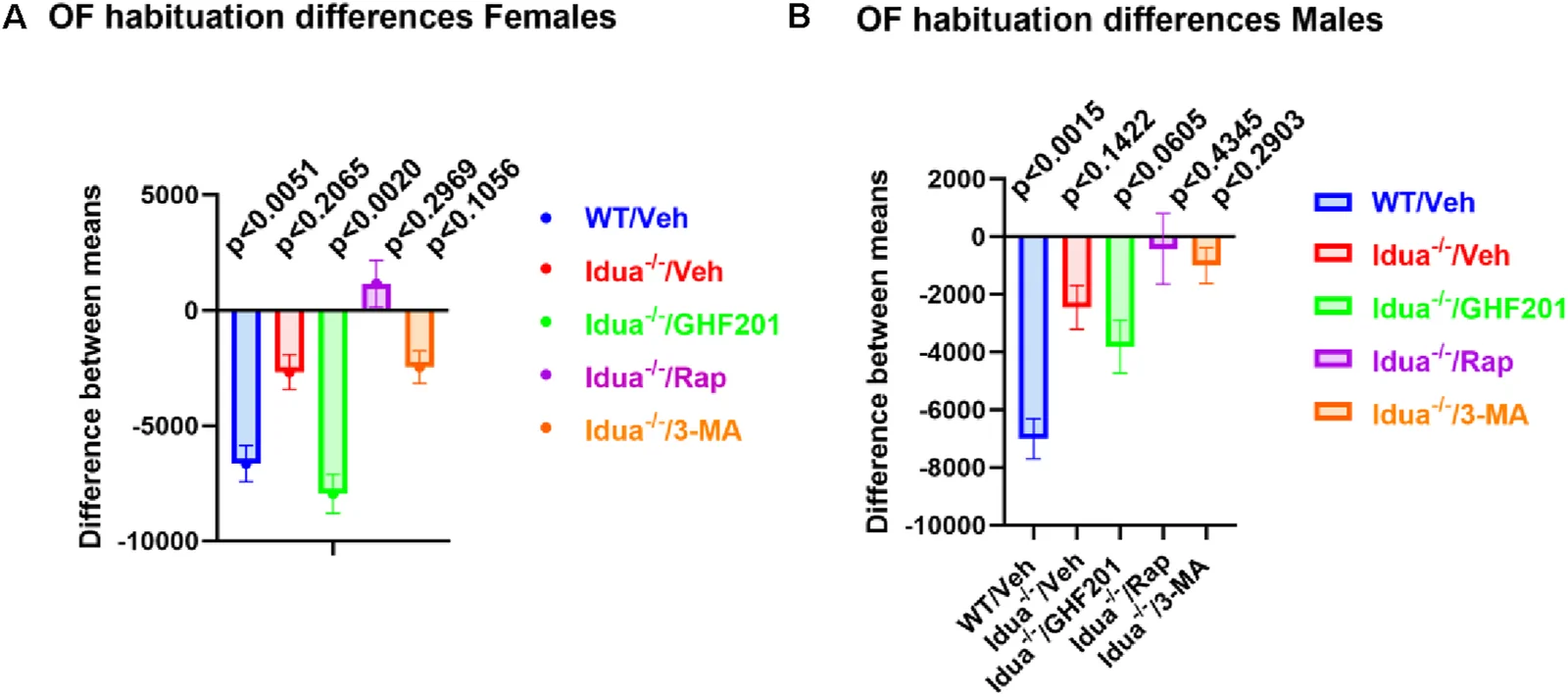
GHF201 ameliorates open field locomotion in *Idua*^−*/*−^ female and male mice. **(A)** OF habituation mean differences and their respective *P*-values are shown for male (A) and female (B) mice. See Fig S2 for group assignments and animal numbers. The conclusion is that OF habituation (nonassociative memory) was improved by GHF-201 in *Idua*^*−/−*^ females more than in *Idua*^*−/−*^ males. OF, open field.

In addition to its effect on motor capacities, we tested the effect of rapamycin and 3-MA, in comparison with GHF201, on cognitive capacities (Fig S4A and B). Nonassociative spatial memory, evaluated by open-field habituation (Fig 2C), was compromised in *Idua*^*−/−*^ mice and corrected by GHF201 in both female and male mice with a stronger effect in female mice. However, rapamycin and 3-MA did not have a significant effect on nonassociative spatial memory in *Idua*^*−/−*^ mice.

**Figure S5.**
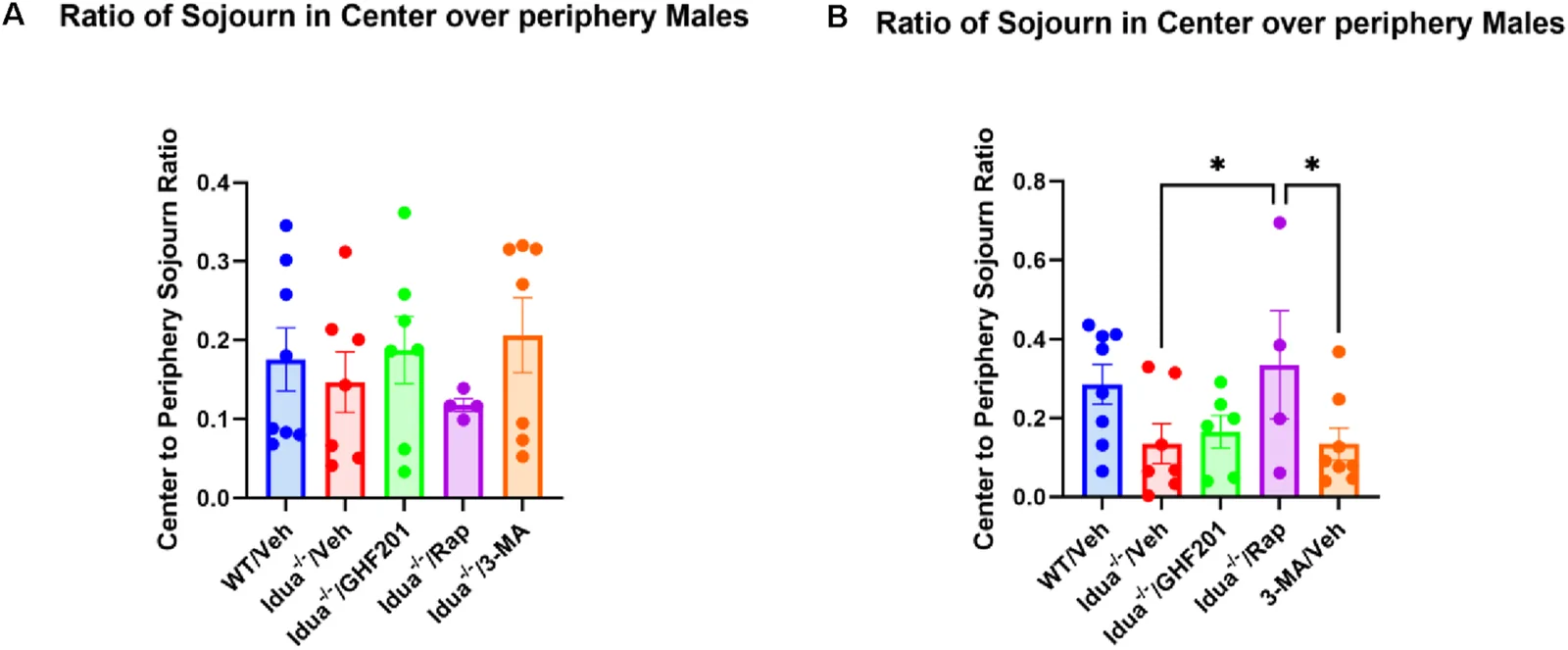
GHF201 does not significantly affect anxiety in *Idua*^−*/*−^ female and male mice. **(A)** Relative sojourn in the center of an open field, as an inverse estimate of anxiety in female mice. Relative sojourn in the center of an open field was measured in n = 8 WT mice, or n = 7 *Idua*^*−/−*^ mice treated with unloaded ND vehicle (WT/Veh and *Idua*^*−/−*^, respectively); n = 7 *Idua*^*−/−*^ mice treated with a ND excipient loaded with GHF201 (*Idua*^*−/−*^/GHF201), n = 4 *Idua*^*−/−*^ mice treated with the autophagy inducer rapamycin (*Idua*^*−/−*^/Rap), and n = 7 4 *Idua*^*−/−*^ mice treated with the autophagy inhibitor 3-MA (*Idua*^*−/−*^/3-MA). One-way ANOVA with correction for multiple comparisons shows no significant anxiety or correction thereof. **(B)** Same results in male mice, except n = 6 for *Idua*^*−/−*^/GHF201 mice, and n = 8 for *Idua*^*−/−*^/3-MA mice. *P < 0.05. The conclusion is that no apparent anxiety or correction thereof was detected by this test in females. Anxiety is detected in males and can be corrected by rapamycin, but not by GHF201. WT, wild type.

Anxiety (Fig S5) was assessed by the ratio between sojourn at the center and periphery in an open field, based on the premise that reduced time at the center demonstrates risk avoidance and anxiety. According to our results (Fig S5), no anxiety and/or correction thereof was observed in *Idua*^*−/−*^ female mice. This suggests that the baseline locomotor deficits reflect a physical motor limitation rather than an altered central exploratory drive or emotional baseline. In *Idua*^*−/−*^ males, anxiety was improved by rapamycin, but not by GHF201, and 3-MA increased anxiety in rapamycin-treated animals.

**Figure S6.**
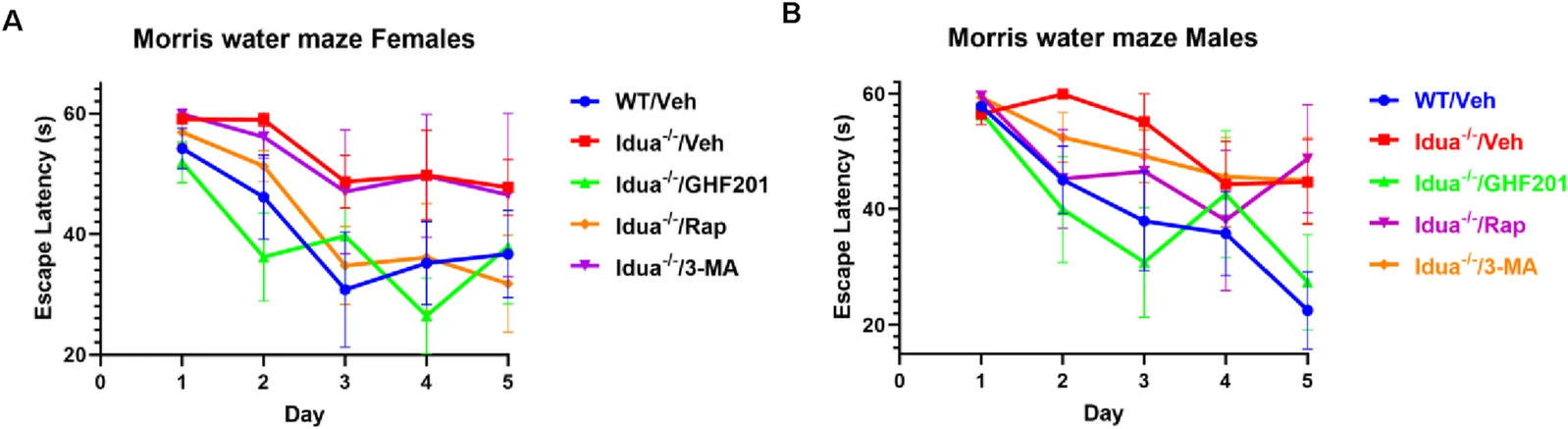
GHF201 and rapamycin improved, while 3-MA exacerbated performance in Morris Water Maze test in *Idua*^−^*^/^*^−^ female mice. **(A)** Change over time in latency to escape to a submerged platform as an inverse estimate of spatial learning in female mice. This escape latency was measured over 5 d in n = 8 WT mice, or n = 7 *Idua*^*−/−*^ mice treated with unloaded ND vehicle (WT/Veh and *Idua*^*−/−*^, respectively); n = 7 *Idua*^*−/−*^ mice treated with a ND excipient loaded with GHF201 (*Idua*^*−/−*^/GHF201), n = 4 *Idua*^*−/−*^ mice treated with the autophagy inducer rapamycin (*Idua*^*−/−*^/Rap), and n = 7 *Idua*^*−/−*^ mice treated with the autophagy inhibitor 3-MA (*Idua*^*−/−*^/3-MA). One-way ANOVA with repeated measures shows that reduction in escape latency over time, or spatial learning, is reduced in *Idua*^*−/−*^ mice as compared to WT (*P* < 0.03) and improved by GHF201 (*P* < 0.02), such that the difference in spatial learning between *Idua*^*−/−*^ and WT mice became insignificant (*P* < 0.50). In addition, rapamycin increased (*P* < 0.05) and 3-MA decreased (*P* < 0.05) spatial learning in *Idua*^*−/−*^ mice. **(B)** Spatial learning in male mice was analyzed as in (A). In *Idua*^*−/−*^ mice, spatial learning was lower than in WT (*P* < 0.03). GHF201 treatment increased spatial learning in *Idua*^*−/−*^ mice (*P* < 0.05), rendering it indistinguishable from WT (*P* < 0.48). Rapamycin and 3-MA did not exert a significant effect on spatial learning in *Idua*^*−/−*^ mice (*P* < 0.15 and *P* < 0.17, respectively). WT, wild type.

Spatial learning was assessed by the MWM (Fig 2D). Our results (Fig S6A and B) demonstrate that rapamycin and 3-MA respectively increased and decreased spatial learning, compromised in the transgenic strain, in females only (Fig S6A). This is in contrast to GHF201 treatment, which repaired spatial learning, rendering it indistinguishable from values obtained in the *Idua*^*+/+*^ mice, in both female and male Idua^−/−^ mice.

**Figure S7.**
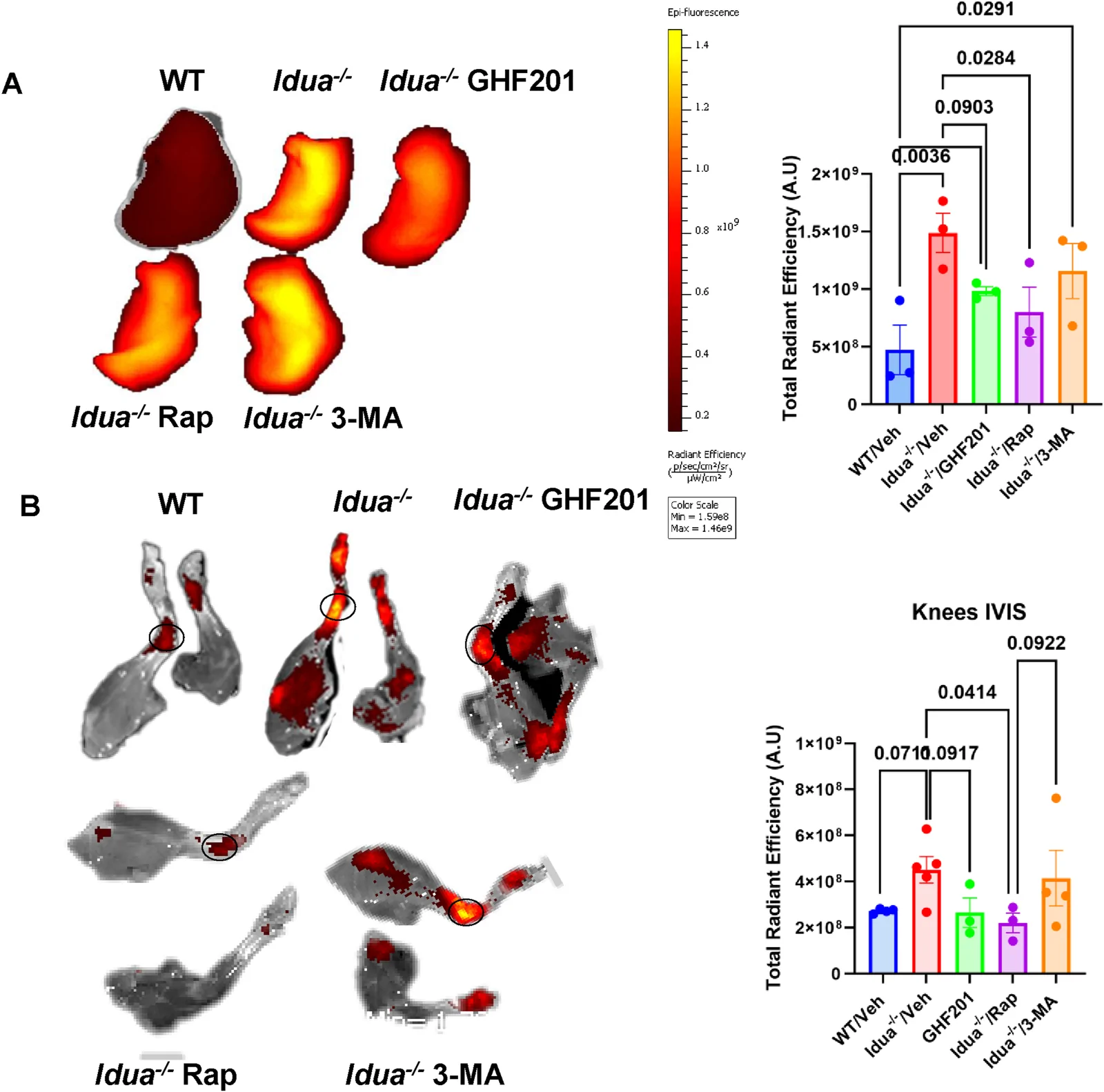
Reduction in cathepsin activity in situ. Left panels, total radiant fluorescence efficiency of GB123 in liver (A) and knee joints (B) of female *Idua*^*−/−*^ mice treated for 5.5 mo with GHF201. Right panels, total radiant fluorescence efficiency was quantified in 3–4 mice per group, presented as the mean ± SD. *P*-values were calculated using a one-way ANOVA with correction for multiple comparisons.

**Figure S8.**
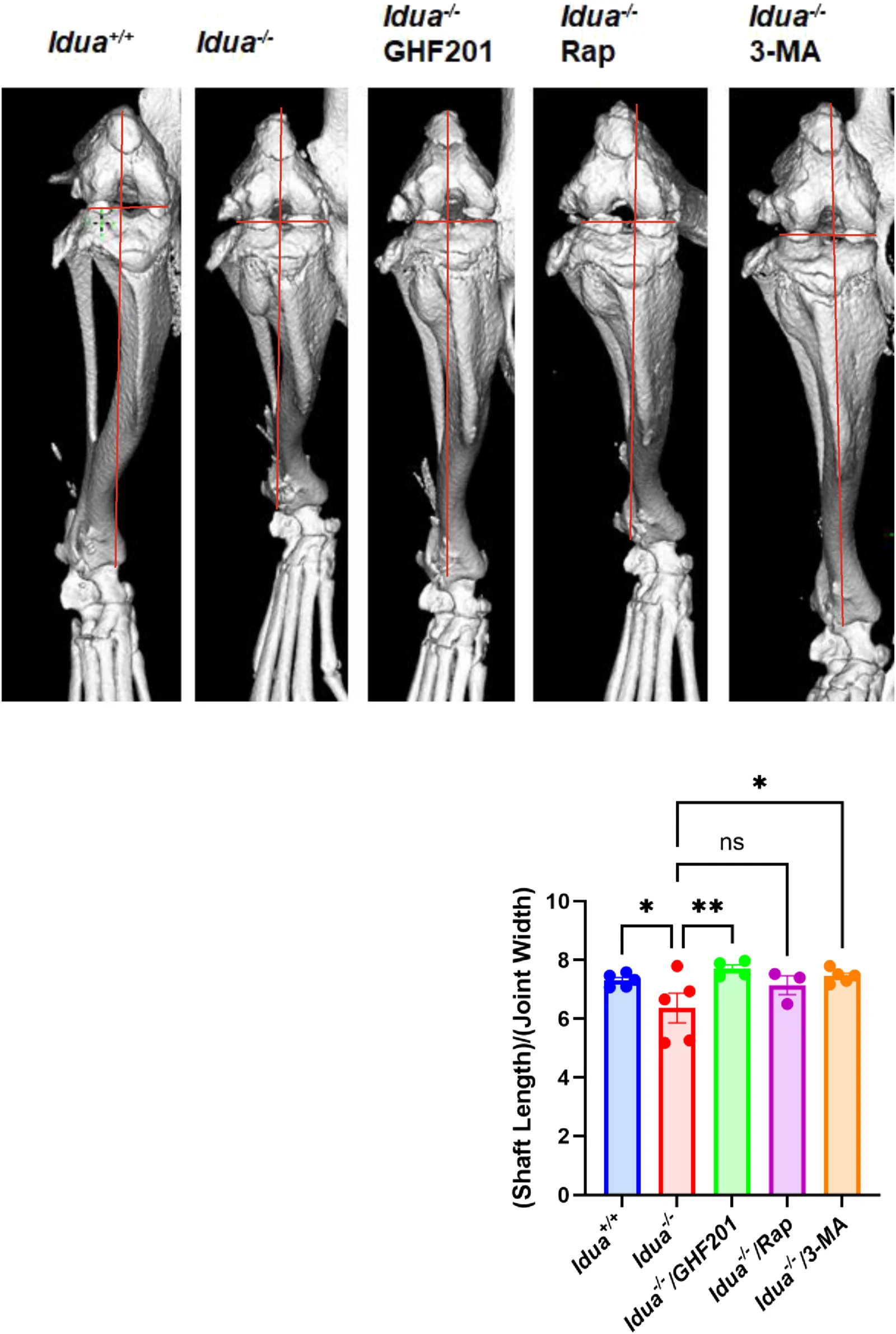
GHF201 corrects knee joint bone dysplasia. *Upper panel*, representative CT images of legs of *Idua*^+/+^, and vehicle-, GHF201-, rapamycin-, and 3-MA–treated *Idua*^−/−^ mice illustrating bone dysplasia (especially note knee joint deformity and reduced tibia length-to-joint width ratio) in *Idua*^−/−^ mice and its partial correction by GHF201. Shaft length and joint width parameters are demarcated by lines in the representative CT images. *Lower panel*, quantification of (shaft length)/(joint width) as a measure of bone dysplasia. n = 3. *P*-values were determined by one-way ANOVA with correction for multiple comparisons. *, *P* < 0.05; **, *P* < 0.01.

**Figure S9.**
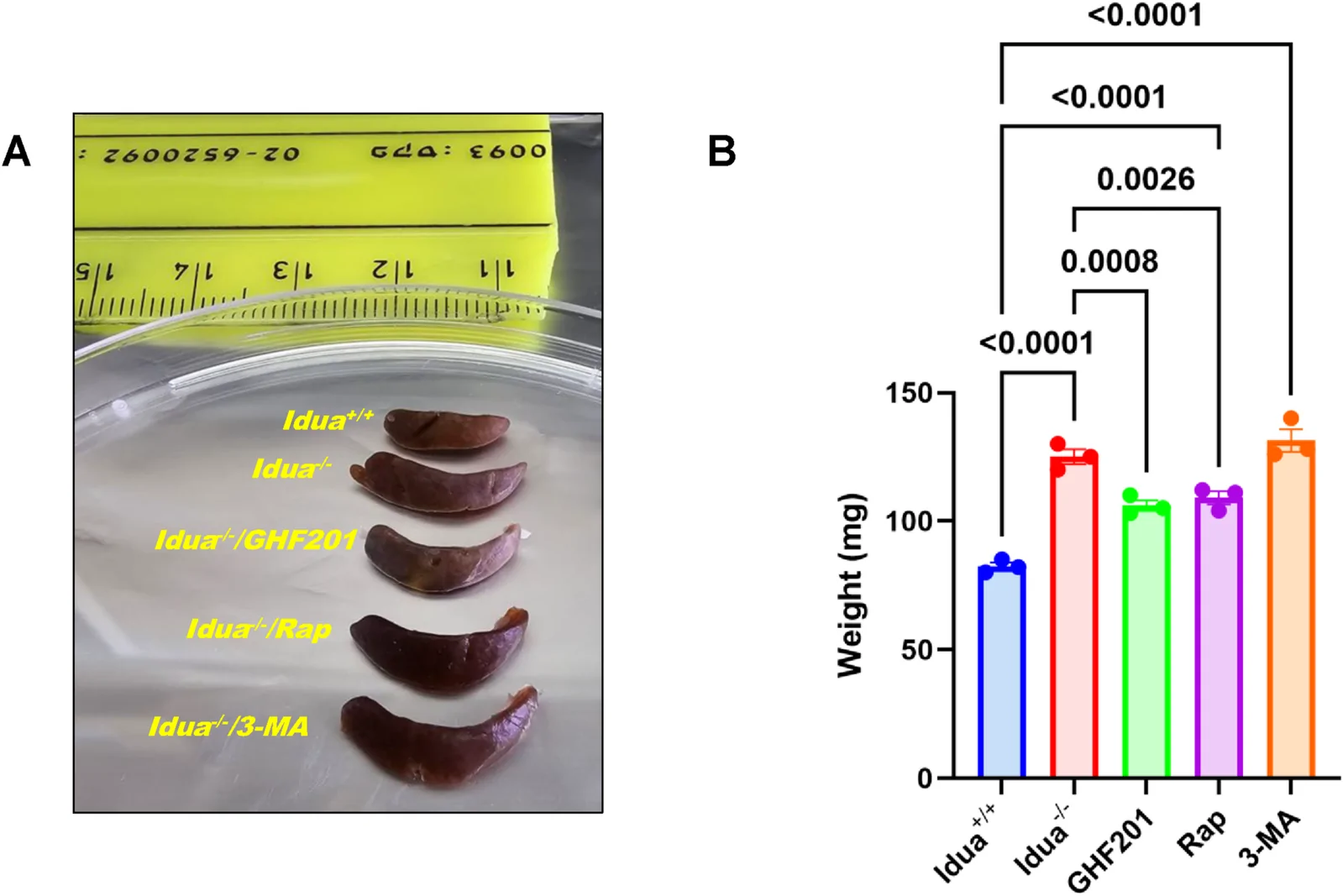
Normalization of splenomegaly. **(A)** Representative picture of the spleens collected from 8-mo-old mice from the indicated genotypes and treatments. **(B)** Mean weights of spleens in the indicated groups. One-way ANOVA with correction for multiple comparisons shows that spleen weights were significantly increased in *Idua*^*−/−*^ mice and decreased by GHF201.

**Figure S10.**
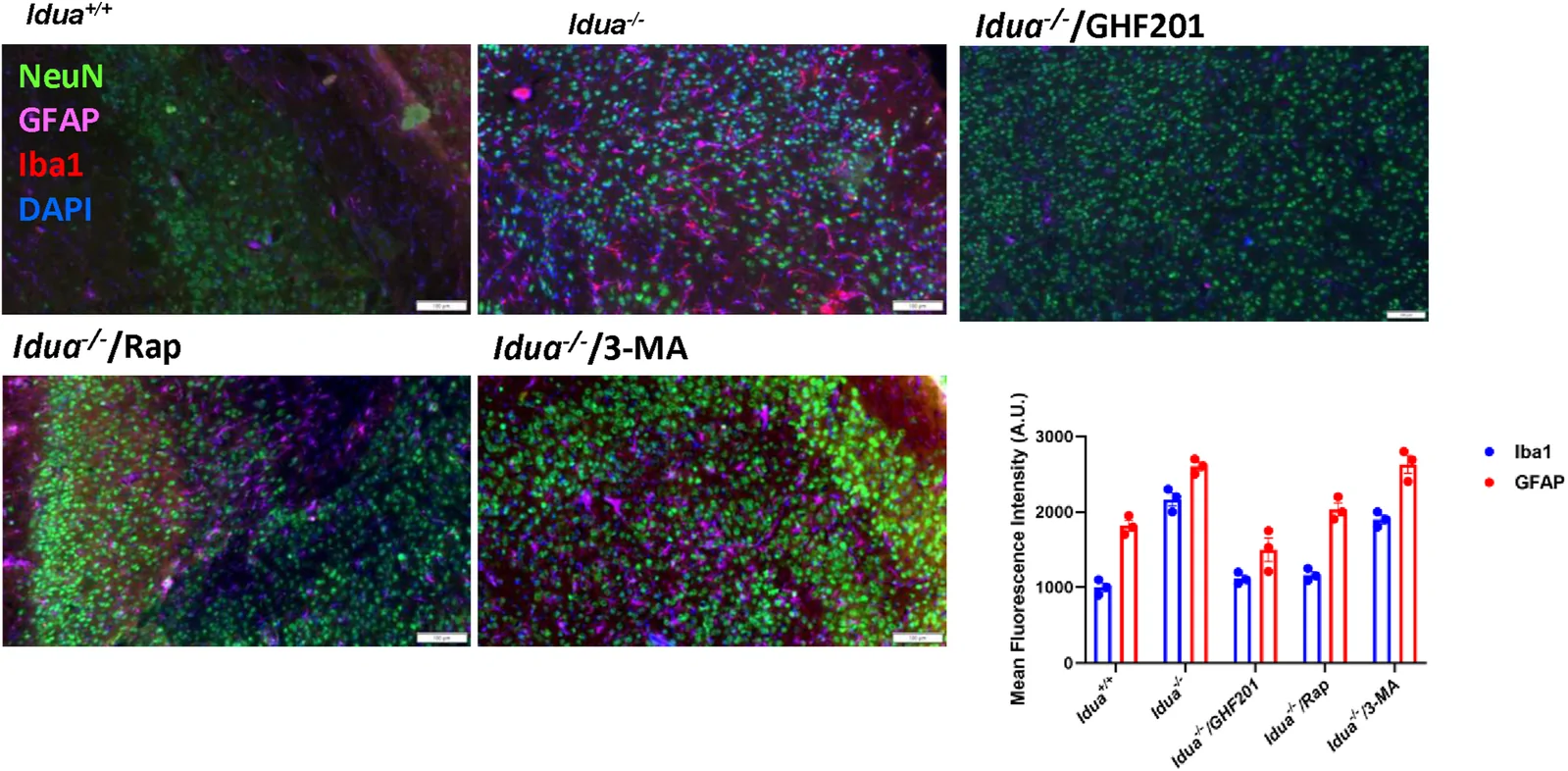
Brain slices from the indicated mouse groups were fixed and stained with NeuN and DAPI to detect neuronal and total cell nuclei, respectively. GFAP and Iba1 antibodies were used as biomarkers of gliosis and microglial activation, respectively. Secondary antibodies were conjugated to FITC, CY3, and CY5 dyes to respectively stain NeuN, Iba1, and GFAP as indicated. Lower panel, quantification of fluorescence intensity of GFAP and Iba1 staining in *Idua*^*+/+*^, *Idua*^*−/−*^, and GHF201-treated *Idua*^*−/−*^ mice (n = 3 per group). Statistical differences were determined by one-way ANOVA with multiple comparisons correction. Scale bars, 100 μm.

**Figure S11.**
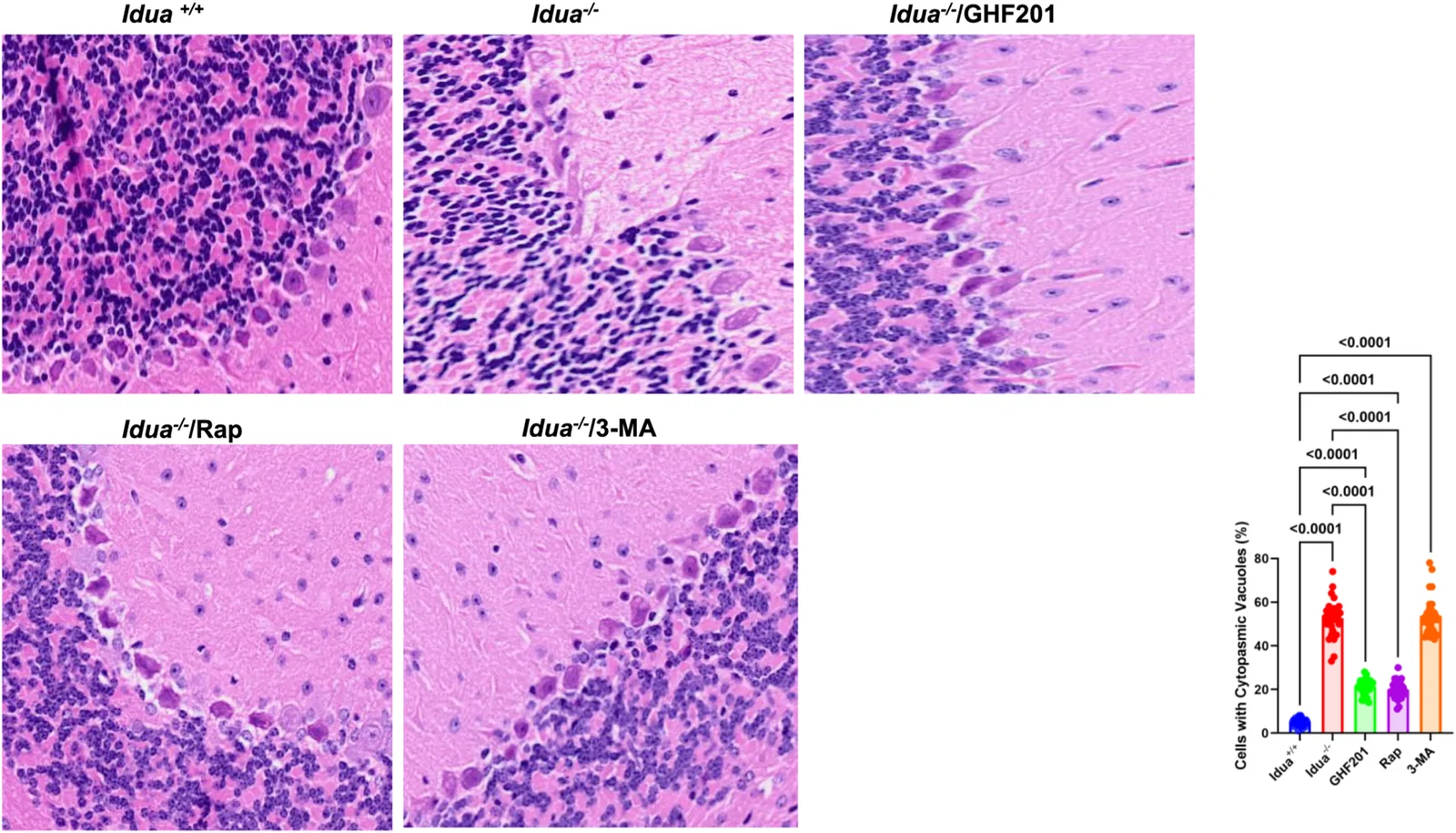
*Left panel*, H&E staining of fixed cerebellum sections taken from mice of the indicated genotypes and treatments. Purkinje cells manifest cytoplasmic vacuoles as a sign of morphological abnormality and stress. Right panel, quantification of the percentage of vacuolated Purkinje cells based on five fields from n = 6 animals per group. One-way ANOVA with correction for multiple comparisons shows that cerebral Purkinje cells are hypervacuolated in *Idua*^*−/−*^ mice. This hypervacuolation is partially restored by GHF201 and rapamycin. Scale bar, 50 μm.

In our postmortem analyses, the effects of the autophagic activator rapamycin followed in general the same direction as those of GHF201. Analysis of GB123 fluorescence by IVIS in the liver and knee joints (Fig S7A and B) revealed that rapamycin treatment elicited an even more pronounced reduction in signal in *Idua*^*−/−*^ mice than GHF201. However, the autophagy inhibitor 3-MA did not increase GB123 IVIS fluorescence beyond the levels observed in untreated *Idua*^*−/−*^ mice. Bone dysplasia (Fig S8) was also ameliorated by rapamycin, but not to a significant level. 3-MA did not exert a significant effect on bone dysplasia in *Idua*^*−/−*^ mice. In countering splenomegaly as well (Fig S9A and B), like GHF201 rapamycin reduced spleen size, whereas 3-MA did not exert a significant effect. Rapamycin and 3-MA were also tested as modulators of neuroinflammation in *Idua*^*−/−*^ mice (Fig S10): like GHF201, rapamycin reduced Iba1 and GFAP cortical levels, whereas 3-MA reduced Iba1 (i. e., mitigated microglial activation), contrary to the effect expected from an autophagy inhibitor, and did not affect GFAP or astrogliosis. Lastly, vacuolation of cerebellar Purkinje neurons (Fig S11) was also mitigated by rapamycin, similar to GHF201, but not affected by 3-MA.

### Ultrastructural resolution of intracranial lysosomal clearance

To resolve these cellular changes at the nanoscale, we conducted ultrastructural examination of the cortex and hippocampus via TEM. Compared with *Idua*^*+/+*^ controls, untreated *Idua*^*−/−*^ mice manifested extensive cytoplasmic vacuolation. Specifically, microglia displayed large, electron-lucent vacuoles—a classic hallmark of water-soluble GAG accumulation, where the water-soluble GAGs wash out during aqueous fixation (58) (Fig 6D). These distended structures were delineated by a defined limiting membrane, identifying them as severely pathological lysosomes. Remarkably, GHF201 treatment significantly reduced the number and volume of these distended lysosomes. This ultrastructural clearance provides direct visual evidence that GHF201 alleviates lysosomal substrate overload and restores functional organelle morphology within the deep tissues of the brain.

### Cell type–specific activation of lysosomal function in vivo

To definitively map the intracranial therapeutic activity of GHF201 across distinct neural lineages, we analyzed freshly resected brain tissue using the cathepsin activity–based probe GB123 combined with multicolor flow cytometry. Brain tissues were homogenized into single-cell suspensions and stained for cell-specific markers: CD11b/c for microglia, nuclear NeuN for neurons, O4 for oligodendrocytes, and ASCA2 for astrocytes, as described in Schroeter et al (59). Consistent with our in vitro embryonic fibroblast data, systemically administered GHF201 significantly up-regulated cathepsin activity directly within isolated microglia, neurons, and oligodendrocytes harvested from adult *Idua*^*−/−*^ brains (Fig 7A and B). Because more than 90% of intracellular cathepsins are localized to the lysosome (60, 61), this targeted increase confirms a direct, cell-autonomous enhancement of lysosomal degradative capacity. Crucially, these cell type–specific profiles demonstrate that GHF201 maintains broad, multilineage therapeutic efficacy throughout the CNS, surmounting a primary bottleneck that limits standard ERTs.

### GHF201 partially reverses the pathological brain N-glycome by attenuating a distinct subset of disease-correlated N-glycans

Matrix-assisted laser desorption/ionization imaging mass spectrometry imaging (MALDI-IMS (62)) analysis after N-glycosidase F (PNGase F) digestion, which cleaves and releases N-glycans from glycoproteins, reports spatial glycoprotein abundance in the brain. Changes in the abundance of these glycoproteins can be the result of changes in glycan biosynthesis and processing and can be influenced by the brain cell composition and by pathology. To phenotype the spatial glycomics signature of *Idua*^*−/−*^ mouse brains and whether it can be modulated by GHF201, formalin-fixed paraffin-embedded specimens were treated with PNGase F followed by the application of ionization matrix and quantitative analysis by MALDI-IMS. Given the small cohort size (n = 3 for each group) and the presence of individual animal outliers, before group-wise comparisons, we filtered high-variance metabolites from the data, removing metabolites with CV<50% across all groups. These data and test statistic–independent filtering (63) ensure that the filtered metabolites are actually changing, have a large enough dynamic range to overcome technical stochasticity, and represent true biological transitions between the experimental groups. Supervised PLS-DA dimension reduction analysis of 20 prefiltered brain N-glycans demonstrated clear separation between *Idua*^*+/+*^ and *Idua*^*−/−*^ mice (both treated and untreated) along the first component accounting for 50.4% of the total variance (Fig 8A). The top-ranking Variables Importance in Projection metabolites contributing to this separation and the direction of the metabolic shifts are shown in Fig 8B. Clustering heatmap of five N-glycans, which were significantly different among the three groups by one-way ANOVA, is shown in Fig 8C. As can be seen, these N-glycans cluster the three groups, and as compared to the *Idua*^*+/+*^ group, both treated and untreated *Idua*^*−/−*^ groups had higher N-glycan levels. In the context of MPS-I, where Idua deficiency leads to the primary accumulation of GAGs in lysosomes with ensuing lysosomal dysfunction, this detected N-glycan increase could be attributed to impairment of endo-lysosomal and autophagic flux, leading to the pathological sequestration of membrane-associated and luminal glycoproteins. We assume that because these molecules cannot be recycled or processed into free sugars, the PNGase F-releasable fraction—which serves as the MALDI-IMS readout—is higher in *Idua*^*−/−*^ than in *Idua*^*+/+*^ mice. Another possibility is that brain glycoprotein accumulation in *Idua*^*−/−*^ mice is a consequence of the chronic neuroinflammation we observed in *Idua*^*−/−*^ mice (Figs 6A and 7), which significantly alters the cellular landscape of the brain. Reactive astrogliosis and microglial activation (Fig 7A and B) involve the robust up-regulation of specific glycosylated markers and various cell-surface receptors (64). Thus, the increased glycomics signal we observed could also represent a spatial signature of this inflammatory response.

When N-glycans from treated and untreated *Idua*^*−/−*^ mice were analyzed separately, the two groups were well separated along the first component (Fig 8D, 38.9% of total variance) with all N-glycans similarly contributing to overall variance, as shown by the vector sizes in the biplot (Fig 8E) and also in the Variables Importance in Projection plot (Fig 8F). Separation between brain glycans of GHF201-treated and untreated *Idua*^*−/−*^ mice is also demonstrated by the ROC curve with groups of 2, 3, 5, 7, and 10 specific metabolites discriminating the two groups with an AUC of 1 (Fig 8G). The volcano plot in Fig 8H shows that the GHF201 treatment mostly led to a decrease in the level of several different N-glycans. Because GHF201 is an autolysosomal enhancer, this observation aligns with a GHF201-dependent activation of lysosomal glycosidases, which would lower the level of glycoprotein substrates and thus the MALDI signal mentioned. The clustering heatmap (Fig 8I) shows that the five most discriminatory N-glycans (*t* tests) clustered all treated and untreated *Idua*^*−/−*^ mice to their respective groups, and in this pairwise comparison, GHF201 significantly decreased brain N-glycans. Lastly, Fig 8J shows a MALDI ion image of the representative N-glycan species Hex4dHex1HexNAc2_1 in a sagittal brain section. This N-glycan was significantly reduced by GHF201 treatment.

## Discussion

**Figure S12.**
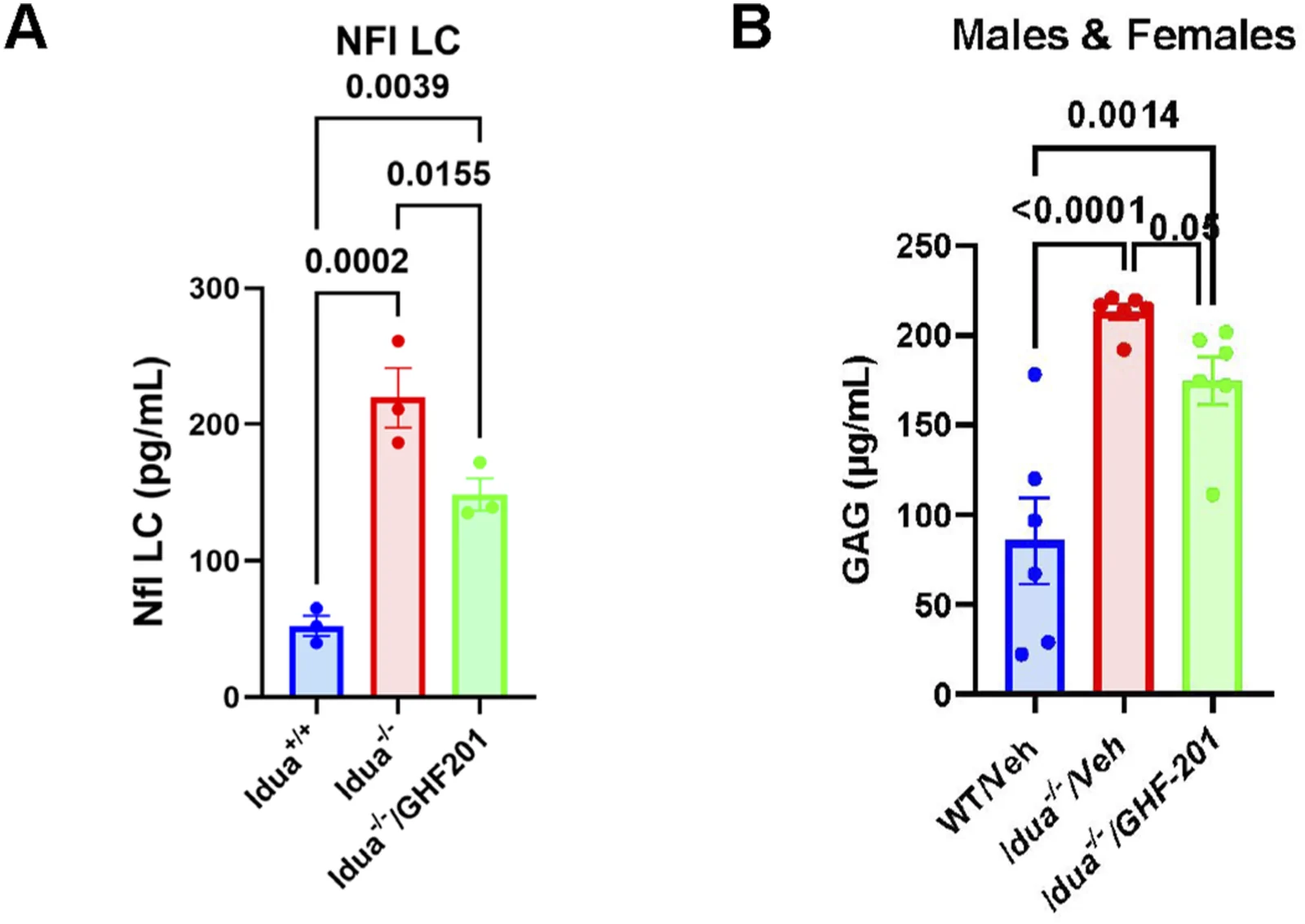
GHF201 reduced NFl LC plasma levels and urine GAG levels in *Idua*^−^*^/^*^−^ mice. **(A)** LC-Nfl in the plasma determined by the Simoa machine using a specific Nfl detection kit. Results from n = 3 mice per group in the groups indicated are shown here. One-way ANOVA with correction for multiple comparisons shows that GHF-201 treatment of *Idua*^*−/−*^ mice reduced their plasma LC-Nfl levels, which are increased compared with the levels in *Idua*^*+/+*^ mice. **(B)** GAGs were quantified in the urine of six mice per group using the Biocolor Blyscan assay. To increase statistical power, because of technical difficulties in obtaining enough urine for the assay, male and female data were pooled. The data are presented as the mean ± SD. *P*-values were calculated using a one-way ANOVA with correction for multiple comparisons.

This study illustrates the therapeutic potential of our novel autolysosomal activator GHF201 for treating MPS-I. We show that independent of enhancing Idua levels or activity, as done by ERT, enhancement of lysosomal function by GHF201 can effectively reduce GAG accumulation (Figs 1A and S12B), ameliorate systemic (Fig 4) and CNS-specific (Figs 6, 7, and 8) biomarkers, and partially rescue motor and cognitive deficits (Fig 2) in the *Idua*^*−/−*^ MPS-I mouse model. These results suggest that the pathology in MPS-I is not solely driven by the impaired trimming of terminal iduronic acid from DS or HS. The accumulation of any lysosomal substrate, including, but not limited to, unprocessed GAGs, can lead to lysosomal swelling and compromise lysosomal activity, leading to collateral damage, which might be as important as the direct deficiency in fully processed GAGs (DS or HS with terminal iduronic acid removed). Lysosomal dysfunction on its own, caused by the accumulation of partially processed GAGs in *Idua*^*−/−*^ mice (or MPS-I patients), can dysregulate mTOR signaling (65, 66), lead to aberrant mitophagy (67), thus enriching cells with dysfunctional mitochondria, and cause lysosomal rupture leading to spilling of potentially toxic lysosomal content into the cytoplasm (68). This cascade of secondary cellular pathology is a well-established driver of storage disease progression, shifting the lysosome from a passive storage sink to an active dysregulator of cellular signaling and homeostasis (69, 70). Therefore, because GHF201 can improve lysosomal function, and autophagic and autolysosomal flux, and reduce lysosomal pH (27), its amelioration of the murine MPS-I phenotype, also instigated by autolysosomal dysfunction, is plausible (71). The translational potential of the GHF201 and Idua–ERT combination: a key finding in our MEF studies (Fig 1B) was the additive increase in cathepsin activity when GHF201 was combined with recombinant Idua (combo treatment in Fig 1). Although Idua–ERT treatment targets the primary enzymatic deficiency in MPS-I, GHF201 primes the lysosomal environment, likely by increasing the overall lysosomal hydrolytic capacity, or improving the maturation of endogenous enzymes. This suggests that GHF201 could serve as a potent adjunct therapy in MPS-I, potentially allowing for lower doses of ERT, or providing benefit to tissues that are typically refractory to enzyme uptake but contribute to disease pathology, such as the brain.

An interesting finding of this work was the divergent effect of GHF201 on cathepsin activity in isolated cells (MEFs [Fig 1] and different brain cell types [Fig 7A and B]) versus intact tissues (liver and joints [Fig 3]): in *Idua*^*−/−*^ MEFs, microglia, neurons, astrocytes, and oligodendrocytes, GHF201 increased lysosomal cathepsin activity, reflecting a restoration of intracellular degradative capacity. Conversely, IVIS imaging of liver and joints suggests that GHF201 decreased cathepsin activity in situ, probably in the ECM of these tissues, bringing it closer to cathepsin activity observed in *Idua*^*+/+*^ mice, thus demonstrating a therapeutic effect. We view this apparent discrepancy not as a contradiction, but rather as evidence of a systemic correction: in MPS-I, excessive GAGs in the ECM stabilize secreted cathepsins, leading to chronic inflammation and tissue damage. Because the ultimate source of extracellular ECM GAGs is intracellular GAGs, by reducing intracellular GAG accumulation, GHF201 eventually also reduces extracellular GAGs, thereby reducing the stabilization and sequestration of cathepsins in the ECM and attenuating pathological extracellular proteolysis. This explanation is based on the premise that the in situ cathepsin signal measured by IVIS is dominated by extracellular/interstitial pool of cathepsins. This premise is well established, as extracellular cathepsins dominate the synovial fluid in joints (72) and the liver secretome (73, 74) and as GAGs act as TLR4 agonists triggering macrophages to secrete cathepsin to the extracellular milieu (e.g., reference (75)). Another, more methodological, explanation for the dominance of extracellular cathepsin signal in situ is that ECM cathepsins are more accessible to GB123, which encounters this extracellular pool first. Access to the intracellular lysosomal cathepsin pool might be rate-limited by GB123 endocytosis into lysosomes. By enhancing lysosomal degradative capacity, GHF201 clears the GAGs, inhibiting the TLR4 inflammatory trigger and consequently inhibiting active secretion of pro-inflammatory proteases, including cathepsins. By restricting lysosomal rupture, GHF201 treatment also attenuates passive cathepsin leakage from compromised lysosomes, which could also compromise the integrity of the plasma membrane (*rejacere* (76, 77)) leading to extracellular cathepsin leakage. Thus, the overall result of GHF201 treatment is a decrease in the extracellular cathepsin pool, which is the dominant pool, even though intracellular lysosomal cathepsins are more active.

At the subcellular level, TEM examination of the liver revealed a striking rescue of organelle ultrastructure after GHF201 administration. Untreated *Idua*^*−/−*^ hepatocytes and resident Kupffer cells exhibited extensive vacuolation dominated by markedly distended, single-membrane-bound, electron-lucent storage vacuoles, the classical ultrastructural hallmark of engorged lysosomes and autolysosomes filled with undigested GAGs (Fig 5C). Rather than inducing net lysosomal exocytosis or uncoordinated substrate spillage into the extracellular space (next paragraph), GHF201 might act by enhancing intrinsic lysosomal hydrolytic capacity and autolysosomal flux to promote the complete intralysosomal degradation of this entrapped GAG cargo. This complete substrate catabolism resolves organellar distension, significantly reducing both the number and volume of these storage vacuoles. Importantly, in Kupffer cells, which act as key drivers of hepatic inflammation and systemic cytokine release via TLR4 sensing of ECM GAGs, degrading GAGs within the lysosomal compartment removes the intracellular storage burden while preventing the extracellular accumulation of TLR4-activating ligands, thereby reducing chronic inflammatory macrophage activation. As suggested above, a lysosomal enhancer such as GHF201 might also be expected to promote lysosomal exocytosis with ensuing extracellular accumulation of lysosomal enzymes, such as cathepsins, and exacerbation of the *Idua*^*−/−*^ pathology. Indeed, in zebrafish models of three lysosomal disorders—sialidosis, mucolipidosis II, and MPS-IVa—enhanced lysosomal exocytosis increased extracellular cathepsins and their mediated proteolysis and TGFβ signaling leading to detrimental effects in the cartilage (78), comparable to the joint aberrations observed in our murine MPS-I model (Fig 5A), which also presented with cathepsin overaccumulation in the joints. Assuming that GHF201 increased lysosomal exocytosis also in joints and liver of *Idua*^*−/−*^ mice, the fact that it overall reduced extracellular cathepsins could theoretically be ascribed to two scenarios: 1. in parallel to the activation of controlled lysosomal exocytosis, GHF201 also enhances lysosomal degradative capacity, thus reducing uncoordinated leakage of cathepsins to the cytosol and extracellular milieu (76), as well as lysosome-dependent cell death, which leads to necroptotic cytosolic content spillage through RIPK1 signaling (77), which might outweigh cathepsin egress by lysosomal exocytosis. 2. A stated above, cathepsin overactivity in the joints and liver of *Idua*^*−/−*^ mice is a secondary result of chronic inflammation, not just direct lysosomal leakage. GAG accumulation triggers the TLR4 signaling pathway, leading to the recruitment of macrophages and neutrophils, which secrete considerable amounts of pro-inflammatory cathepsins. If GHF201 improves lysosomal function and clears GAGs, it effectively removes the primary inflammatory trigger and the recruitment and activation of these cathepsin-secreting immune cells. Thus, GHF201-mediated reduction of interstitial cathepsins in joints and liver likely reflects a secondary systemic reduction in inflammation, rather than changes in the rate of lysosomal exocytosis.

Our untargeted plasma metabolomics analysis (Fig 4) revealed that GHF201 induces a systemic shift toward catabolism. The co-enrichments of both the TCA cycle and amino acid metabolism (specifically histidine, alanine, and aspartate/glutamate) are consistent with induction of anaplerosis by GHF201: the conversion of glutamate to α-ketoglutarate provides essential intermediates required to maintain TCA cycle flux. This indicates that GHF201 enables the cell to bypass the impairment of the lysosomal–mitochondrial metabolic flux, through which the lysosome furnishes the mitochondria with energy substrates, characteristic of the *Idua*^*−/−*^ phenotype. Interestingly, Scarcella et al (79) show that in MPS-IIIb patient–derived fibroblasts, inhibition, rather than activation, of autophagy and AMPK rescues HS accumulation and lysosomal deficiency. These results counter our results in MPS-I mice, according to which enhancement of autophagic flux and catabolism normalizes the aberrant MPS-I phenotype. However, because the intervention in the Scarcella et al (79) study decreased the LC3II/I ratio, it is possible that this curative effect is attributed to enhancement of autophagic flux rather than inhibition of autophagy (inhibition of autophagosome biogenesis, which actually might enhance the flux). Interestingly, the metabolomics shift toward enhanced catabolism observed in GHF201-treated *Idua*^*−/−*^mice mirrors results seen in other storage disorders (glycogen storage disorders III (49), IV (27), Ia, and II [both in revision]), pointing to a generalized, broad-spectrum mode of action of GHF201, which can reestablish cellular homeostasis in the presence of pathological substrate accumulation. In these disorders, the primary pathological driver is energetic compromise paired with the accumulation of undigested, toxic storage material. By acting as a broad-spectrum metabolic modulator, GHF201 stimulates catabolic substrate clearance, accelerating carbohydrate burning to mobilize pathogenic glycogen in GSD III and IV, enhancing lysosomal glycogen degradation to clear accumulated liver G6P and correct hypoglycemia in GSD Ia (*L.G6pc*^*−/−*^ mice), and up-regulating the malate–aspartate shuttle to support mitochondrial oxidative phosphorylation in Pompe disease (GSD II). Importantly, this catabolic stimulation is homeostatic and self-limiting rather than indicative of toxic hypercatabolism: across our in vivo studies, GHF201 treatment consistently corrected disease-associated hypoglycemia, hyperlipidemia, and blunted total energy expenditure without inducing tissue toxicity (as evidenced by unaltered or restored liver ALT and muscle CK levels) or exhausting cellular energy reserves. Thus, rather than causing unchecked hypercatabolism, GHF201 recalibrates cellular fuel use to clear pathogenic storage burdens and reestablish systemic energetic homeostasis.

Perhaps the most significant therapeutic bottleneck in MPS-I clinical management has historically been the inability of systemically administered recombinant enzymes to traverse the blood–brain barrier. Consequently, although conventional ERT manages systemic visceral disease, the progressive neurocognitive decline in patients has remained largely unaddressed. Although recent progress has been made to circumvent this restriction using bioengineered, brain-penetrating lysosomal enzymes (80), these large macromolecular platforms face distinct translational and manufacturing complexities. Similarly, emerging gene therapy strategies, such as AAV-mediated gene transfer (81, 82), or cell-based gene therapy (83), as well as hematopoietic stem cell transplantation (84) offer durable systemic expression but encounter formidable hurdles in achieving widespread, uniform transduction across the dense architectures of the mature CNS, often requiring highly invasive delivery routes. In contrast to these large-scale macromolecular or viral-vectored modalities, our pharmacokinetic (PK) data (Fig S1) demonstrate that a systemically delivered small-molecule approach—specifically the nonaqueous oral solution ND formulation of GHF201—successfully overcomes this anatomical barrier.

The translational significance of these findings for the lysosomal storage disease field lies in the fact that GHF201-mediated clearance operates across multiple scales—resolving tissue histopathology and organelle ultrastructure, while simultaneously driving cell-autonomous enzymatic activation. At the nanoscale level, our ultrastructural TEM examination (Fig 6D) revealed a striking reduction in the number and volume of distended, electron-lucent lysosomes within microglia, indicating that the drug actively relieves lysosomal storage in situ. Furthermore, using cell type–specific flow cytometry coupled with the activity-based probe GB123 (Fig 7A and B), we demonstrated that GHF201 directly up-regulates lysosomal cathepsin activity within isolated neurons, microglia, and oligodendrocytes harvested from adult brains.

Achieving broad, multilineage activation across both neuronal and glial populations is a notoriously difficult hurdle in neurotherapeutic design. By demonstrating that a systemically delivered small molecule can cross the blood–brain barrier, clear ultrastructural storage, activate target lysosomal enzymes across specific neural lineages, and reverse chronic neuroinflammation, our data position GHF201 as a highly promising stand-alone or adjunct candidate to treat the currently intractable neurological components of MPS-I.

The subset of N-glycans significantly up-modulated in *Idua*^*−/−*^ brains and lowered by GHF201 consisted of heterogeneous (or mixed) species. The absence of a dominant species (such as a single Man5 or a specific sialylated complex glycan) suggests that the GHF201 treatment is likely improving overall lysosomal clearance or proteostatic flux. If the subset were enriched in high mannose species, for instance, the *Idua*^*−/−*^ impairment would have specifically affected ER-to-Golgi trafficking or early autophagic vesicles. The fact that mixed N-glycans are elevated in *Idua*^*−/−*^ mouse brains suggests an arrest of glycoprotein turnover, resulting in the accumulation of various biosynthetic intermediates and mature glycoforms within dysfunctional lysosomes. Partial reversal of this N-glycan accumulation by GHF201 suggests that the compound can restore endo-lysosomal flux, consistent with the previous studies (27, 49), by alleviating the degradative bottleneck induced by GAG accumulation, thereby partially reinstating the capacity of endogenous proteases to catabolize the sequestered glycoprotein cargo.

Lysosomes can degrade glycoproteins and glycolipids to release monosaccharides (e.g., mannose, fucose, and GlcNAc), which can be exported to the cytoplasm and salvaged for nucleotide sugar biosynthesis (e.g., GDP-mannose, UDP-GlcNAc). Therefore, in MPS-I cells with dysfunctional lysosomes the steady stream of salvaged sugars to the cytoplasm followed by N-glycan biosynthesis in the ER/Golgi might be restricted. However, this salvage pathway is not the cell’s only source of nucleotide sugars. Because the brain is a high-glucose–consuming organ, the conversion of glucose-6-P to fructose-6-P (glycolysis) also provides a major precursor for the hexosamine biosynthetic pathway (producing UDP-GlcNAc) and the mannose pathway (producing GDP-mannose via Man-6-P and Man-1-P). In the face of a salvage block, as might be the case in MPS-I, cells would typically up-regulate de novo pathways, such as the above-mentioned glycolysis, to maintain essential glycosylation. Consequently, although metabolic efficiency is compromised by the loss of efficient salvage or recycling, biosynthetic flux can be largely sustained. The secretory machinery can continue to glycosylate proteins using de novo nucleotide sugar donors, ultimately channeling these glycoforms into a terminal lysosomal sink where they accumulate because of the failure of catabolic clearance, which, as explained, can be partially restored by GHF201. Importantly, we interpret our observed MALDI signal to be dominated by the storage fraction. In a healthy cell, once a glycoprotein is tagged for degradation, it is rapidly proteolyzed and deglycosylated. Its residence time in the degradative compartment is expectedly very short. In MPS-I, however, the residence time of these glycoproteins in the lysosomal compartment can significantly increase.

Addressing sex dimorphism in the MPS-I mouse model: The observed sex dimorphism—where female mice showed superior behavioral rescue compared with males—aligns with existing literature regarding the epidermal growth factor receptor–testosterone axis in MPS-I models (31, 32, 33). Although this dimorphism appears unique to the murine model and is not reported in human patients, it highlights the importance of sex-stratified analysis in preclinical trials. The robust correction of nonassociative memory and spatial learning in females (Fig 2E) underscores the potency of GHF201 when the baseline GAG synthesis rates are manageable.

In conclusion, GHF201 represents a multimodal approach to MPS-I. Beyond its role as a lysosomal activator, it acts as a metabolic modulator and a potent CNS-penetrant agent. The reduction in systemic biomarkers such as LC-NfL (Fig 4A) and the correction of bone dysplasia (Fig 5A) suggest that GHF201 can address intractable aspects of MPS-I refractory to current standards of care. Future studies should explore the long-term safety of this catabolic activation and its potential for treating other lysosomal storage disorders characterized by impaired autophagic flux.

## Materials and Methods

### Antibodies

Primary antibodies against Akt (#9272), p-Akt (ser473) (#9271), p-mTOR (ser2448) (hybridoma clone D9C2) (#5536), mTOR (#2972), S6 ribosomal protein (hybridoma clone 5G10) (#2217), p-S6 ribosomal protein (ser235/236) (#2211), and beta-actin (hybridoma clone D6A8) (8457S) were from Cell Signaling Technology. Primary antibodies against SQSTM1/p62 (hybridoma clone [EPR4844]) (ab109012), LC3B antibody (hybridoma clone [EPR18709]) (ab192890), Iba1 (ab153696), GFAP (ab7260), and NeuN (hybridoma clone [EPR12763]) (ab279295-100) were from Abcam. PE-conjugated anti-mouse CD11b antibody (117307) was from BioLegend. Primary antibodies against ACSA-2 and O4 were a kind gift from Prof. Oded Behar, the Hebrew University of Jerusalem. Secondary antibodies HRP-conjugated anti-rabbit (7076S), HRP-conjugated anti-mouse (7074S), anti-rabbit F(ab’)_2_ fragment (Alexa Fluor 488 Conjugate) #4412S, anti-mouse Alexa Fluor 594 (CST-8890S), anti-chicken IgG H&L (Alexa Fluor 647) (ab150171) were from Abcam.

### Reagents

GMEM (#11-710-035), FCS (Qualified, A5256701), sodium pyruvate 100 mM (×100, 11360070), 1 mM glutamine (#25030-024), 100X nonessential amino acid solution (#11140050), 2-mercaptoethanol (#11528926), 0.25% trypsin–EDTA (X1, 25200056), Dulbecco’s phosphate-buffered saline without calcium and magnesium (14190144) were from Gibco. RIPA buffer (20-188) and Bicinchoninic Acid Protein Assay Kit (23225) were from Merck. 10-cm dishes (431110) were from Corning. ECL SuperSignal kit (34579) was from Pierce Biotechnology. MES SDS running buffer (NP0002), SeeBlue Plus2 Prestained protein standard ladder (LC5925), and reducing buffers (NP0004) were from Thermo Fisher Scientific (Life Technologies). Antifade with DAPI (#8961), and protease/phosphatase inhibitor cocktail (#5872) were from Cell Signaling Technology.

### Mouse embryonic fibroblasts

Primary MEFs were isolated from *Idua*^*−/−*^ and *Idua*^*+/+*^ mouse embryos at E12.5–13.5 following the protocol in O’Dwyer et al (85). In brief, embryos’ internal organs and heads were removed and the body was trypsinized and homogenized. After cell debris disposal, single cell–containing supernatant was plated in 10-cm dishes containing warm MEF medium (GMEM [G5154; Sigma-Aldrich], 10% FCS, 1 mM sodium pyruvate, 1 mM L-glutamine, 1× nonessential amino acids [11140035; Thermo Fisher Scientific]). Confluent cells (passage 0) were collected and used. MEF viability after GHF201 treatment was determined by the Abcam MTT assay kit (ab211091; Abcam).

### Determination of sGAGs

Total sGAGs were determined in MEFs and in mouse urine using the Blyscan sGAG quantification assay (Biocolor), as per the manufacturer’s instructions.

### Live imaging

MEFs were seeded at 1,400 cells per well and cultured in specialized microscopy-grade 96-well plates (Cellvis P96-1.5H-N) for 24 h at 37°C and 5% CO_2_ with the supplements indicated in Fig 1. Cells were then washed in HBSS, supplemented for 20 min with 1.6 mM Hoechst 33342 and 0.4 mM calcein-AM Green (Thermo Fisher Scientific), and washed again with HBSS. Images were then acquired by the Operetta G1 system at 20× magnification. All assay parameters (including the acquisition exposure times, objective, and the analysis parameters) were kept constant for all assay repetitions. Images were analyzed by the Harmony image analysis software version 1.0. Downstream analysis included removal of wells with outlier values using GraphPad Prism (version 10.2.3)’s ROUT method (Q = 10%), feature selection by backward feature elimination, and normalization to *Idua*^*+/+*^ MEF levels before statistical analysis (86).

### PK profiling

The PK profile of the ND formulation of GHF201, ND-GHF201, developed by Lyotropic Delivery Systems Ltd. for p.o. administration, was determined in several tissues and in serum derived from *Idua*^*−/−*^ mice using liquid chromatography–tandem mass spectrometry as described previously by us (27).

### Animals and in vivo tests

*Idua* knockout *Idua*^*−/−*^ mice were procured from the Jackson Laboratory (strain B6.129-*Idua*^*tm1Clk*^/J). C57BL/6 littermate mice were the genetic background of these transgenic mice and used as a wild-type (*Idua*^*+/*+^) control. All in vivo experiments were approved by the Hebrew University Faculty of Medicine IACUC (approval number 17201). n = 6–8 mice/arm were used. GHF201 was orally administered by daily oral pipetting of 20 μl liquid ND-GHF201 formulation at 70 mg/kg. 20 μl unloaded ND formulation served as a control. Mice were treated from the age of 3 mo to the age of 8.5 mo. Animals’ weight was recorded every week. A weight decrease of >10% between biweekly weightings, or >20% from treatment initiation led to removal of the animal from the study. Based on our experience in animal models of other storage disorders (glycogen storage disorder types III (49), IV (27)) where deviation from wild type of various motor–behavioral parameters was maximal at 8–10 mo of age, we determined these parameters at the age of 8.5, shortly before euthanasia: open-field moving distance, front paw grip strength using a grip-strength meter (Columbus Instruments). Cognitive function in the open field was assessed by comparing habituation time, which evaluates long-term, nonassociative, spatial memory (36, 37). In this test, a mouse is placed in an open field over two consecutive days and is allowed to explore for 5 min. The longer the animal spends exploring its environment after the first day, the more compromised its nonassociative memory is considered. We assessed spatial learning by testing mice daily over five consecutive days in the MWM (38). MWM measures change over time in latency to escape to a submerged platform as an inverse estimate of spatial learning. This escape latency was measured over 5 d so that learning can be assessed.

### IVIS analysis

Mice were i.v. injected with 100 μl of GB123 (0.55 mM) through the tail vein. 24 h later, mice were swiftly euthanized by cervical dislocation and liver and left legs were collected and placed on ice in PBS. The liver was sectioned: one section was snap-frozen in liquid nitrogen and used for on-gel assays. In the in vivo on-gel assay (Fig 3B), proteins from this section were extracted by homogenizing the tissue in 1.5-ml Screw-Cap tubes filled with stainless steel beads (SSB16; Next Advance, Inc.) and RIPA buffer using a bead homogenizer (Bullet Blender Storm—BBY24M; Next Advance) at speed 8 for 3 min. Total protein extracts were separated by 12.5% SDS–PAGE and visualized by scanning the gel with a Typhoon scanner FLA 9500 at excitation/emission wavelengths of 635/670 nm (GE Healthcare Bio-Sciences AB). For the direct ex vivo on-gel assay (Fig 3B), lysates were incubated with GB123 (2 μM), for 90 min. Equal protein amounts were separated on 12.5% SDS–PAGE and scanned by the Typhoon scanner. The other section of the liver and the left legs were placed on ice in PBS and used for fluorescence analysis in the IVIS 200(PerkinElmer) with a Cy5.5 filter. Liver sections were used directly, whereas in the left legs, skin and fur were first exfoliated in the knee joint area before IVIS imaging.

### Mouse plasma metabolomics

LC-MS metabolomics analysis was performed on mouse plasma as follows. Plasma (20 μl) was diluted 10 times with 180 μl extraction buffer (50% methanol, 30% acetonitrile, 20% water), mixed in a thermoshaker (ThermoMixer C, Eppendorf) at 2.4*g* for 10 min, and centrifuged at 4°C for 20 min at 16,160*g*. Supernatants were stored in HPLC glass vials at −80°C until LC-MS analysis. For the LC-MS, Dionex UltiMate 300 high-performance liquid chromatography (UPLC) system coupled to an Orbitrap Q Exactive plus Mass spectrometer (Thermo Fisher Scientific) with a resolution of 70,000 at 200 mass/charge ratio (m/z), electrospray ionization in the HESI source, and polarity switching mode to enable both positive and negative ions across a mass range of 70–1,000 m/z was used. The UPLC setup included a ZIC-pHILIC column (SeQuant; 150 mm × 2.1 mm, 5 μm; Merck) with a Sure-Guard filter (SS frit 0.5 μm). Five microliters of the plasma extracts was injected, and the compounds were separated using a mobile phase consisting of 20 mM ammonium carbonate (adjusted to pH 9.2 with 0.1% ammonium hydroxide) as solvent A and acetonitrile as solvent B. The method started at 80% B (20% A) and was held until 17.0 min, followed by a step to 20% B (80% A) at 17.0 min. The mobile phase was then returned to 80% B at 17.1 min and maintained until 26.0 min for column re-equilibration. The flow rate and column temperature were maintained at 0.2 ml/min and 45°C, respectively, for a total run time of 26 min. All metabolites were detected using mass accuracy below 5 ppm. Thermo Xcalibur was used for the data acquisition. Data processing and analysis were carried out using Compound Discoverer version 3.3.2.31. In total, 167 metabolites were identified through this platform. The identification process used an in-house spectral library and MS2 data comparison with the mzCloud and MzVault databases. Metabolomics data were analyzed using MetaboAnalyst 6.0. Metabolites with more than 33% missing values were removed, and metabolites that were near-constant throughout the groups were detected using a 20 IQR variance filter. Samples were normalized to the healthy control group and scaled using logarithmic normalization. Individual metabolite peak areas (areas under the curve) derived from LC-MS analysis were exported and uploaded to MetaboAnalyst 6.0 for data preprocessing and downstream statistical evaluation. Because a uniform initial volume of plasma (20 μl) was used for every sample extraction, software-based row-wise sample normalization was omitted. For univariate statistical analyses—specifically *t* tests for selecting heatmap metabolites—peak area data were subjected to log transformation (base 10) with data scaling set to “None.” All relative changes were calculated using the healthy control (*Idua*^*+/+*^) group as the reference baseline. Conversely, for multivariate statistical modeling, the log-transformed dataset was also preprocessed using auto scaling (mean centering followed by division by the SD of each variable) to account for magnitude variations among high- and low-abundance features. Statistical significance for univariate comparisons was determined using two-tailed Student’s t tests, with thresholds for significance set at *P* < 0.05.

### Micro-CT scanning

Micro-CT scans were performed using MILabs U-CT Low-Dose Ultra-High-Resolution Micro-CT System with specific parameters: total body magnification, single energy source, and normal scan mode (360° rotation, 50 kV voltage, 0.21 mA current, one projection per step with a 1° step angle, 75-ms exposure time, and 1 × 1 binning) with a fixed 100-μm aluminum filter and a standard 400-μm aluminum filter. Image analysis was conducted using Imalytics software (version 3.1.1.8).

### Transmission electron microscopy

Whole brain and small pieces of liver tissue were fixed in a solution containing 2% PFA, 2.5% glutaraldehyde EM grade in 0.1 M sodium cacodylate buffer, pH 7.3, for 2 h at RT followed by 24 h at 4°C. The brain was sliced sagittally using vibratome to 200-μm slices, and slices were reduced in size to the regions, which include the cortex and the hypothalamus. Samples were washed four times with sodium cacodylate and postfixed for 1 h with 1% osmium tetroxide, 1.5% potassium ferricyanide in sodium cacodylate, and washed 4 times with the same buffer, followed by dehydration with graded series of ethanol solutions (30%, 50%, 70%, 80%, 90%, 95%) for 10 min each and then 100% ethanol 3 times for 20 min each, followed by two changes of propylene oxide. Samples were then infiltrated with series of epoxy resin (25%, 50%, 75%, 100%, 24 h in each) and then flat-embedded at the desired orientation, and polymerized in the oven at 60°C for 48 h. The blocks were sectioned by an ultramicrotome (Ultracut E, Reichert-Jung), and sections of 80 nm were obtained on 200-mesh carbon–formvar grids and stained with uranyl acetate and lead citrate. Sections were observed by JEOL JEM-1400 Plus Transmission Electron Microscope, and images were taken using a Gatan Orius CCD camera.

### Western immunoblotting

Brain and liver tissues were homogenized in RIPA buffer (Merck) on ice using a Teflon pestle and a Jumbo Stirrer (Thermo Fisher Scientific). The homogenates were centrifuged at 17,000*g* for 30 min at 4°C. The supernatant was collected, and the protein concentration was estimated by Bicinchoninic Acid Protein Assay (Merck). The samples were subjected to polyacrylamide gel electrophoresis, followed by wet transfer onto a polyvinylidene fluoride membrane (Bio-Rad Laboratories). Nonspecific sites were blocked by either 5% dried skimmed milk or 5% BSA in Tris-buffered saline with Tween-20, containing 135 mm NaCl, 50 mm Tris, and 0.1% Tween-20, pH 7.4, for 2 h at room temperature. polyvinylidene fluoride membranes containing transferred proteins were incubated with a primary antibody overnight at 4°C under shaking. The following primary antibodies were used: anti-Akt (1:1,000), anti-phospho-Akt (1:1,000), anti-phospho-mTOR (1:1,000), anti-mTOR (1:1,000), anti-S6 ribosomal protein (1:1,000), anti-phospho-S6 ribosomal protein (1:1,000), anti-beta-actin (D6A8) (1:1,000), anti-SQSTM1/p62 antibody [EPR4844] (1:1,000), anti-LC3B antibody [EPR18709] (1:1,000). After exposure to primary antibodies, the membranes were washed with Tris-buffered saline with Tween-20 and incubated with anti-mouse/rabbit HRP secondary antibody for 1 h at room temperature. Specific binding of the protein of interest was detected using ECL substrate (Bio-Rad Laboratories). The bands were visualized using the Bio-Rad ChemiDoc imaging system (Hercules). For quantification, we analyzed the gray value of the protein using Bio-Rad Image Lab software. We normalized it to that of the corresponding internal controls.

### Image scanning of brain sections

Coronal sections of the cortical region (20 μm thick) were processed for dual immunofluorescence. In brief, the sections were washed with 1xPBS for 1 min. The sections were then permeabilized with 0.2% Triton X-100 (Merck) in 1xPBS for 10 min, followed by blocking with 5% goat serum (Vector Laboratories) in 0.1% Triton X-100 and 1% BSA (MP Biomedicals) in 1xPBS for 2 h. After blocking, sections were washed three times (10 min in each wash) with 1xPBS. Sections were then incubated overnight at 4°C with rabbit anti-NeuN (1:100), mouse anti-Iba1 (1:100), and chicken anti-GFAP (1:500). Then, the sections were rinsed with PBS and incubated with anti-rabbit Alexa Fluor 488 (1:1,000), anti-mouse Alexa Fluor 594 (1:1,000), and anti-chicken Alexa Fluor 647 (1:1,000) secondary antibodies for 2 h in the dark. After incubation with secondary antibodies, the sections were washed with PBS three times and mounted on glass slides with DAPI. Images were then captured using the SlideView VS200 slide scanner (Olympus) at a 40× magnification. The depth intervals (Z-intervals, the vertical distance between consecutive image planes during the Z-stack acquisition performed by the scanner) of each image were automatically determined by the instrument. The mean fluorescence intensity was calculated using ImageJ software.

### Flow cytometry

Lysosomal caspase activity in microglia, neurons, astrocytes, and oligodendrocytes was determined by flow cytometry as follows (87): resected brain sections were rapidly frozen by being submerged in −40°C isopentane for 20 s. Sections were kept frozen at −80°C until use. Before use, dissected tissue was transferred into ice-cold buffer (PBS, 1% BSA, and 0.1% sodium azide) and finely minced (∼100 cuts in orthogonal directions). Samples were centrifuged at 110*g* for 2 min at 4°C, and the supernatant was discarded. The tissue pellet was resuspended in enzyme solution (Accutase) and incubated for 30 min at 4°C with gentle mixing. After enzymatic digestion, samples were centrifuged (960*g*, 2 min, 4°C) and pellets were resuspended in cold PBS. Cell suspensions were then filtered sequentially through 70- and 40-μm cell strainers to remove debris and aggregates. The final single cell–filtered suspension (∼1 ml) was maintained on ice. This cell suspension was stained with 0.25 μM GB123 for 1 h and subsequently washed three times with FACS buffer. Cell suspensions were then fixed and permeabilized with ice-cold ethanol to a final concentration of 50%. Samples were incubated on ice for 15 min and centrifuged at 1,700*g* for 4 min at 4°C. Supernatants were carefully removed, leaving minimal residual volume to avoid cell loss. Cell pellets were resuspended and blocked in 1% BSA for 30 min at 4°C. Before staining, aliquots of cell suspension were used to prepare control samples, including unstained, single-labeled, and secondary antibody controls. For the main test samples, cells were incubated with antibodies against CD11b conjugated to PE to label microglia (#117307; BioLegend, 0.25 μg per 10^6^ cells in 100 μl), NeuN conjugated to Alexa Fluor 488 to label neuronal nuclei (ab190195; Abcam, 1:500), ASCA2 (1:100 hybridoma supernatant) followed by staining with secondary anti-IgG antibody conjugated to Alexa Fluor 594 to label astrocytes, and O4 (1:100 hybridoma supernatant) followed by staining with secondary anti-IgG antibody conjugated to Alexa Fluor 594 to label oligodendrocytes. After immunostaining and GB123 staining, acquisition was made in the BD LSRFortessa flow cytometer using the following configurations: for NeuN (conjugated to Alexa Fluor 488), laser line 488 nm, 530/30 BP filter with 505 LP dichroic mirror; for CD11b (conjugated to PE), laser line 561 nm, 586/15 BP filter without dichroic mirror; for both the ASCA2 and O4 antigens labeled with secondary antibody conjugated to Alexa Fluor 594, laser line 561 nm, 610/20 BP filter with 600 LP dichroic mirror; for GB123, laser line 633-640 nm, 675/50 BP filter without a dichroic mirror.

### Spatial profiling of brain N-glycans

In brief, brains were dissected and swiftly fixed in 10% formalin (pH 7.3), dehydrated by increasing concentrations of ethanol, permeated with xylene, and paraffin-embedded. These formalin-fixed paraffin-embedded blocks were sectioned into 4-μm slices and mounted onto positively charged microscope slides. Tissue-mounted slides were heated for 1 h at 60°C, dewaxed, dried, and antigen-retrieved by citraconic anhydride matrix. Subsequently, tissues were digested with PNGase F and coated with alpha-cyano-4-hydroxycinnamic acid matrix. The N-glycans produced by the PNGase F treatment were ionized by traveling wave laser and recorded by time-of-flight mass spectrometry. A detailed step-by-step protocol can be found in Hawkinson (88) and Stanback et al (89).

### Statistical analysis

Sample sizes were determined based on the effect sizes of key parameters previously measured (e.g., open-field moving distance (27)), as detailed in reference (90). These sample sizes were sufficient to provide the acceptable statistical power of at least 80%. In in vivo studies, the experimenters were blinded to treatment group allocations because animals were encoded. Mouse randomization of *Idua*^*−/−*^ mice was obtained by pooling litters from mice born a few days apart and dividing pups with similar weights into vehicle- and GHF201-treated arms. Statistical significance in experiments involving >2 groups was determined by one-way ANOVA with Tukey’s post hoc test. *P*-values under 0.05 were considered significant, and *P*-values between 0.05 and 0.1 were considered a trend. However, we treat *P*-values as a context-dependent value and not as an arbitrary threshold (91, 92). For instance, because extremely small sample sizes, occasionally used in this work (n = 3), result in low statistical power, a higher *P*-value threshold sometimes can allow to identify potentially significant differences between groups. Specifically, significance of differences between groups in the MWM was determined by one-way ANOVA with repeated measures. In the metabolomics analyses, to compare groups, two-tailed t tests were performed and raw *P*-values were used to define significant metabolites. For metabolite set enrichment analysis and pathway enrichment predictions, raw *P*-values were used as well. For metabolomics visualization of metabolites, s-PLS-DA (based on 2 components) and metabolite set enrichment analysis and visualization were performed and plotted using MetaboAnalyst 6.0.

## Supplementary Material

Reviewer comments

## Data Availability

All data generated or analyzed during this study are included in this published article and its supplementary information files. The raw datasets supporting the findings of this study, specifically the raw plasma metabolomics and raw brain glycomics data, are provided as Source Data files linked to Figs 4 and 8, respectively. Any additional materials or inquiries are available from the corresponding author upon reasonable request (or Kakhlon, ork@hadassah.org.il).
