# Supplementary material for: Autolysosomal enhancer GHF201 attenuates pathophysiology in a mucopolysaccharidosis type I mouse model
Source: Life Sci Alliance. 2026 Oct 6;9(12):e202603750. doi: 10.26508/lsa.202603750 (PMC13646730; doi:10.26508/lsa.202603750)
Supplement: Supplementary file 1 [file LSA-2026-03750_review_history.pdf]

Autolysosomal enhancer GHF201 attenuates pathophysiology in a mucopolysaccharidosis-I mouse model

Pradeep Kumar, Emmanuelle Merquiol, Talya Avraham, Reut Sinai-Turyanski, Pankaj Singh, Roberto Ribas, Sahar Sweetat, Uri Sprecher, Noam Muchtar, Manjeet Gupta, Kumudesh Mishra, Shashank Ojha, Zhana Yekhtin, Hava Glickstein, Ori Shalev, Alexandra Eliassaf, Anna Morshina, Hadas Segev-Yekutiel, Sharon Garti, Nissim Garti, Hanna Rosenmann, Miguel Weil, Matthew Gentry, Galia Blum, and Or Kakhlon
DOI: https://doi.org/10.26508/lsa.202603750

Corresponding author(s): Or Kakhlon, Hadassah-Hebrew University Medical Center and Pradeep Kumar, Hebrew University of Jerusalem

**Review Timeline:**		
	Submission Date:	2026-04-28
	Editorial Decision:	2026-06-23
	Revision Received:	2026-07-24
	Editorial Decision:	2026-08-25
	Revision Received:	2026-09-14
	Accepted:	2026-09-16

Scientific Editor: Sarita Hebbar

Transaction Report:

(Note: With the exception of the correction of typographical or spelling errors that could be a source of ambiguity, letters and reports are not edited. The original formatting of letters and referee reports may not be reflected in this compilation.)

June 23, 2026

Re: Life Science Alliance manuscript #LSA-2026-03750-T

Prof. Or Kakhlon
Hadassah-Hebrew University Medical Center
Neurology
Israel

Dear Dr. Kakhlon,

Thank you for submitting your manuscript entitled "The autolysosomal enhancer GHF201 rescues pathophysiology in a mucopolysaccharidosis-I mouse model" to Life Science Alliance. We apologise for delay in communicating a decision due to reviewer availability issues.

Your manuscript was revised by two reviewers. As you will note the reviewers are largely positive about the results. That said, the reviewers have raised some concerns and highlighted aspects of that manuscript that could be strengthened.

In line with the overall assessment of the reviewers, we invite you to submit a revised manuscript.
-We agree that your revised manuscript must resolve all the reviewers' points. These relate to completeness, accuracy, and scientific rigour of this work.
-Your revised ms must moderate your conclusions on the effect of GH-201 on behaviours (Reviewer 1, point on behaviours), cathepsin (Reviewer 2, point 4) and rescue of glycans (point 10). Related to this, we encourage you to adjust your title accordingly.
-We concur with both reviewers that you must carefully report on the comparisons of ERT with that of GH-201, better explain the confounding result on combo therapy on cell viability (Reviewer 2, point 1) and provide evidence of the effect of the combo therapy (in MEFs, Reviewer 1, second paragraph and Reviewer 2, point 2).

When submitting the revision, please include a letter addressing the reviewers' comments point by point.

I would be happy to discuss the revision in more detail via email or phone/videoconferencing. Please let me know which option you prefer, if any.

To upload the revised version of your manuscript, please log in to your account: https://lsa.msubmit.net/cgi-bin/main.plex

You will be guided to complete the submission of your revised manuscript and to fill in all necessary information. Please get in touch in case you do not know or remember your login name.

The typical timeframe for revisions is three months. Please note that papers are generally considered through only one revision cycle, so strong support from the referees on the revised version is needed for acceptance.

While you are revising your manuscript, please also attend to the below editorial points to help expedite the publication of your manuscript. Please direct any editorial questions to the journal office.

Thank you for this interesting contribution to Life Science Alliance. We hope that the comments below will prove constructive as your work progresses, and we are looking forward to receiving your revised manuscript.

Sincerely,

Sarita Hebbar, PhD
Scientific Editor
Life Science Alliance
http://www.lsajournal.org

A. THESE ITEMS ARE REQUIRED FOR REVISIONS

-- A letter addressing the reviewers' comments point by point.

-- An editable version of the final text (.DOC or .DOCX) is needed for copyediting (no PDFs).

-- High-resolution figure, supplementary figure and video files uploaded as individual files: See our detailed guidelines for preparing your production-ready images, https://www.life-science-alliance.org/authors

-- Summary blurb (enter in submission system): A short text summarizing in a single sentence the study (max. 200 characters including spaces). This text is used in conjunction with the titles of papers, hence should be informative and complementary to the title and running title. It should describe the context and significance of the findings for a general readership; it should be written in the present tense and refer to the work in the third person. Author names should not be mentioned.

-- By submitting a revision, you attest that you are aware of our payment policies found here: https://www.life-science-alliance.org/copyright-license-fee

B. MANUSCRIPT ORGANIZATION AND FORMATTING:

Full guidelines are available on our Instructions for Authors page, https://www.life-science-alliance.org/authors

We encourage our authors to provide original source data, particularly uncropped/-processed electrophoretic blots and spreadsheets for the main figures of the manuscript. If you would like to add source data, we would welcome one PDF/Excel-file per figure for this information. These files will be linked as supplementary "Source Data" files.

IMPORTANT: It is Life Science Alliance policy that if requested, original data images must be made available. Failure to provide original images upon request will result in unavoidable delays in publication. Please ensure that you have access to all original microscopy and blot data images before submitting your revision.

Reviewer #1 (Comments to the Authors (Required)):

Kuma et al. present an extensive study into the use of GHF-201 for the treatment of the MPS I mouse. The topic is of great importance, particularly in relation to the yet unmet need for treatment of the neurological manifestations of the disease. I found the study to be generally well defined, however with such extensive data, I did struggle a little at times to follow along and found some important points got lost, particularly in the figures.

One exciting piece of data was the improvement in lysosomal activation when GHF-201 and Ald were used together. I was therefore surprised that this combined therapy was not investigated further in the mouse model as a potential adjunct therapy. Even in the MEFs, neither GHF-201 or Ald normalised GAG on their own, but would the combined Ald and GHF-201 further reduce GAG toward normal concentrations? I think this would have been an important point to show and would have further enhanced the argument that a combined therapy approach is beneficial, linking back to an objective diagnostic measure for patients. Can the authors please explain why this did not occur?

The CNS results were perhaps the most exciting in the manuscript and the most relevant to the field given the lack of current drugs that successfully cross the BBB. Figure 6 shows clear normalisation of neuroinflammation which could be a major advancement to treating neurological forms of the disease, but very little is mentioned in either the results or discussion. I think these results need to be highlighted more. This is great work that is currently getting lost in the other data.

General comments:
The figures are very busy making them difficult to look at and interpret. I worry that important information is getting lost with so many panels per figure that don't necessarily go together, e.g. the pharmacological data and behavioural studies in Figure 2. I think it would be beneficial to keep and enhance the most important panels and put the other figures into the supplementary material. This would also simplify the Figure legends which are currently very long and - in many cases - give more information than what is provided in the text.

It is referred to as GHF-201 in the text, but GHF201 in most of the figures and the title. It would be good to be consistent.

Glycosaminoglycans is abbreviated to GAGs, however GAG and GAGs are interchanged throughout.

Specific comments/suggestions for the manuscript:
Introduction:
Line 3: define the abbreviation CNS in the first instance.

Line 13: It may be worth starting a new paragraph for the pathological features. I also suggest moving the incidence sentence to the start of the paragraph which may flow better than having it between the peripheral and CNS pathology.

Row 14: Consider changing to "prominent peripheral features."

Row 15: Consider changing "milder" to attenuated, as per current accepted nomenclature.

Reference 16 is incorrect.

At the start of the third paragraph (line 24) the abbreviation SoC is not defined.

Line 27: Should this be hematopoietic stem cell transplant (HSCT)? Reference should come after this sentence.

Results:
GHF-201 and ERT reduce GAG levels in MEF
I did find this section a little hard to follow, the Figure legend actually explains better than the text. I am not sure I understand the rationale for the addition of 150 µM GHF-201 given GAG concentrations were similar by 50 µM and stabilised. Addition of the recombinant enzyme did not normalise GAG concentrations, which should be noted.

GHF-201 increases cathepsin activity additively with ERT in Idua-/- mouse fibroblasts.
Typo on line 5 - caused by GHF-201...

GHF-201 improves behavioural and motor deficits in Idua-/- mice
I think the authors need to be careful with their interpretation of the behavioural studies. Only the open field showed a true sex difference, with males not having any improvement with treatment. Grip strength in affected male mice was not decreased compared to WT; this is not a poor response to treatment, but a lack of deficit to begin with and should be noted. In the Water Cross maze, the treated mice showed a similar escape latency to WT for both sexes. This is not reflected in the text.

Discussion:
Non-reducing end iduronic acid is not removed in MPS I, that is where the deficiency lies. Please correct in line 5 and 8.

There needs to be more relation back to the literature. For example, the cascade of secondary cellular pathology is widely documented and should be referenced. I think it would also be good to compare/contrast the results in this manuscript back to those for other types of treatment such as ERT and gene therapy.

On the third page of the discussion, the abbreviation BBB has not been defined previously.

Methods:
Were the mice embryos used in the MEFs from the same colony described in the "Animals and in vivo tests"?

Mouse plasma metabolomics
There is no information on how the plasma samples were extracted. This needs to be included or referenced.

I'm not sure I understand the LC gradient. It started at 80% B and ramped to 20%B at 15 min? Then what? Was it held there or did it go straight back to 80% B for equilibration until 26 min? Please clarify.

"Samples were normalized to the HC group..." I am assuming that HC is healthy control group? How was this normalisation done? Comparison of the area under the curve? Using an internal standard? More information is needed for others to be able to follow.

Statistics
While I appreciate that small sample sizes do reduce the power of statistics, I do not believe increasing the p value threshold above 0.05 is appropriate. To have this as a 'sliding scale' feels like moving the target to fit the hypothesis, which is not scientifically sound. There is no real rationale for the changes within the manuscript and I would think there would be a need for orthogonal validation that the reported significant changes are not false positives due to the high threshold. It is perfectly acceptable to talk about trends that do not reach significance (especially when they are 0.06 or 0.07) but they are not significantly different, and this should be noted along with the limitation of sample size.

Figure 2:
There are abbreviations in the Figure legend that have not been defined in full.
The y-axes for the open field graphs would be better in cm.
The examples of tracking and heat maps would be better in the supplementary material.
The statistics in the Figure legend for the Water Cross Maze are unclear and not indicated on the Figure, and the wording is a little confusing, e.g. the sentence below.
"...reduction in escape latency over time, or spatial learning, is reduced in Idua-/-mice as compared to Idua+/+ ($p<0.03$) and improved by GHF-201..." Please consider changing the wording for clarity.

Figure 3:

Label the graphs in panel B.
Abbreviations in the Legend that are not defined.

Figure 4:
There is no key for panels G and H.
I would suggest removing the ROC curve, as it does not add anything more than the PLS plot.
Panel D says Empty ND (please define in the legend) and GHF 201, however in the legend it only mentions WT and affected mice.

Figure 8:
No title on the x-axis on panel B.
Label panel J. Are these two different brains? Two different sections?

Supplementary material:
To be clear, rapamycin and 3-MA were administered to affected littermates that had not received treatment in the preceding time?
It is stated that grip strength was decreased by 3-MA, however this is the same as what is seen in affected mice, so is it not that 3-MA had no effect?
How is open field influenced by grip strength? (paragraph 3)

Reviewer #2 (Comments to the Authors (Required)):

In this paper, Kumar et al have described the usage of GHF-201, an established lysosomal activator drug for the potential treatment of MPS-1 mouse model. They utilize cell culture and in-vivo tissue studies to study the efficacy of the drug. While overall the results are encouraging, there are sections, where the interpretation and a follow up experiment is not done to reach the stated conclusion. Some results presented in the paper is also not interpreted well enough to justify what was observed.
1. One of the main conclusions of the paper as mentioned in the discussion section is to use this drug alongside already existing enzyme replacement therapy. The idea primarily comes from the result presented in Fig 1B. In this result, the focus is made onto the activity state of cathepsin B, which seems to be improved by the treatment of GHF-201, however looking at the calcein staining, seems the cell viability is severely compromised in the combo treatment. The author shows a graph (Fig. 1C) that stand alone treatment with GHF-201 does not impact cell viability. However, no data was presented to show viability of the cells in the combo treatment. And the Calcein data raises a doubt whether the combo treatment affects cell survival significantly even if it is able to induce cathepsin B activity. In the absence of such data, the conclusion and using the drug in a combo therapy claim cannot be made.
2. In the same figure a panel (Fig 1A) shows the GAG concentration decrease in knockout and WT cells with the ERT and new drug. What about the combo? How is the GAG levels respond to the combo treatment? Secondly, looking at this result, seems beyond 50 uM dose there is not really any effect of the drug on the GAG reduction. Therefore, the conclusion that GAG reduction happens in a dose dependent manner should emphasize this observation and its potential translational implication/drawbacks
3. Minor point: Fig 2 panel C, place the genotypes on the open filed pictures for a quick and better visualization by the reader
4. Fig 3, panel B - The results shown somehow does not correlate with the quantification shown and hence the conclusion presented in the result section ("IVIS imaging and on gel studies demonstrate attenuation of tissue cathepsins by GHF-201"). The on-gel in-vivo result clearly demonstrates that Idua-/-/GHF-201 lane barely has any detectable cathepsin, compared to WT. However, the quantification shows that this is significantly indistinguishable from WT. How was the quantification done to get this opposite conclusion? Where is the loading control for this assay?
5. The mobility shift of cathepsin that is shown in this on-gel assay is contributed to the hyperglycosylation of cathepsin B. Interestingly the WT does not have this. Was hyperglycosylation confirmed using some sort of quick biochemical assays such as EndoH or PNGase F treatment? The authors explain this as a result of lysosomal dysfunction. As the whole point of the GHF-201 treatment is to improve lysosomal function, it is surprising that the treatment did not cause any change (even if not significant but a trend towards lowering this hyperglycosylation of cathepsinB). The author should explain this drawback in the discussion section.
6. Fig 4. The metabolomics study clearly shows that the treatment has some sort of effect that is different than WT metabolism. Interestingly the shift is towards more catabolism as seen in other diseases when treated with GHF-201. This implies this maybe a general effect of GHF-201 rather than specific for MPS-I during any pathological conditions. Also, too much catabolism could be detrimental. How in other diseases this shift helped in restoring cellular homeostasis and not any detrimental effect needs to be pointed out in the discussion.
7. Minor point: Bone dysplasia pictures Fig 5A is not convincing to look at the effect of GHF-201. The knockout and the treatment bones look very similar. Is it possible to clearly label where the measurement was taken to produce the graph next to it? Same to do for the supplementary figures for the treatment with Rapamycin and 3MA.
8. Striking results is the reduction of vacuolar structures as shown by EM in the liver and Kupffer cells. Not so much went into the discussion for these results. Please explain in the discussion how do you think GHF-201 may contribute to this phenotype and how this may correlate with the pathogenesis reduction in MPS-I. What type of vacuoles they are? Lysosome vs other vacuoles (i.e. double vs single membrane bound vacuoles as can be easily seen in EM)

9. Similar to the vacuolar structures in the brain also altered in the brain (Fig. 6) i.e. less vacuolar structures in the brain with the treatment. As this effect is seen in multiple tissues - the relevant biology and an explanation of the effect of GHF-201 is warranted. Also the arrows are not clear in this figure, make them prominent to see the difference in the vacuoles numbers. Right now the histology pictures do not any difference to the eye, unless we look at the quantification.
10. The paper title does not fully reflect the content of the paper. The treatment certainly doing something in the knockout mouse and, in some cases, it is not sure whether it is a 'rescue' such as looking at the plasma metabolomics data or glycan data (Fig. 8). These data showed that the treatment cohort is different than the knockout cohort but definitely not as the same as WT cohorts. All could be said some phenotypes were altered by this treatment and in some cases some pathophysiological rescues was observed (bone dysplasia result). The title needs to be changed to emphasize this.
11. Was there any tox study done in the mouse model in the WT mouse to show that it does not have any toxic effect in animal model?

Reviewer #1 (Comments to the Authors (Required)):

Kuma et al. present an extensive study into the use of GHF-201 for the treatment of the MPS I mouse. The topic is of great importance, particularly in relation to the yet unmet need for treatment of the neurological manifestations of the disease. I found the study to be generally well defined, however with such extensive data, I did struggle a little at times to follow along and found some important points got lost, particularly in the figures.

One exciting piece of data was the improvement in lysosomal activation when GHF-201 and Ald were used together. I was therefore surprised that this combined therapy was not investigated further in the mouse model as a potential adjunct therapy. Even in the MEFs, neither GHF-201 or Ald normalised GAG on their own, but would the combined Ald and GHF-201 further reduce GAG toward normal concentrations? I think this would have been an important point to show and would have further enhanced the argument that a combined therapy approach is beneficial, linking back to an objective diagnostic measure for patients. Can the authors please explain why this did not occur?

Response: We thank the reviewer for this insightful comment. The in vitro additivity between GHF201 and Aldurazyme (Ald) is indeed compelling, and evaluating this combination in vivo is our immediate next step. Our strategy prioritizes establishing the standalone in vivo efficacy of a novel small molecule before introducing the complexities of a combination regimen. This systematic workflow mirrors recent pre-clinical validation pipelines in related glycogen and lysosomal storage disorders (Gaspar et al., 2024 (PMID: 39171753); and our own: Mishra et al., 2024 (PMID: 39199279); Sprecher et al., 2026 (in revision, https://ssrn.com/abstract=6050465)). This sequence is also strictly anchored in the 3Rs principle (Reduction). A premature in vivo combination study would require a vastly expanded animal cohort to navigate numerous unresolved pharmacological variables, such as dosing schedules (co- vs. pre-administration), delivery routes (e.g., injection vs. formulation in chow (Ullman et al., 2024 (PMID: 38232139)), and dose-de-escalation to capture true additivity or synergy. Establishing a robust standalone baseline for GHF201 in the current manuscript provides the necessary ethical and scientific foundation to properly design these subsequent multi-arm combination trials.

We also thank the reviewer for the justified comments and observations on our MEFs studies. While Aldurazyme (Ald) significantly reduced sGAGs - clearing over 50% of the pathological excess (from 5.2 down to 4.5 µg/mL, against a 3.85 µg/mL WT baseline) - incomplete normalization is a well-documented limitation of standalone in vitro ERT. This plateau (green line in Fig. 1A) is probably biologically driven by three primary factors: (1) Receptor saturation: Ald uptake velocity is capped by the availability of surface CI-M6P receptors. (2) Trafficking bottlenecks: Pre-existing lysosomal storage in knockout cells physically disrupts endo-lysosomal fusion, delaying the transit of exogenous enzyme to target sites. (3) Substrate accessibility: Dense, long-standing sGAG aggregates are inherently resistant to rapid enzymatic clearance compared to soluble substrates. Importantly, as the reviewer suggests, this incomplete clearance by ERT monotherapy forms the exact biological rationale for our study. It highlights why exogenous enzymes alone are often insufficient, and demonstrates the advantage of adjunct autolysosomal enhancers such as GHF201 to clear intractable storage pools and approach baseline normalization. Subsequently, we accept the reviewer suggestion and added a combined Ald (5 ug/mL)+GHF201 (150 uM) condition in Fig. 1A. Indeed, this combined treatment attained sGAG levels of 4.15 ug/mL.

We wanted to stress that in our original submission, our exploration of the combination was intentionally focused on upstream lysosomal activation - specifically cathepsin activity, where we were happy to observe clear therapeutic additivity. Because our primary objective for this initial manuscript was to thoroughly characterize the standalone baseline of GHF201, we did not initially extend this combination inquiry to downstream substrate, or sGAG, clearance. However, as said, the reviewer is entirely correct that demonstrating a corresponding further reduction in sGAG levels by the combo treatment provides a definitive, translationally relevant link to patient benefit.

The CNS results were perhaps the most exciting in the manuscript and the most relevant to the field given the lack of current drugs that successfully cross the BBB. Figure 6 shows clear normalisation of neuroinflammation

which could be a major advancement to treating neurological forms of the disease, but very little is mentioned in either the results or discussion. I think these results need to be highlighted more. This is great work that is currently getting lost in the other data.

Response: Thank you for this important comment. We modified relevant text on the CNS effect of GHF-201 in the Results (p. 6, l. 31-43; p. 7, l. 1-9 and Discussion (p. 10, l. 33-46; p. 11, 1. 1-10) sections to better emphasize this translational impact of our study.

General comments:
The figures are very busy making them difficult to look at and interpret. I worry that important information is getting lost with so many panels per figure that don't necessarily go together, e.g. the pharmacological data and behavioural studies in Figure 2. I think it would be beneficial to keep and enhance the most important panels and put the other figures into the supplementary material. This would also simplify the Figure legends which are currently very long and - in many cases - give more information than what is provided in the text.

Response: Thank you for this comment. The reason we included our pharmacokinetics data in Fig. 2 was that, as the reviewer commented above, it was important for us to demonstrate that GHF-201 is biodistributed to the tissues where it exerts a significant effect. For instance, in Fig. 2 which demonstrates the behavioral effects of GHF-201, it was important for us to show that the compound can cross the BBB and be biodistributed to the CNS. Given the importance of these pharmacokinetic data, we did not want to include them in the supplement and, on the other hand, they could also not stand alone as a single figure, and also did not belong in any other figure. However, we accept this comment and have now moved our pharmacokinetics results to a new supplementary figure (Fig. S1).

We also moved Fig. 4A (NfL-LC) and Fig. 4I (urine GAG) to a new supplementary figure (Fig. S12) since these are not metabolomic results, as opposed to the rest of the results in Fig. 4. We hope that these changes adequately address the reviewer's criticism and make our figures more focused and easier to follow.

It is referred to as GHF-201 in the text, but GHF201 in most of the figures and the title. It would be good to be consistent.

Response: Thank you for noticing. We changed all to GHF201.

Glycosaminoglycans is abbreviated to GAGs, however GAG and GAGs are interchanged throughout.

Response: Thank you for noticing. We changed all to GAGs, except when "GAG" is used as an adjective (*e.g.,* "GAG-mediated…"), where it should be used in the singular form for grammatical reasons.

Specific comments/suggestions for the manuscript:
Introduction:
Line 3: define the abbreviation CNS in the first instance.

Response: Corrected.

Line 13: It may be worth starting a new paragraph for the pathological features. I also suggest moving the incidence sentence to the start of the paragraph which may flow better than having it between the peripheral and CNS pathology.

Response: Thank you. We accept your suggestion. We started a new paragraph describing the pathological features and moved the epidemiological part to the beginning of this paragraph.

Row 14: Consider changing to "prominent peripheral features."

Response: Changed. Other text in this paragraph was slightly modified as a result of moving the incidence sentence to the beginning of the paragraph.

Row 15: Consider changing "milder" to attenuated, as per current accepted nomenclature.

Response: Changed.

Reference 16 is incorrect.

Response: Thank you for noticing. This reference was removed.

At the start of the third paragraph (line 24) the abbreviation SoC is not defined.

Response: Corrected.

Line 27: Should this be hematopoietic stem cell transplant (HSCT)? Reference should come after this sentence.

Response: Thank you for the comment. We added Aldehoven *et al* (2015) *Blood* 125: 2164 (PMID: 25624320) as a MPS-I HSCT reference.

Results:
GHF-201 and ERT reduce GAG levels in MEF
I did find this section a little hard to follow, the Figure legend actually explains better than the text. I am not sure I understand the rationale for the addition of 150 µM GHF-201 given GAG concentrations were similar by 50 µM and stabilised. Addition of the recombinant enzyme did not normalise GAG concentrations, which should be noted.

Response: We thank the reviewer for this insightful comment and for pointing out the need for greater clarity in this section. We agree that the original text did not adequately convey the *a priori* experimental design, and we have thoroughly revised the paragraph to clarify our rationale. To clarify, the dosing matrix for both the wild-type (*Idua*$^{+/+}$) and mutant (*Idua*$^{-/-}$) MEFs was established *a priori* based on our previous characterization of GHF201, which indicated that maximal therapeutic effect typically peaks near 50 µM [(Kakhlon *et al.* (2021) *EMBO Mol Med* 13:e14554 (PMID: 34486811); Solmesky *et al.* (2017) *Biochem J* 474:3403 (PMID: 28827282) (there GHF201 is called 144DG11); Mishra *et al.* (2024) *Biomolecules* 14:893 (PMID: 39199279)]. Therefore, the 100 µM and 150 µM concentrations were intentionally included in the *Idua*$^{-/-}$ cohort as saturating, supra-maximal doses (no cell toxicity, in different cell types, has been observed using these doses, unpublished results). While the post-hoc data indeed demonstrate that sGAG reduction stabilizes and reaches an asymptotic plateau by 50 µM, the inclusion of the 100 µM and 150 µM doses was mathematically vital to confirm that the absolute ceiling of GHF201's efficacy had been reached. This ensured that our subsequent benchmarks against recombinant ERT (supplemented Aldurazyme) were comparing the absolute maximal therapeutic potential of both interventions. We have modified the text in the revised manuscript (p. 3, l. 10-23) to describe this rationale.

We also added a biochemical explanation (please also see our first response) on the limited capacity of Ald and thus its inability to normalize GAG levels (p. 3, l. 23-28).

GHF-201 increases cathepsin activity additively with ERT in Idua-/- mouse fibroblasts.
Typo on line 5 - caused by GHF-201...

Response: Corrected.

GHF-201 improves behavioural and motor deficits in Idua-/- mice
I think the authors need to be careful with their interpretation of the behavioural studies. Only the open field showed a true sex difference, with males not having any improvement with treatment. Grip strength in affected

male mice was not decreased compared to WT; this is not a poor response to treatment, but a lack of deficit to begin with and should be noted. In the Water Cross maze, the treated mice showed a similar escape latency to WT for both sexes. This is not reflected in the text.

Response: We entirely agree with the reviewer's nuanced perspective on these behavioral data. We highly appreciate this constructive critique, which has helped us significantly refine the interpretation and clarity of our behavioral narratives. We have addressed each of the three behavioral assays below and corrected the manuscript accordingly (p. 4, l. 6-43): Open Field (True sex difference): We agree that the open field assay stands out as the primary indicator of a true, treatment-resistant sex dimorphism in the male cohort. We have maintained this explicit interpretation in the text. Grip Strength (Lack of baseline deficit): The reviewer is entirely correct. The lack of a treatment response in the male grip-strength assay was not due to therapeutic failure, but rather due to the complete absence of a baseline deficit to begin with. We have completely rewritten this section in both the main text and the supplement (p. 2, l. 19-30; p. 3, l. 1-10) to ensure we do not mischaracterize this as a "poor response." We now explicitly note that male mutant mice maintain their raw grip-strength baseline equivalent to wild-type controls, likely due to a baseline musculoskeletal reserve characteristic of male rodents. Water Maze / Escape Latency (Both sexes reaching WT levels): We appreciate the reviewer pointing out the need for absolute clarity regarding the spatial learning data. Our intended takeaway message matches the reviewer's observation: GHF201 successfully restored spatial learning capacity in both male and female *Idua*$^{-/-}$ mice to levels indistinguishable from their respective WT controls. While we did state this in the original text, we realize that our phrasing might have been buried and lacked the clarity required for such an important finding. We have thoroughly revised this paragraph to make this sex-independent efficacy clearer.

Discussion:
Non-reducing end iduronic acid is not removed in MPS I, that is where the deficiency lies. Please correct in line 5 and 8.

Response: We thank the reviewer for pointing out this oversight. We have revised the text to accurately reflect that the pathology actually stems from the failure to remove these terminal residues (p. 8, l. 13-26).

There needs to be more relation back to the literature. For example, the cascade of secondary cellular pathology is widely documented and should be referenced. I think it would also be good to compare/contrast the results in this manuscript back to those for other types of treatment such as ERT and gene therapy.

Response: We thank the reviewer for this constructive feedback. We completely agree that the downstream cascade of secondary cellular pathology is an essential component of MPS-I pathogenesis. In our original draft, we conceptually highlighted these mechanisms (including mTOR dysregulation, aberrant mitophagy, and lysosomal rupture in the first paragraph of the Discussion, as well as TLR4-mediated inflammation in the third paragraph of the Discussion). To fully address the reviewer's request, we have now explicitly expanded this section in the first paragraph of the revised Discussion to substantiate these statements with key references documenting these secondary cascades. Furthermore, we have expanded our comparative framework in response to the reviewer's second comment (seventh paragraph of the Discussion) to explicitly compare and contrast the capabilities of our small-molecule platform against both standard ERT and emerging Gene Therapy (GT) modalities, highlighting GHF-201's non-invasive blood-brain barrier penetration.

On the third page of the discussion, the abbreviation BBB has not been defined previously.

Response: Corrected.

Methods:
Were the mice embryos used in the MEFs from the same colony described in the "Animals and in vivo tests"?

Response: No, the MEFs were derived from a separate, dedicated breeding cohort established specifically for embryo harvesting. Importantly, this separate cohort shared the identical genetic background (*Idua*$^{-/-}$ and *Idua*$^{+/+}$ wild-type littermates) as the animals utilized in the in vivo studies described in the manuscript. All breeding and harvesting procedures for this cohort were fully reviewed and approved by our IACUC (approval number 17201).

Mouse plasma metabolomics
There is no information on how the plasma samples were extracted. This needs to be included or referenced.

Response: We thank the reviewer for bringing to our attention the omission of the plasma extraction method, which is now added in the Methods section under "Mouse Plasma Metabolomics."

I'm not sure I understand the LC gradient. It started at 80% B and ramped to 20%B at 15 min? Then what? Was it held there or did it go straight back to 80% B for equilibration until 26 min? Please clarify.

Response: We thank the reviewer for highlighting this potential ambiguity. Following the step to 20% B at 17.0 min, the mobile phase was immediately returned to 80% B at 17.1 min and maintained until 26.0 min for column re-equilibration. The Liquid Chromatography gradient description under "Mouse Plasma Metabolomics" has been clarified in the revised manuscript.

"Samples were normalized to the HC group..." I am assuming that HC is healthy control group? How was this normalisation done? Comparison of the area under the curve? Using an internal standard? More information is needed for others to be able to follow.

Response: We thank the reviewer for the opportunity to clarify this step. The acronym HC denotes the Healthy Control (*Idua*$^{+/+}$) group, which served as the baseline reference for relative group comparisons (such as calculating relative fold changes and volcano plot distributions). To ensure transparency and reproducibility, the individual metabolite peak areas (areas under the curve) were uploaded to MetaboAnalyst 6.0 and pre-processed based on the specific requirements of each downstream statistical application:

Univariate Analysis (Volcano Plots / Fold Changes): Because a uniform volume of plasma (20 µL) was processed for every sample at the bench, software-based row-wise sample normalization was not applied. To satisfy the variance assumptions required for parametric statistical analysis (t-tests for selecting the metabolites shown in the heatmap), the peak area data were subjected exclusively to Log transformation (base 10), with data scaling set to "None."

Multivariate Analysis: For multi-variable modeling, the log-transformed peak areas were further subjected to auto scaling (mean-centering and dividing by the standard deviation of each variable) to ensure that high-abundance metabolites did not disproportionately dominate the models. We have updated the text in the "Mouse Plasma Metabolomics" section to explicitly delineate these exact software settings and pre-processing pathways.

Statistics
While I appreciate that small sample sizes do reduce the power of statistics, I do not believe increasing the p value threshold above 0.05 is appropriate. To have this as a 'sliding scale' feels like moving the target to fit the hypothesis, which is not scientifically sound. There is no real rationale for the changes within the manuscript and I would think there would be a need for orthogonal validation that the reported significant changes are not false positives due to the high threshold. It is perfectly acceptable to talk about trends that do not reach significance (especially when they are 0.06 or 0.07) but they are not significantly different, and this should be noted along with the limitation of sample size.

Response: We completely agree with the reviewer that maintaining scientific rigor is paramount and that a 'sliding scale' for statistical significance is inappropriate. We appreciate the constructive suggestion to handle these values as

descriptive trends rather than definitive assertions of significance. Accordingly, we have thoroughly revised the manuscript to explicitly denote all differences where $0.05 < p < 0.10$ as trends, while clearly stating the sample size limitations where appropriate. Regarding the reviewer's point about orthogonal validation and the risk of false positives: by reclassifying these metrics as biological trends rather than significant statistical discoveries, we have mitigated the risk of claiming false-positive significance. Furthermore, we observed that these specific trends do not stand in isolation; they consistently align with the broader, highly significant ($p < 0.05$) downstream phenotypic rescues and biomarker corrections observed across our systemic and CNS datasets. To provide context for why we chose to retain and discuss these trends rather than omitting them entirely, our rationale was guided by modern statistical recommendations—including the American Statistical Association (ASA) Statement on Statistical Significance and P-Values (https://www.tandfonline.com/doi/full/10.1080/00031305.2016.1154108#d1e385). In **exploratory** preclinical models with logistically constrained sample sizes, avoiding rigid binary thresholds helps prevent severe Type II errors (dismissing genuine, biologically relevant therapeutic signals). We believe this revised language maintains adherence to the conventional $p < 0.05$ binary threshold for significance, while transparently presenting these parallel biological trends for the reader's consideration. This approach also aligns with the following high impact references: Halsey, L. G. (2019). The reign of the p-value is over: what alternative analyses could we employ to fill the power vacuum? Biol Lett, 15(5), 20190174; Lazzeroni, L. C., et al. (2016). Solutions for quantifying P-value uncertainty and replication power. Nature Methods, 13(2), 107–108; Halsey, et al. (2015) The fickle P value generates irreproducible results. Nature Methods 12:3 12, 179–185; Krzywinski, M. & Altman, N. (2013) Power and sample size. Nat Methods 10, 1139–1140.

Figure 2:
There are abbreviations in the Figure legend that have not been defined in full.

Response: We thank the reviewer for pointing out this omission. We have thoroughly revised the legend for Figure 2 to explicitly define all abbreviations in full. The abbreviated terms we were able to find were Veh (Vehicle), and ANOVA (Analysis of Variance). These abbreviation are now defined at the end of this figure legend.

The y-axes for the open field graphs would be better in cm.

Response: Corrected.

The examples of tracking and heat maps would be better in the supplementary material.

Response: Thank you. This inset was moved to Fig S2C and the text was updated accordingly.

The statistics in the Figure legend for the Water Cross Maze are unclear and not indicated on the Figure, and the wording is a little confusing, e.g. the sentence below.
"...reduction in escape latency over time, or spatial learning, is reduced in Idua-/-mice as compared to Idua+/+ ($p<0.03$) and improved by GHF-201..." Please consider changing the wording for clarity.

Response: We thank the reviewer for pointing out this possibly confusing phrasing. We agree that the expression "reduction... is reduced" could have been linguistically circular and that the notation $p < 0.50$ was unconventional for denoting non-significance. To resolve this, we have reworded the legend for clarity, adding color-based group designations. We have explicitly stated the statistical test used (Repeated Measures ANOVA), corrected the non-significance notation to $p > 0.05$, and streamlined the text to standard behavioral nomenclature.

Figure 3:
Label the graphs in panel B.
Abbreviations in the Legend that are not defined.

Response: We thank the reviewer for pointing out the omission of the labels on the on-gel quantifications plots. We have now added those labels ("in vivo" and "in vitro"). We also thank the reviewer for pointing out the missing definitions in the figure legend. We have updated the legend for Fig 3 to write out all abbreviations.

Figure 4:
There is no key for panels G and H.

Response: We thank the reviewer for pointing out this omission. We agree that the graphical elements of the pathway topology plots (from MetaboAnalyst 6.0) require explicit definitions within a standalone legend. We have updated the figure legend text for former panels **G** and **H** (now changed to **F** and **G**, following a previous comment) to provide a clear visual key for the plot graphics. The legend now explicitly clarifies that the circle sizes represent the calculated pathway impact scores, while the color gradient indicates the level of statistical significance (-$\log_{10}$(p-value)).

I would suggest removing the ROC curve, as it does not add anything more than the PLS plot.

Response: We appreciate the reviewer's suggestion to streamline the figures and avoid redundancy. While we agree that both plots reflect distinct profiles between the *Idua*$^{-/-}$ and *Idua*$^{+/+}$ cohorts, they provide fundamentally different and complementary statistical insights, which are standard in metabolomics workflows: PLS-DA (Panel B) is an exploratory, supervised dimensionality-reduction tool designed to demonstrate global variance and cluster separation across the entire detected metabolome. It does not evaluate predictive classification accuracy. The ROC Curve (Panel C), on the other hand, is a predictive validation tool. It moves beyond global clustering to test the diagnostic power of a minimized, concrete biomarker panel (specifically, 15 metabolites), establishing a highly robust classification probability (AUC = 0.94). It explicitly answers how many and which specific features are required to reliably distinguish the phenotypes. Retaining this analysis also ensures methodological consistency with our other experimental figure (Fig 8G), where ROC analyses are similarly leveraged to quantify predictive performance. However, we fully respect the reviewer's goal of optimizing the manuscript's flow. To make this distinction completely transparent, we have revised the figure legend to clarify the predictive nature of the ROC panel. Alternatively, if the reviewer still feels it is overly dense for the main text, we are open to moving the ROC curve panel to the Supplementary Information.

Panel D says Empty ND (please define in the legend) and GHF 201, however in the legend it only mentions WT and affected mice.

Response: We thank the reviewer for pointing out this error. The comparisons in panels D and E are of course between ND-GHF201 treated and empty-ND treated *Idua*$^{-/-}$ mice. Empty-ND is simply an unloaded non-aqueous oral solution nanodroplet, as opposed to ND-GHF201 which was loaded with the GHF201 payload. This has now been corrected in the manuscript.

Figure 8:
No title on the x-axis on panel B.

Response: We thank the reviewer for pointing out the apparent omission of the x-axis in panel B. It was simply covered by panel E below it. It has now been corrected.

Label panel J. Are these two different brains? Two different sections?

Response: Thank you for this important comment. These are representative sections from two different brains – one derived from an *Idua*$^{-/-}$ mouse treated with a control unloaded formulation and the other derived from an *Idua*$^{-/-}$ mouse treated with ND-GHF201. We have now labeled the sections and also edited the figure legend text accordingly.

Supplementary material:
To be clear, rapamycin and 3-MA were administered to affected littermates that had not received treatment in the preceding time?

Response: Yes. That is correct. Rapamycin and 3-MA were indeed administered (for only two weeks due to known toxicity) as standalone drugs in littermates which did not receive GHF201 at all. Thank you for highlighting this detail. We have now added this clarification in both the Results and Supplementary sections.

It is stated that grip strength was decreased by 3-MA, however this is the same as what is seen in affected mice, so is it not that 3-MA had no effect?

Response: We entirely agree with the reviewer's assessment. Indeed, our data indicates that 3-MA administration had no additional statistical effect on grip strength beyond the deficit already caused by the disease state itself. Our original statement was intended to convey this exact lack of effect, noting that the 3-MA group was not significantly different from the untreated *Idua*$^{-/-}$ group. However, we recognize that the original phrasing may have been ambiguous or easily misread. To ensure clarity, we have revised this sentence to explicitly state that grip strength reduction exerted by 3-MA on *Idua*$^{-/-}$ mice was not statistically significant (supplementary text, p. 2, l. 19-30).

How is open field influenced by grip strength? (paragraph 3)

Response: We thank the reviewer for highlighting the intrinsic relationship between these two behavioral assays. Grip strength serves as a direct readout of skeletal muscle force and peripheral neuromuscular integrity. Conversely, the open field test integrates both gross locomotor output and central open field exploratory drive (anxiety-like behavior). Peripheral muscle weakness and decreased grip strength act as a primary physical constraint on an animal's overall motor capacity. If a mouse suffers from compromised musculoskeletal strength, its physical capability to traverse the open field arena is inherently restricted, leading to a secondary reduction in total moving distance. To confirm that this decreased moving distance was indeed driven by physical motor limitations rather than altered emotionality or lack of exploratory drive, we analyzed the classic anxiety-related parameters within the open field assay (e.g., time spent in the center zone vs. the periphery). We observed no significant differences in anxiety-like behavior across the experimental groups (Fig. S5). This thus rules out anxiety as a confounding factor and confirms that the reduced open field activity is a direct consequence of compromised motor capacity. By presenting both readouts, we demonstrate that GHF201 treatment concurrently ameliorates both the underlying physical muscle force (grip strength) and the integrated, gross functional output (open field distance) without altering the emotional baseline of the animals.

Reviewer #2 (Comments to the Authors (Required)):

In this paper, Kumar et al have described the usage of GHF-201, an established lysosomal activator drug for the potential treatment of MPS-1 mouse model. They utilize cell culture and in-vivo tissue studies to study the efficacy of the drug. While overall the results are encouraging, there are sections, where the interpretation and a follow up experiment is not done to reach the stated conclusion. Some results presented in the paper is also not interpreted well enough to justify what was observed.
1. One of the main conclusions of the paper as mentioned in the discussion section is to use this drug alongside already existing enzyme replacement therapy. The idea primarily comes from the result presented in Fig 1B. In this result, the focus is made onto the activity state of cathepsin B, which seems to be improved by the treatment of GHF-201, however looking at the calcein staining, seems the cell viability is severely compromised in the combo treatment. The author shows a graph (Fig. 1C) that stand alone treatment with GHF-201 does not impact cell viability. However, no data was presented to show viability of the cells in the combo treatment. And the Calcein data raises a doubt whether the combo treatment affects cell survival significantly even if it is able to induce cathepsin B activity. In the absence of such data, the conclusion and using the drug in a combo therapy claim cannot be made.

Response: We thank the reviewer for raising this point, allowing us to clarify the cell viability data and the microscopy images of combination-treated cells. Following this reviewer's comment, cell viability for the combination treatment ("Combo", Aldurazyme + GHF201) has also been evaluated via MTT assay, complementing the monotherapy dose-response curve shown in current Figure 1D, and is now presented in the new Figure 1E. As demonstrated in Figure 1E, combination treatment in both *Idua*$^{+/+}$ and *Idua*$^{-/-}$ MEFs maintains cell viability at levels comparable to untreated controls (~95–100%), confirming that the combination therapy does not induce cytotoxicity. The fluorescent micrographs in Figure 1B were acquired using an Operetta High-Content Analysis (HCA) imaging system, which quantifies signals across multiple automated fields per well (as reflected in current Figure 1C). The images displayed in Figure 1B are qualitative, hand-picked representative fields intended primarily to illustrate active cathepsin labeling (GB123 probe, red channel) alongside cell morphology/staining (Calcein, yellow channel). Because these panels represent single localized fields of view, natural variations in cell distribution across the well can yield localized differences in apparent cell density that do not reflect overall viability. On the other hand, robust automated quantification across numerous fields per well, as reflected in current Figure 1C for GB123 intensity and orthogonally validated by the plate-wide MTT assay in Figure 1E, confirms that cell viability and total cell mass are fully preserved under combination treatment. We have revised the main text in the Results section to explicitly reference Figure 1E when discussing the safety profile of the combination treatment alongside Figure 1D. We have also updated Figure 1's Legend to clearly state that Figure 1E demonstrates the viability of the combination treatment. Please note that we have also replaced the representative field for the *Idua*$^{-/-}$ Combo panel in Figure 1B with a field of view that more accurately reflects the typical, uniform cell density observed across the wells. Additionally, we noticed that the Calcein micrograph for the *Idua*$^{-/-}$/Aldurazyme group was inadvertently duplicated in the *Idua*$^{-/-}$/GHF201 panel. We have now updated Figure 1B with the correct, representative image for the *Idua*$^{-/-}$/GHF201 group.

2. In the same figure a panel (Fig 1A) shows the GAG concentration decrease in knockout and WT cells with the ERT and new drug. What about the combo? How is the GAG levels respond to the combo treatment? Secondly, looking at this result, seems beyond 50 uM dose there is not really any effect of the drug on the GAG reduction. Therefore, the conclusion that GAG reduction happens in a dose dependent manner should emphasize this observation and its potential translational implication/drawbacks

Response: These points were also made by the first reviewer. Therefore, we have copied here our relevant responses to the first reviewer comments.

Here is our response regarding the addition of the Combo condition to the sGAG experiment (from our response to the first comment of the first reviewer):

"We also thank the reviewer for the justified comments and observations on our MEFs studies. While Aldurazyme (Ald) significantly reduced sGAGs - clearing over 50% of the pathological excess (from 5.2 down to 4.5 µg/mL, against a 3.85 µg/mL WT baseline) - incomplete normalization is a well-documented limitation of standalone in vitro ERT. This plateau (green line in Fig. 1A) is probably biologically driven by three primary factors: (1) Receptor saturation: Ald uptake velocity is capped by the availability of surface CI-M6P receptors. (2) Trafficking bottlenecks: Pre-existing lysosomal storage in knockout cells physically disrupts endo-lysosomal fusion, delaying the transit of exogenous enzyme to target sites. (3) Substrate accessibility: Dense, long-standing sGAG aggregates are inherently resistant to rapid enzymatic clearance compared to soluble substrates. Crucially, as the reviewer suggests, this incomplete clearance by ERT monotherapy forms the exact biological rationale for our study. It highlights why exogenous enzymes alone are often insufficient, and demonstrates the advantage of adjunct autolysosomal enhancers such as GHF201 to clear intractable storage pools and approach baseline normalization. Subsequently, we accept the reviewer suggestion and added a combined Ald (5 ug/mL)+GHF201 (150 uM) condition in Fig. 1A. Indeed, this combined treatment attained sGAG levels of 4.15 ug/mL.

We wanted to stress that in our original submission, our exploration of the combination was intentionally focused on upstream lysosomal activation - specifically cathepsin activity, where we were happy to observe clear therapeutic

synergy. Because our primary objective for this initial manuscript was to thoroughly characterize the standalone baseline of GHF201, we did not initially extend this combination inquiry to downstream substrate, or sGAG, clearance. However, as said, the reviewer is entirely correct that demonstrating a corresponding further reduction in sGAG levels by the combo treatment provides a definitive, translationally relevant link to patient benefit."

Here is our response to the reviewer's comment on the oversaturation of the GHF201 effect (from our response to the 13th comment made by the first reviewer):

"**Response:** We thank the reviewer for this insightful comment and for pointing out the need for greater clarity in this section. We agree that the original text did not adequately convey the *a priori* experimental design, and we have thoroughly revised the paragraph to clarify our rationale. To clarify, the dosing matrix for both the wild-type (*Idua*$^{+/+}$) and mutant (*Idua*$^{-/-}$) MEFs was established *a priori* based on our previous characterization of GHF201, which indicated that maximal therapeutic effect typically peaks near 50 µM [(Kakhlon *et al.* (2021) *EMBO Mol Med* 13:e14554 (PMID: 34486811); Solmesky *et al.* (2017) *Biochem J* 474:3403 (PMID: 28827282) (there GHF201 is called 144DG11); Mishra *et al.* (2024) *Biomolecules* 14:893 (PMID: 39199279)]. Therefore, the 100 µM and 150 µM concentrations were intentionally included in the *Idua*$^{-/-}$ cohort as saturating, supra-maximal doses (no cell toxicity, in different cell types, has been observed using these doses, unpublished results). While the post-hoc data indeed demonstrate that sGAG reduction stabilizes and reaches an asymptotic plateau by 50 µM, the inclusion of the 100 µM and 150 µM doses was mathematically vital to confirm that the absolute ceiling of GHF201's efficacy had been reached. This ensured that our subsequent benchmarks against recombinant ERT (supplemented Aldurazyme) were comparing the absolute maximal therapeutic potential of both interventions. We have modified the text in the revised manuscript to describe this rationale.

We also added a biochemical explanation (please also see our first response) on the limited capacity of Ald and thus its inability to normalize GAG levels."

3. Minor point: Fig 2 panel C, place the genotypes on the open filed pictures for a quick and better visualization by the reader

Response: Corrected. Now this open field images are in new Fig S3C, as per the request of the first reviewer.

4. Fig 3, panel B - The results shown somehow does not correlate with the quantification shown and hence the conclusion presented in the result section ("IVIS imaging and on gel studies demonstrate attenuation of tissue cathepsins by GHF-201"). The on-gel in-vivo result clearly demonstrates that Idua-/-/GHF-201 lane barely has any detectable cathepsin, compared to WT. However, the quantification shows that this is significantly indistinguishable from WT. How was the quantification done to get this opposite conclusion? Where is the loading control for this assay?

Response: We thank the reviewer for this insightful observation and for giving us the opportunity to clarify both our densitometry methodology and experimental design. Densitometric quantification (performed using ImageJ) was executed by integrating the total signal intensity across the entire lane rather than measuring solely the primary ~30 kDa mature Cathepsin B band. Please note that the active Cathepsin B signal in WT tissue is focused almost entirely into a single, highly concentrated, sharp band at 30 kDa. While this primary 30 kDa mature Cathepsin B band is markedly suppressed in the *Idua*$^{-/-}$/GHF201 group, the active probe signal in this lane is redistributed across a broader, diffuse multi-band pattern between 30-37 kDa. Thus, the cumulative signal in *Idua*$^{-/-}$/GHF201 (75%) is quantitatively comparable to the sharp, concentrated signal of WT (80%). We wish to clarify that the active probe used in this on-gel assay selectively labels catalytically active cathepsins. Pro-cathepsin B is enzymatically inactive (and also migrates above 40 kDa), meaning it is not detected by the GB123 probe. The additional active bands migrating between 30 and 37 kDa probably represent hyperglycosylated active Cathepsin B species, a hallmark of impaired lysosomal processing in *Idua*$^{-/-}$

tissue. Importantly, directly referring to the reviewer's query on the loading control, we also wish to clarify, that this is an on-gel activity-based probe (ABP) assay measuring active catalytic sites, rather than a standard Western blot for total protein expression. Because housekeeping proteins (*e.g.*, β-actin or GAPDH) cannot be probed simultaneously on a fluorescent activity gel, equal loading was controlled prior to gel loading by measuring total protein concentration in all tissue homogenates using the BCA protein quantification assay and loading identical total protein amounts per lane. These details are now added to the figure legend.

5. The mobility shift of cathepsin that is shown in this on-gel assay is contributed to the hyperglycosylation of cathepsin B. Interestingly the WT does not have this. Was hyperglycosylation confirmed using some sort of quick biochemical assays such as EndoH or PNGase F treatment? The authors explain this as a result of lysosomal dysfunction. As the whole point of the GHF-201 treatment is to improve lysosomal function, it is surprising that the treatment did not cause any change (even if not significant but a trend towards lowering this hyperglycosylation of cathepsinB). The author should explain this drawback in the discussion section.

Response: We thank the reviewer for this thoughtful comment regarding the altered mobility of active Cathepsin B in *Idua*$^{-/-}$ tissue and its response to GHF201 treatment. Enzymatic deglycosylation (*e.g.,* by EndoH or PNGase F treatment) was not incorporated into our initial experimental workflow, as the primary objective of this experiment was to profile overall active catalytic cathepsin pools using activity-based probes (ABPs), rather than to phenotype post-translational glycosylation profiles. Unfortunately, tissue lysates from these specific mouse cohorts have been fully exhausted, precluding post-hoc deglycosylation assays. However, altered N-glycosylation and higher molecular weight mobility shifts of active cathepsins in models of lysosomal storage disorders, resulting from impaired lysosomal maturation and lysosomal enzyme recycling, are well-documented in other LSDs, such as Mannosidosis (Damme *et al.* DOI: 10.1128/MCB.01143-09) and Niemann-Pick type C diseases (Dixit et al.,DOI: 10.1371/journal.pone.0023677). These citations are now added to the manuscript.

To directly evaluate whether GHF201 attenuates the higher-molecular-weight, probably hyperglycosylated, active Cathepsin B species (>30 kDa), we performed targeted densitometric quantification focused specifically on this upper band/smear region (>30 kDa) across all independent biological replicates (N=3): Our results are now added as a new panel to Figure 3B and show that untreated *Idua*$^{-/-}$ liver tissue exhibits a pronounced accumulation of the active >30 kDa hyperglycosylated species compared to WT controls. Specific quantification of this >30 kDa region demonstrates that GHF201 treatment significantly reduces (or markedly decreases) the abundance of these aberrant hyperglycosylated active Cathepsin B species in both in vivo and in vitro conditions. Therefore, rather than representing a drawback, these quantified data demonstrate that GHF201 not only reduces total pathologically elevated cathepsin activity but also specifically attenuates the accumulation of altered, hyperglycosylated active cathepsin forms stemming from lysosomal dysfunction.

6. Fig 4. The metabolomics study clearly shows that the treatment has some sort of effect that is different than WT metabolism. Interestingly the shift is towards more catabolism as seen in other diseases when treated with GHF-201. This implies this maybe a general effect of GHF-201 rather than specific for MPS-I during any pathological conditions. Also, too much catabolism could be detrimental. How in other diseases this shift helped in restoring cellular homeostasis and not any detrimental effect needs to be pointed out in the discussion.

Response: We thank the reviewer for this perceptive observation regarding the metabolomic signature induced by GHF201. The reviewer correctly highlights two central aspects of GHF201's activity: (1) Broad-spectrum mechanism: GHF201 functions as a generalized enhancer of catabolic flux and metabolic efficiency across multiple pathological conditions, rather than acting through a single, narrow disease-specific target. (2) Homeostatic benefit: This catabolic shift is therapeutically restorative and self-limiting, rather than causing detrimental or toxic hyper-catabolism. The therapeutic benefit of GHF201 relies on controlled, homeostatic catabolic activation rather than unchecked or

detrimental hyper-catabolism. We agree that expanding on this mechanism in the Discussion section significantly strengthens the manuscript. Indeed, as suggested by the reviewer, we have now integrated our extensive cross-disease findings, which explain how this catabolic shift successfully restores cellular homeostasis without causing detrimental metabolic toxicity (p. 10, l. 22-32).

7. Minor point: Bone dysplasia pictures Fig 5A is not convincing to look at the effect of GHF-201. The knockout and the treatment bones look very similar. Is it possible to clearly label where the measurement was taken to produce the graph next to it? Same to do for the supplementary figures for the treatment with Rapamycin and 3MA.

Response: We thank the reviewer for this helpful suggestion. To make the morphological differences and relative proportions clear and intuitive to the reader, we have added red reference lines demarcating both shaft length (vertical projection) and joint width (horizontal) across all micro-CT render images in Figure 5A and Supplementary Figure S8. The figure legends have been updated to describe these visual indicators. We wish to clarify a technical aspect regarding the quantitative data: while the red lines in the 2D figure panels represent linear 2D projections provided for straightforward visual orientation, the quantitative shaft length measurements reported in the manuscript were calculated using Imalytics software along the full 3D anatomical/curved trajectory of the bone shaft. This 3D tracing accurately accounts for phenotypic differences in bone curvature and bowing across groups. With these visual reference lines in place, the phenotypic effect of GHF201 is clearly illustrated: while untreated *Idua*$^{-/-}$ bones exhibit a distinctly stunted, bowed shaft paired with a widened, dysplastic joint (yielding a reduced length-to-width ratio), GHF201 treatment visibly restores shaft extension relative to joint width, shifting overall bone proportions back toward *Idua*$^{+/+}$ wild-type morphology.

8. Striking results is the reduction of vacuolar structures as shown by EM in the liver and Kupffer cells. Not so much went into the discussion for these results. Please explain in the discussion how do you think GHF-201 may contribute to this phenotype and how this may correlate with the pathogenesis reduction in MPS-I. What type of vacuoles they are? Lysosome vs other vacuoles (i.e. double vs single membrane bound vacuoles as can be easily seen in EM)

Response: We thank the reviewer for highlighting this key result and suggesting a deeper discussion of these ultrastructural findings. Morphologically, the vacuolar structures observed in untreated *Idua*$^{-/-}$ hepatocytes and Kupffer cells are single-membrane-bound storage vacuoles representing severely distended lysosomes and autolysosomes (terminal degradative compartments) filled with undigested, electron-lucent GAGs (being water-soluble these GAGs wash out during aqueous fixation), rather than double-membrane-bound nascent autophagosomes. In MPS-I, impaired enzymatic trimming leads to substrate accumulation, lysosomal swelling, and blocked autophagic/lysosomal clearance. Because GHF201 enhances overall lysosomal hydrolytic activity, autolysosomal flux, and acidification, it restores the cell's ability to clear this sequestered GAG cargo. This directly relieves organellar distension, resulting in the marked quantitative reduction of single-membrane storage vacuoles in both parenchymal hepatocytes and immune Kupffer cells. Importantly, we presume that GHF201 clears these storage vacuoles by enhancing intralysosomal enzymatic degradation rather than promoting lysosomal exocytosis or uncoordinated release of undigested substrate into the extracellular space. In Kupffer cells (liver-resident macrophages), degrading GAGs within the organelle prevents them from accumulating in the extracellular matrix where they would otherwise act as TLR4 agonists to trigger chronic cytokine release. Thus, true intralysosomal clearance in these cells directly correlates with the attenuation of hepatic inflammation and overall visceral disease severity. We have expanded the Discussion section (fourth paragraph of the revised Discussion, p. 9, l. 24-34) to explicitly detail the single-membrane lysosomal nature of these storage vacuoles and elaborate on how GHF201-mediated clearance in liver cell types contributes to systemic pathogenesis reduction in MPS-I.

9. Similar to the vacuolar structures in the brain also altered in the brain (Fig. 6) i.e. less vacuolar structures in the brain with the treatment. As this effect is seen in multiple tissues - the relevant biology and an explanation

of the effect of GHF-201 is warranted. Also the arrows are not clear in this figure, make them prominent to see the difference in the vacuoles numbers. Right now the histology pictures do not any difference to the eye, unless we look at the quantification.

Response: We thank the reviewer for this comment and for the opportunity to clarify both the visual annotations and the underlying cross-tissue biological mechanism of GHF201. To make the ultrastructural differences immediately apparent, we have now added prominent red arrows directly pointing to the primary clusters of storage vacuoles in the *Idua*$^{-/-}$ panels of Figure 6D. Treatment with GHF201 induced a striking clearance of these storage compartments. The cytoplasm is cleared of the vast vacuolar clusters, leaving normal cellular architecture and cytoplasmic organelle density that closely mirrors the *Idua*$^{+/+}$ wild-type control. In the Results section (p. 7, l. 1-9), we explicitly detail the nature of these brain vacuoles and their resolution upon treatment: "Compared to *Idua*$^{+/+}$ controls, untreated *Idua*$^{-/-}$ mice manifested extensive cytoplasmic vacuolation. Specifically, microglia displayed large, electron-lucent vacuoles — a classic hallmark of water-soluble GAG accumulation, where the water-soluble GAGs wash out during aqueous fixation (Fig 6D). These distended structures were delineated by a defined limiting membrane, identifying them as severely pathological lysosomes. Remarkably, GHF201 treatment significantly reduced the number and volume of these distended lysosomes. This ultrastructural clearance provides direct visual evidence that GHF201 alleviates lysosomal substrate overload and restores functional organelle morphology within the deep tissues of the brain." In the Discussion section (p. 10, l. 45-46+p. 11, l. 1-10), we connect these ultrastructural observations in the CNS to the drug's cell-autonomous mechanism of action across visceral and neural tissues: "The translational significance of these findings for the lysosomal storage disease field lies in the fact that GHF201-mediated clearance operates across multiple scales—resolving tissue histopathology and organelle ultrastructure, while simultaneously driving cell-autonomous enzymatic activation. At the nanoscale level, our ultrastructural TEM examination (Fig 6D) revealed a striking reduction in the number and volume of distended, electron-lucent lysosomes within microglia, indicating that the drug actively relieves lysosomal storage in situ."

10. The paper title does not fully reflect the content of the paper. The treatment certainly doing something in the knockout mouse and, in some cases, it is not sure whether it is a 'rescue' such as looking at the plasma metabolomics data or glycan data (Fig. 8). These data showed that the treatment cohort is different than the knockout cohort but definitely not as the same as WT cohorts. All could be said some phenotypes were altered by this treatment and in some cases some pathophysiological rescues was observed (bone dysplasia result). The title needs to be changed to emphasize this.

Response: We completely agree with the reviewer's nuanced assessment. To accurately reflect that GHF201 attenuates the disease phenotype across datasets without claiming a full, uniform restoration to wild-type levels, we have updated the title language, replacing "rescue" with "attenuation".

11. Was there any tox study done in the mouse model in the WT mouse to show that it does not have any toxic effect in animal model?

Response: Yes, comprehensive safety and toxicity evaluations of GHF-201 (formerly called 144DG11) have been systematically conducted in wild-type animals as well as across multi-species preclinical toxicological programs prior to its advancement into clinical trials. As originally demonstrated in our foundational study (Kakhlon et al., EMBO Mol Med 2021; 13(10):e14554), GHF-201 was administered to wild-type mouse control cohorts to evaluate systemic tolerability by a comprehensive Irwin test and by tissue histology, and weight monitoring. In WT mice, GHF-201 treatment elicited no observable toxicity, no physiological impairment, and no histopathological or ultrastructural damage in key organs. Beyond wild-type mice, GHF-201 has undergone a formal, comprehensive preclinical safety pharmacology and toxicology package as part of regulatory filings (IND). Acute and repeated-dose toxicological evaluations performed across both rodent (mice, rats) and non-rodent species (dogs, cats) confirmed a wide safety margin without compound-related organ toxicity or adverse safety signals. Reflecting the strong safety profile established in preclinical models,

GHF-201 has successfully progressed into human clinical development, currently being evaluated in a Phase 2/3 adaptive clinical trial in Adult Polyglucosan Body Disease (https://www.neurology.org/doi/abs/10.1212/WNL.0000000000215858), alongside long-term compassionate use programs (exceeding 12 cumulative patient-years of exposure) with no reported drug-related safety issues or toxicities.

August 25, 2026

RE: Life Science Alliance Manuscript #LSA-2026-03750-TR

Prof. Or Kakhlon
Hebrew University of Jerusalem
Neurology
Ein Kerem
Jerusalem 9112001
Israel

Dear Dr. Kakhlon,

Thank you for submitting your revised manuscript entitled "Autolysosomal enhancer GHF201 attenuates pathophysiology in a mucopolysaccharidosis-I mouse model". We apologise for the delay in communicating a decision due to editor availability issues.

Your revised manuscript has been evaluated by both the original reviewers whose comments are appended below. As you will read, both reviewers have commented positively on your revised manuscript. Reviewer 2 has noted some minor but valid suggestions to improve the clarity of the manuscript.

Based on the overall assessment of both reviewers, we would be happy to publish your paper in Life Science Alliance pending resolution of the minor points from Reviewer 1 as well as final revisions necessary to meet our formatting guidelines.

MANUSCRIPT ORGANIZATION AND FORMATTING:

To avoid unnecessary delays in the acceptance and publication of your paper, please read the following information carefully. Full guidelines are available on our Instructions for Authors page, https://www.life-science-alliance.org/authors

Submission of a paper that does not conform to Life Science Alliance guidelines will delay the acceptance of your manuscript.

-Please provide the original uncropped scan for the western blot shown in figure 3B (right panel, "in-vitro"). This image was flagged during our routine image integrity screening. You may upload include the original scan as Supporting Data associated with Figure 3.
-Please include scale bar and size information for images in Figure S11. Please also make the scale bars and size information in all figures more prominent. Kindly indicate scale bar size in the legend for figure 5 and S10.
-Please incorporate all results and methods from the supplementary section ("Supplementary Material file") into the respective sections of the main text (results, materials and methods respectively). Please provide sufficient details on your description of combo therapy, and for spatial profiling of brain N-glycans. LSA guidelines require that the manuscript has sufficient detail so that all experimental procedures can be repeated by others, in conjunction with cited references.
-Kindly include molecular weight markers for blots in Figure 5.
-Please remove legends from the supplementary figures. The legends should appear only in the manuscript file.
-As also indicated by Reviewer 1, please note that Figure S5 is missing.
-Please mark the authors' contribution section correctly - not as acknowledgment, and be sure that all authors are mentioned.
-The contributions selected for the following authors do not qualify them for authorship. Please either update the contributions in our system and in the Author Contributions section of the manuscript, or let us know if the authors need to be removed (and added eventually to the acknowledgment section): Haitham Amal, Hanna Rosenmann, Miguel Weil, Matthew Gentry, and Galia Blum.
-Please consult our manuscript preparation guidelines https://www.life-science-alliance.org/manuscript-prep and make sure your manuscript sections are in the correct order.
-LSA does not permit citation of "data not shown," "manuscript in preparation," "manuscript submitted," "unpublished," etc., in any section of the manuscript. For example in line 435, please provide the evidence or remove the claim on unpublished results for the reversal of N-glycan accumulation by GHF201.
-The "Data Availability" section should be placed after the Materials & Methods section. Please consult our guidelines at https://www.life-science-alliance.org/manuscript-prep#format
-Please add callouts for Figures 7A-B; S1A-B; S2A-B; S3A-C; S4A-B; S6A-B; S7A-B; S9A-B and S10 to your main manuscript text.
-Please note that the ORCID IDs are obligatory for all corresponding authors. The 2ndary CA has received instructions on how to connect their OrciD ID with their account.
-Please add the X and Bluesky handles of your host institute/organization, as well as your own, and/or one of the authors, in our system.

-Please upload a clean manuscript file without the track changes. The version with the track changes should be uploaded with the file designation "Related Manuscript file" .
-Please label the abstract clearly in the manuscript file.
-Please add your main and supplementary figure legends to the main manuscript text after the references section.
-Please be sure that the authorship listing and order is correct.

We encourage our authors to provide original source data, particularly uncropped/-processed electrophoretic blots and spreadsheets for the main figures of the manuscript. If you would like to add source data, we would welcome one PDF/Excel-file per figure for this information. These files will be linked as supplementary "Source Data" files.

It is Life Science Alliance policy that if requested, original data images must be made available to the editors. Failure to provide original images upon request will result in unavoidable delays in publication. Please ensure that you have access to all original data images prior to final submission.

If you are planning a press release on your work, please inform us immediately to allow informing our production team and scheduling a release date.

We welcome submissions of potential cover images for the issue of LSA in which your work would appear. If you have high quality images associated with this work, please feel free to email these, with a caption, to the journal office.

LSA encourages authors to provide a 30-60 second video where the study is briefly explained. These videos will be appear embedded with the manuscript online at Life Science Alliance, and on social media to promote the published paper and authors (for examples, see https://docs.google.com/document/d/1-UWCfbE4pGcDdcgzcmiuJl2XMBJnxKYeqRvLLrLSo8s/edit?usp=sharing). Corresponding or first-authors are welcome to submit the video. Please submit only one video per manuscript. The video can be emailed to contact@life-science-alliance.org

FINAL FILES:

To upload the final version of your manuscript, please log in to your account: https://lsa.msubmit.net/cgi-bin/main.plex
You will be guided to complete the submission of your revised manuscript and to fill in all necessary information. Please get in touch in case you do not know or remember your login name.

The following items are required for acceptance.

-- An editable version of the final text (.DOC or .DOCX) is needed for copyediting (no PDFs).

-- High-resolution figure, supplementary figure and video files uploaded as individual files: See our detailed guidelines for preparing your production-ready images, https://www.life-science-alliance.org/authors

-- Summary blurb (enter in submission system): A short text summarizing in a single sentence the study (max. 200 characters including spaces). This text is used in conjunction with the titles of papers, hence should be informative and complementary to the title. It should describe the context and significance of the findings for a general readership; it should be written in the present tense and refer to the work in the third person. Author names should not be mentioned.

The license to publish form must be signed before your manuscript can be sent to production. A link to the license to publish form will be available to the corresponding author only. Please take a moment to check your funder requirements.

Reviews, decision letters, and point-by-point responses associated with peer-review at Life Science Alliance will be published, alongside the manuscript. If you do want to opt out of having the reviewer reports and your point-by-point responses displayed, please let us know immediately.

Thank you for your attention to these final processing requirements. Please revise and format the manuscript and upload materials as soon as you are able.

Thank you for this interesting contribution to the literature. We look forward to publishing your paper in Life Science Alliance.

Sincerely,

Sarita Hebbar, PhD
Scientific Editor
Life Science Alliance
http://www.lsajournal.org

Reviewer #1 (Comments to the Authors (Required)):

I would like to thank the authors for the hard work that they have put into the manuscript following the previous review. I am satisfied that my concerns have been addressed either by revision or in the author's comments. I have some minor suggestions below.

Line 26: only one s in abbreviation GAGs. Again, on line 28 and line 39.
Line 145: Two full stops.
Line 148: Should this be "...ameliorated muscle dysfunction?
Line 181: You should specify Niemann-Pick Disease type C1 and C2, to differentiate from Niemann-Pick types A and B.
Line 187: Consider breaking this sentence up, it is very long and wordy as is.
Line 398: Bracket before GSD-II
Line 653: Should be under 0.05
Line 677: Check start of line. "GHF201GHF201GHF201"?
Line 711: Remove GHF201 from the start of the line.
There was no Supplementary Figure 5.
Fig. S12: Figure B has stars for levels of significance with no key while Figure A has numerals.

Reviewer #2 (Comments to the Authors (Required)):

I have gone through the response to the reviewer's document and the associated changed manuscript to incorporate those changes. The paper is now suitable for possible acceptance in the journal.

September 16, 2026

RE: Life Science Alliance Manuscript #LSA-2026-03750-TRR

Prof. Or Kakhlon
Hadassah-Hebrew University Medical Center
Neurology
Ein Kerem
Jerusalem 9112001
Israel

Dear Dr. Kakhlon,

Thank you for submitting your Research Article entitled "Autolysosomal enhancer GHF201 attenuates pathophysiology in a mucopolysaccharidosis-I mouse model", and for addressing our formatting requests. It is a pleasure to let you know that your manuscript is now accepted for publication in Life Science Alliance. Congratulations on this interesting work.

The final published version of your manuscript will be deposited by us to PubMed Central upon publication.

Your manuscript will now progress through copyediting and proofing. It is journal policy that authors provide original data upon request.

Your article will publish open access upon publication under a CC-BY license.

Reviews, decision letters, and point-by-point responses associated with peer-review at Life Science Alliance will be published, alongside the manuscript. If you do want to opt out of having the reviewer reports and your point-by-point responses displayed, please let us know immediately.

IMPORTANT: If you will be unreachable at any time, please provide us with the email address of an alternate author. Failure to respond to routine queries may lead to unavoidable delays in publication.

Scheduling details will be available from our production department. You will receive proofs shortly before the publication date. Only essential corrections can be made at the proof stage so if there are any minor final changes you wish to make to the manuscript, please let the journal office know now.

DISTRIBUTION OF MATERIALS:
Authors are required to distribute freely any materials used in experiments published in Life Science Alliance. Authors are encouraged to deposit materials used in their studies to the appropriate repositories for distribution to researchers.

You can contact the journal office with any questions, contact@life-science-alliance.org

Again, congratulations on a very nice paper. I hope you found the review process to be constructive and are pleased with how the manuscript was handled editorially. We look forward to future exciting submissions from your lab.

Sincerely,

Sarita Hebbar, PhD
Scientific Editor
Life Science Alliance
http://www.lsajournal.org
